# Effect of the COVID-19 pandemic on the proportion of physically active children and adults worldwide: A systematic review and meta-analysis

**DOI:** 10.3389/fpubh.2022.1009703

**Published:** 2022-12-09

**Authors:** Karima Chaabna, Sonia Chaabane, Anupama Jithesh, Sathyanarayanan Doraiswamy, Ravinder Mamtani, Sohaila Cheema

**Affiliations:** Institute for Population Health, Weill Cornell Medicine - Qatar, Education City, Qatar Foundation, Ar-Rayyan, Qatar

**Keywords:** physical activity, physical inactivity, movement restrictions, COVID-19 pandemic, prevalence of physical activity

## Abstract

**Introduction:**

Safety measures implemented to address the COVID-19 pandemic have had a profound impact on the mobility of people worldwide We synthesized the global evidence on physical activity (PA) participation before and during the pandemic.

**Methods:**

We conducted a systematic review, searching PubMed, Embase, WHO Global literature on coronavirus disease (between January 2020 and April 2022), and reference lists. Meta-analysis and meta-regression were conducted to quantitatively synthesize the data.

**Results and discussion:**

Sixty-three primary studies were included. In children, the global pooled prevalence of PA was 46.4% before the pandemic, 40.6% during the pandemic before movement restriction (MR), and 19.5% during MR. A statistically significant decrease in prevalence was observed between the period before the pandemic and the period during which MR was implemented (*p* < 0.001). In adults, the global pooled prevalence (both sexes) decreased between the periods before the pandemic (64.7%) and during MR (57.0%). During the period of COVID-19 MR, children had significantly lower odds to meet the WHO PA recommendation than adults (19.5%, 95%CI: 15.8–23.8% vs. 57.0%, 95%CI: 43.3–62.5%; OR = 0.21; *p* ≤ 0.001). Patient populations were less active than the general population, and their PA levels decreased during the pandemic. Mental and physical health benefits of PA have been well-demonstrated. Prioritizing PA in health campaigns and strategies is critical to address health issues exacerbated during this pandemic.

**Protocol registration:**

doi: 10.17605/OSF.IO/GVABX.

## Introduction

The ongoing COVID-19 pandemic has caused worldwide human devastation, with over five hundred million cases and six million deaths as of May, 2022 (1). A wide range of strategies have been employed to mitigate the pandemic. Movement restrictions (MRs), especially when combined with other safety measures such as handwashing and wearing masks, have been reported to reduce the number of COVID-19 cases and deaths (2–7). In January 2020, China implemented MRs to limit viral spread (e.g., city lockdowns, school closures, home quarantine requirements, and travel restrictions) (8). Similarly, complete or partial MR measures were adopted by more than 100 countries by the end of March 2020 (9).

COVID-19-related MR measures resulted in a drastic change in people's daily activities, and may have had a correspondingly wide effect on physical activity (PA) levels. Our hypothesis is that these measures negatively affected PA levels among children and adults in both the general and patient populations worldwide. To be considered physically active, the World Health Organization (WHO) guidelines recommend an average of 60 min (min) per day of moderate-to-vigorous intensity PA for children (10). Adults should aim for either 150–300 min/week of moderate-intensity aerobic PA, 75–150 min/week of vigorous-intensity aerobic PA, or an equivalent combination of moderate- and vigorous-intensity PA throughout the week for optimum health benefit (10). Prior to the COVID-19 pandemic, the global prevalence of individuals who achieved these levels of activity was 72.5–77% (11–13) among adults and 20% among the younger population. As a result of MR measures incorporated during the pandemic, it is likely that fewer adults and children were able to satisfy the WHO PA recommendations.

Previously published reviews highlighted a decrease in PA time per day (14, 15) and in energy expenditure and step count per week during the pandemic (16, 17). However, none of these reviews quantified the prevalence of physically active individuals during the pandemic as per the WHO PA guidelines, nor the changes in PA prevalence when compared to pre-pandemic numbers. Other reviews on PA during the pandemic were restricted to patients with clinical conditions [e.g., non-communicable diseases (18) and neurological diseases (19)] or university students (20). The objectives of this systematic review (SR) and meta-analysis are to: (1) synthesize the available evidence from published primary studies on the worldwide prevalence of physically active people before and during the COVID-19 pandemic, (2) quantify country-specific PA prevalence measures and assess demographic specificities among the general and patient populations, and (3) analyze variations in PA prevalence before the COVID-19 pandemic; during COVID-19 pandemic before MRs; and during the COVID-19 pandemic with MRs.

## Method

The protocol of this research project was developed *a priori* and registered on the Open Science Framework (registration DOI 10.17605/OSF.IO/GVABX). The SR methodology was developed based on the Guidance on Conducting Systematic Reviews, and Meta-analysis of Observational Studies of Etiology (COSMOS-E), AMSTAR2 guidelines (21), and the Cochrane Handbook for Systematic Reviews of Interventions (22). The SR reporting follows the Preferred Reporting Items for Systematic Reviews and Meta-Analyses (PRISMA 2020) guidelines (23) (Supplementary Tables 1, 2 and Supplementary Figure 1).

### Population of interest

The population of interest was composed of the general and the patient populations of all age groups belonging to any country. Children were defined as persons being younger than 18 years of age and adults were considered as those being 18 years of age and above. The patient population included patients with COVID-19 in addition to patients with other health conditions.

### Outcome of interest

PA is defined as any movement of the body produced by skeletal muscles that requires energy expenditure. The primary outcome of interest was PA prevalence during the COVID-19 pandemic, which is the proportion of individuals being sufficiently physically active as per the WHO PA guidelines (10). Validated questionnaires—e.g., the International Physical Activity Questionnaire (IPAQ) and the Global Physical Activity Questionnaire (GPAQ)—are used to assess PA levels among adults and categorize PA levels as high, moderate, or low according to the energy expenditure per week [measured in metabolic equivalent of task (MET)-min/week]. Adults at high PA level are those practicing: (i) vigorous-intensity activity on at least 3 days and accumulating at least 1,500 MET-min/week; or (ii) any combination of walking, moderate-intensity, or vigorous-intensity activities on seven days and achieving a minimum of 3,000 MET-min/week. Adults at moderate PA level are those practicing: (i) vigorous activity of at least 20 min/day on three or more days; or (ii) moderate-intensity activity or walking of at least 30 min/day on 5 or more days; or (iii) any combination of walking, moderate-intensity or vigorous-intensity activities on 5 or more days and achieving a minimum of 600 MET-min/week. Adults at low PA level are those not meeting the WHO criteria for high and moderate PA levels, and are therefore considered physically inactive.

**Figure 1:**
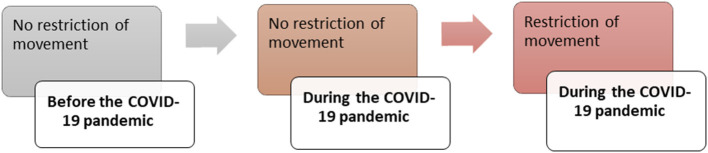
Physical activity before and during the COVID-19 pandemic. Movement restriction in the included studies was due to lockdown, home confinement, community quarantine, or physical distancing policies combined with school closure.

For all countries except China, the period “during the COVID-19 pandemic” began when the WHO characterized the outbreak as a pandemic in March 2020 (9). However, for China, a public health emergency was declared in January 2020 (24) and MR was implemented (8). Thus, we considered the period “during the COVID-19 pandemic” starting in January 2020. During the pandemic, prevalence was estimated for the period prior to MR measures and also while MR measures were in place (Figure 1). The secondary outcome of interest was PA prevalence before the COVID-19 pandemic. PA prevalence before the COVID-19 pandemic was included only if the included studies also reported PA prevalence data during the pandemic. This prevalence has been estimated using data either collected by primary studies conducted in 2019 or by studies conducted during the pandemic based on participants' recall (self-reported) of their PA level before COVID-19. Additionally, variations in PA prevalence before and during MR in the COVID-19 pandemic were also assessed.

### Selection criteria

All observational studies (e.g., cross-sectional or cohort studies) reporting prevalence measures or data that allow calculation of prevalence measures of PA or physical inactivity during the pandemic were included (i.e., PA prevalence could be deduced from the prevalence of physical inactivity). Studies quantifying the proportion of people by levels of total PA weekly time or total energy expenditure of weekly PA (MET-min/week) as per the WHO PA guidelines were also included. Studies that reported data only on average step count, average PA duration, difference in mean time, or sedentary behavior were excluded.

Viewpoints, reviews, pre-prints, and commentaries were excluded. Studies reported in records written in languages other than Arabic, English, French, and/or Urdu—the languages spoken by the research team—were excluded if the information provided in their English abstracts did not meet the inclusion criteria. Studies reporting only PA prevalence before the COVID-19 pandemic were also excluded.

### Literature search strategy

The literature search was developed per the AMSTAR2 guidelines (21). The database selection and the search strategy were reviewed by an experienced librarian. The search was conducted on April 25, 2022. To ensure a comprehensive search (25, 26), PubMed, EMBASE, and WHO Global Literature on Coronavirus Disease were searched for gray and non-gray literature. The search strategy was developed by combining the two main concepts of our research question–PA and COVID-19. The detailed search strategy for each database is reported in Supplementary Box 1. The literature search was not limited to any language, country, or study design. The reference lists of the included primary studies and identified reviews were also searched for additional references.

### Multi-stage screening and data extraction

After removal of the duplicate records, two reviewers independently conducted title and abstract screening, full-text screening, and data extraction. Discrepancies in study selection and data extraction were resolved at the end of each step in consultation with the other reviewers to achieve a consensus on study inclusion and data extraction. Duplicate removal and multi-stage screening were conducted using the online SR software, Rayyan (Rayyan Systems Inc., Cambridge, MA, USA, https://www.rayyan.ai/). Data extraction was conducted using a standardized extraction sheet developed on Microsoft Excel after piloting on a small sample of studies.

Data was extracted from each included primary study for the following variables: (i) study characteristics (study design, data collection time, MR status, sampling method, and response rate); (ii) country; (iii) setting; (iv) population demographics (sex, age, and population description); (v) outcome (prevalence, sample size, PA level, and instrument).

### Quality Assessment, reporting bias, and certainty assessment

We utilized a published quality assessment checklist to assess primary study-level methodological quality. The checklist was adapted for primary studies evaluating PA levels and developed based on the Population, Intervention, Comparator, Timing, and Setting (PICOTS) framework and other published assessment checklists (27–32). The quality assessment checklist helped assess each study's risk of bias, sensitivity (ability to detect a true effect), and reporting bias. Included quality assessment domains were: population characteristics, outcome definition, measurement tool, setting, timing, sampling method, and response rate (Supplementary Table 3). No quality assessment summary score was computed as per COSMOS-E guidance (33). Studies were classified according to the level of risk of bias for each bias domain (low, moderate, or high risk) (22, 33). Primary studies were appraised qualitatively by one reviewer and checked by another. Any discrepancies were resolved by discussion with the consultation of a third reviewer.

Reporting bias due to missing data was discussed. Confidence in the body of evidence presented in the SR was assessed by evaluating the validity and reliability of our estimates. The certainty assessment method was based on the Grading of Recommendations Assessment, Development and Evaluation (GRADE) approach, which considers the risk of bias and reporting biases in a body of evidence, precision of the meta-analysis effect estimates (pooled PA prevalence), the consistency of the primary study results, and how directly the body of evidence answers the research question.

### Qualitative synthesis

Findings of all included primary studies were synthesized narratively.

### Quantitative synthesis

When PA prevalence was reported as levels of “high”, “moderate”, or “low”, as per the scoring of questionnaires such as IPAQ and GPAQ, the prevalence of physically active individuals was calculated by combining the prevalence of high and moderate PA levels.

When PA prevalence was reported as levels of “moderate-to-vigorous”, “moderate-”, and “vigorous-intensity”, we chose to incorporate only the “moderate-to-vigorous” intensity PA prevalence, because it combined all three categories of active people: those engaging in only moderate-intensity PA, those engaging in only vigorous-intensity PA, and those engaging in a combination of moderate- and vigorous-intensity PA.

Meta-analysis was conducted to quantitatively synthesize the data by combining two or more prevalence measures. Clopper-Pearson confidence intervals were computed for individual prevalence measures. Prevalence measures and their 95% confidence intervals (95% CI) were pooled based on the random-effects model with the logit transformation of the proportion. To be included in the meta-analysis, there was a minimum study sample size of 25(34). Explicit timing of the PA prevalence must have been reported to be included as a prevalence measure in the meta-analysis (e.g., before the pandemic, during the pandemic prior to MR implementation, or during the pandemic with MR).

We assessed the heterogeneity between studies using the *I*^2^ statistic, which describes the percentage of variability across studies that is due to true heterogeneity rather than chance (35). Heterogeneity between studies was considered as substantial when *I*^2^ > 50% (22). To investigate reasons for heterogeneity between studies, subgroup meta-analysis was conducted to estimate pooled PA prevalence by country, sex, and age. Cochran's Q between-subgroups statistic was used to test for differences between prevalence estimates across subgroups (36).

To further explore heterogeneity between studies, univariate random-effects meta-regression was conducted to evaluate potential associations between PA prevalence and study characteristics (i.e., instrument and study setting). Meta-regression was also used to estimate odds ratios (ORs) and conduct *t*-tests to assess PA prevalence change between the periods before the pandemic and during MR, as well as PA prevalence variation between children and adults.

Potential small study or publication biases were examined using funnel plots and Peters test for asymmetry (37).

Statistical significance was considered at *p* ≤ 0.05. Meta-analyses and meta-regressions were conducted using R software (version 64 4.0.0) developed by the Comprehensive R Archive Network (https://cran.r-project.org/).

## Results

### Study selection

The literature search conducted in the electronic databases identified 3,405 records (Supplementary Figure 1). The citation hand searching identified 275 primary studies. After duplicate removal from all searches and multi-stage screening, 63 primary studies were included in both the SR and the meta-analysis. Excluded studies at full-text screening stage are listed in Supplementary Box 2.

### Study characteristics

Characteristics of the individual studies on PA prevalence worldwide before and during the pandemic are described in three tables reporting information about the adult general population, the child general population, and the patient population (Supplementary Tables 4–6). Most of the included studies were cross-sectional (78%, 49/63), ten were longitudinal, and four were prospective cohort studies.

### Study-level quality assessment

Overall, most included primary studies were of good methodological quality (low risk of bias; Supplementary Table 7). All included studies used the definition of the population sufficiently active as per the recommended WHO guidelines and clearly defined their study setting and population demographics (sex, population type, and age). Most studies (90.5%, 57/63) clearly defined data collection timing—whether it was before the pandemic or during the pandemic, and specified whether MR has been implemented or not. Validated measurement tools for PA were used in 77.8% (49/63) of studies. Non-validated questionnaires were used in 6.3% (4/63) of the studies, and measurement methodology was not reported in 10 primary studies. Most studies (74.6%, 47/63) used a non-random sampling method (convenient sampling) while 9.5% (6/63) of the studies used a random sampling method. A total of 11 studies did not report their sampling method (17.5%). Response rate was clearly reported in 27% (17/63) of the studies. Among the studies reporting a response rate, 70.6% (12/17) had an acceptable (50–79%) to a good (≥80%) response rate.

### General population—Children

A total of 51 PA prevalence measures were retrieved from 16 studies conducted among children aged three and above (Supplementary Table 4). PA prevalence estimation was conducted in 28 countries (Supplementary Table 4). Prior to the pandemic [between September 2019 and February 2020, number of prevalence measures (*n*) = 11], PA prevalence (both sexes) varied between 18.9% in Germany to 77.2% in Spain. During the pandemic, prior to MR (*n* = 8), PA prevalence varied from 14.3% in Italy to 81.1% in Poland. During MR (*n* = 32), PA prevalence varied from 7.0% in Brazil to 66.9% in Poland.

**Figure 2:**
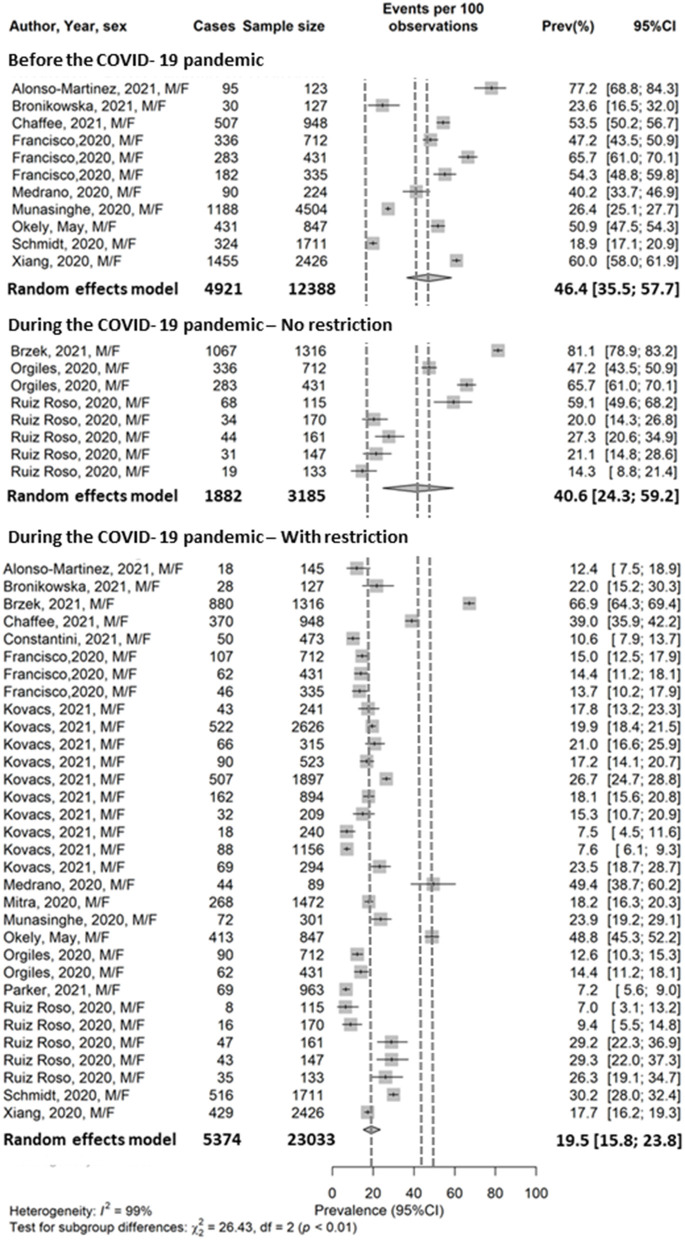
Pooled prevalence of physical activity in the child general population. Francisco et al. (38) reported PA data from Italy, Spain, and Portugal. Orgilés et al. (39) reported PA data from Italy, and Spain. Ruiz-Roso et al. (40) reported PA data from Brazil, Chile, Colombia, Italy, and Spain. Kovacs et al. (41) reported PA data from France, Germany, Hungary, Italy, Poland, Portugal, Romania, Russia, Slovenia, Spain.

Meta-analysis estimated that the global pooled PA prevalence among children was 46.4% prior to the pandemic, 40.6% during the pandemic before MR, and 19.5% during MR (Figure 2). A non-statistically significant decrease of PA prevalence was identified between the period prior to the pandemic and the period during the pandemic before MR. However, statistically significant decrease was identified between the period prior to the pandemic and the period during MR (Q between subgroup test *p* ≤ 0.001). The meta-regression estimated that children had *lower* odds for being active during MR than before the pandemic (OR = 0.28, *p* ≤ 0.001). Additionally, the meta-regression estimated that children had *lower* odds of meeting the WHO PA guidelines than adults during MR (OR = 0.21, *p* ≤ 0.001).

### General population—Adults

A total of 117 PA prevalence measures were retrieved from 43 studies conducted among the adult general population in 33 countries from five continents (Supplementary Table 5). One study included 29 countries located in “Asia, America, Africa, and Europe” but did not specify these countries (42).

Prior to the pandemic (*n* = 42), PA prevalence varied between 30.1% in Brazil and 96.9% in Mexico (both sexes). One study (43) conducted during the pandemic and prior to MR, reported a PA prevalence of 31.2% in Brazil (both sexes). During MR (*n* = 73), PA prevalence varied between 2.9% among females in Brazil and 96.9% in Mexico (both sexes). Two studies (44, 45) have reported PA data during the pandemic with MR among elderly aged 55 or above, ranging between 38.7% in females to 65.2% (both sexes). One study (46) conducted during the pandemic and *after* to MR, reported a PA prevalence of 30.7% in India (both sexes).

Meta-analysis showed a decrease in the global pooled PA prevalence (both sexes) between the period before the pandemic (64.7%) and the period during MR (57.0%) (Table 1). However, the difference in PA prevalence between the periods was not statistically significant.

**Table 1:** Pooled prevalence of physical activity in the adult general population.

	**Number of prevalence measures**	**Total sample size**	**Prevalence range (%)**	**Effect size**		**Subgroup Comparison (Q between subgroup tests *p*-value)**	**Heterogeneity between studies *I*^2^ (%)**

				**Weighted average prevalence (%)**	**95% CI**		
**Global physical activity prevalence in adults**							
Before the COVID-19 pandemic	23	78,770	30.1–96.9	64.7	54.6–74.1	0.0987	99.8
During the COVID-19 pandemic with movement restriction	44	133,982	6.9–96.9	57.0	43.3–62.5		99.9
**Country-level physical activity prevalence in adults**							
Australia	3	6,427	33.0–90.4	57.2	32.3–80.3	0	99.6
Bangladesh	1	2,028	62.1	62.1	60.0–64.2		N/A
Brazil	3	46,885	7.7–23.3	13.8	11.7–12.3		98.4
Canada	2	2,153	36.6–37.5	37.1	35.0–39.1		0
China	5	14,482	29.6–58.8	43.7	34-7–52.8		97.7
Greece	1	8,495	57.6	57.6	56.5–58.6		N/A
Iran	1	670	21.9	21.9	18.9–25.2		N/A
Ireland	1	903	91.14	91.1	89.1–92.8		N/A
Israel	1	473	83.1	83.1	79.6–86.3		
Italy	3	4,756	44.7–75.1	60.3	44.9–74.8		99.1
Mexico	2	69	48.6–96.9	77.3	21.3–100.0		95.8
Morocco	1	406	30.0	30.0	25.7–34.6		N/A
New Zealand	1	4,007	92.4	92.4	91.5–93.2		N/A
Portugal	2	7,260	66.5–68.2	67.0	65.5–68.4		29.2
Spain	6	5,740	39.9–75.0	54.6	47.9–61.2		94.2
Switzerland	1	76	75.0	75.0	64.6–84.2		
Tunisia	1	216	39.4	39.4	32.9–46.0		N/A
Turkey	1	2,301	6.9	6.91	5.9–8.0		N/A
United Kingdom	3	7,144	9.1–90.9	54.5	3.0–99.3		100
Ukraine	1	1,512	43.2	43.2	40.7–45.7		N/A
USA	1	1,809	76.0	76.0	74.0–77.9		

Year of data collection was 2020.

A significant difference of PA prevalence in adults was demonstrated between countries. Pooled prevalence ranged from 6.9% in Turkey to 96.9% in Mexico during MR (Table 1). More males appeared to be active than females before and during the pandemic even during MR (Table 2).

**Table 2:** Sex differences in physical activity prevalence before and during the COVID-19 pandemic in the adult general population.

	**Number of prevalence measures**	**Total sample size**	**Prevalence range (%)**	**Effect size**		**Subgroup Comparison (Q between subgroup tests *p*-value)**	**Heterogeneity between studies *I*^2^ (%)**
				**Weighted average prevalence (%)**	**95% CI**		
**Before the COVID-19 pandemic**							
Male	9	27,876	33.0–82.9	67.8	49.0–84.1	0.4366	99.8
Female	9	37,168	27.6–76.5	57.9	40.7–74.2		99.9
**During the COVID-19 pandemic with movement restriction**							
Male	13	38,164	4.8–84.0	41.7	31.8–52.0	0.5691	99.9
Female	13	42,674	2.9–64.7	38.5	23.4–54.7		99.9

Year of data collection was 2020.

A total of seven PA prevalence measures on mixed populations of adults and children were identified before and during the pandemic with MR. We identified one PA prevalence measure conducted during the COVID-19 pandemic *after* MR was lifted (Supplementary Table 4).

### Patient population—COVID-19 patients

Two prevalence measures were retrieved from one study conducted among adult COVID-19 patients in Spain (47) (Supplementary Table 6). PA prevalence varied between 17.6% during MR and 88.2% before MR in Spain (both sexes). No study measured PA prevalence among COVID-19 patients before their diagnosis and quarantine period.

### Patient population—Non-COVID-19 patients

Six prevalence measures were retrieved from five studies conducted among adult non-COVID-19 patients during the pandemic (Supplementary Table 6). Prior to MR, PA prevalence was reported in Brazil at 24.5% (April-May 2020) among patients with depression and in Italy (January-February 2020) at 62.5% among patients visiting a department of patient medicine and surgery. During MR, PA prevalence (0.0–71.4%) was reported in Bangladesh, Iraq, Italy, and Spain among patients with “chronic disease”, multiple sclerosis, unusual frequent urination associated with abnormal sleep, and patients visiting a department of patient medicine and surgery. None of these prevalence measures were among intensive-care or bed-ridden patients. No data was identified for the period prior to the pandemic.

Six PA prevalence measures among the non-COVID-19 patient population were included in the meta-analysis. During the pandemic, pooled PA prevalence was 42.1% before MR, and this decreased to 25.5% during MR. No statistically significant difference between the two periods with and without MR was identified.

No data was identified for the child patient population.

### Heterogeneity

High heterogeneity between studies was detected when conducting meta-analysis (*I*^2^ > 90%). Sub-group meta-analysis (Tables 1, 2) and meta-regression (Table 3) identified the following factors as significant (*p* < 0.05) in explaining some of the variability between studies among adults: country, instruments used to measure PA levels, sample size, and sampling method. In addition to the instruments used to measure PA levels, study setting and design were identified as significant (*p* < 0.05) in explaining some of the variability between studies among children (Table 3).

**Table 3:** Univariate meta-regression models for physical activity prevalence in adult and child general populations.

		**Before COVID-19 pandemic**				**During COVID-19 pandemic, with restriction**			
		**No. of prevalence measures**	**Weighted prevalence**	**OR**	***p*-value**	**No. of prevalence measures**	**Weighted prevalence**	**OR**	***p*-value**
		**95% CI**	**95% CI**			**95% CI**	**95% CI**	
**Adults**									
Instruments	Validated	18	72.2	Ref.		56	62.1	Ref.	-
			63.3–79.6				24.6–36.7		
	Non-validated	1	35.3	0.2	0.054	3	54.3–69.2	0.3	0.475
			32.8–37.9	0.04–1.02			24.6–36.7	0.1–0.99	
	NR	4	42.2	0.3	**0.004**	8	39.8	0.4	0.347
			29.6–55.9	0.1–0.7			27.9–53.1	0.2–0.9	
Study setting	Community	5	67.2	Ref.	-	12	61.4	Ref.	-
			43.9–84.3				40.0–79.1		
	Online	18	65.8	0.96	0.931	53	57.6	0.9	0.775
			55.2–75.1	0.4–2.6			49.9–65.0	0.4–1.9	
	Online and offline	0	-	-	-	2	52.3	0.7	0.717
			-	-			32.3–71.5	0.1–4.3	
Study design	Cross-sectional	19	64.3	Ref.	-	57	56.4	Ref.	-
			53.3–73.9				48.3–64.2		
	Longitudinal	4	74	1.6	0.365	10	66.0	1.6	0.281
			56.9–86.0	0.6–4.8			54.3–76.1	0.7–3.5	
Sample size	>1,000	10	55.4	Ref.	-	33	50.9	Ref.	-
			43.6–66.6				41.6-60.2		
	101–1,000	9	69.1	1.8	0.136	25	59.2	1.4	0.2666
			53.9–81.1	0.8–3.9			47.4–70.0	0.8–2.5	
	≤ 100	4	83.1	4.2	**0.011**	9	78.5	3.7	**0.004**
			68.0–91.9	1.4–12.9			64.9–87.8	1.5–7.5	
Sampling method	Probabilistic	0	-	-	-	2	18.4	Ref.	-
			-				5.4–47.3		
	Non-probabilistic	20	65.63	Ref.	-	58	58.9	6.4	**0.026**
			55.0–74.9				51.2–66.1	1.3–32.4	
	NR	3	67.3	1.2	0.781	7	61.8	7.6	**0.029**
			48.5–81.8	0.4-4.1			46.8–74.9	1.2–47.3	
**Children**									
Instruments	Validated	12	34.9	Ref.	-	36	24.0	Ref.	-
			24.4–47.0				19.4–29.3		
	Non-validated	7	60	2.8	**0.005**	15	36.2	1.8	**0.044**
			50.5–68.9	1.4–5.7			23.5–51.1	1.0–3.2	
	NR	0	-	-	-	1	18.2	0.7	0.716
			-				16.3–20.3	0.1–4.6	
Study setting	Community	7	50.7	Ref.	-	15	36.2	Ref.	-
			32.4–68.9				24.6–49.7		
	Online	12	40.3	0.7	0.320	37	23.8	0.6	**0.041**
			29.9–51.6	0.3–1.5			19.0–29.5	0.3–0.98	
Study design	Cross-sectional	11	42.5	Ref.	-	35	22.8	Ref.	-
			31.0–54.9				18.1–28.3		
	Longitudinal	4	32.2	0.6	0.359	9	29.7	1.4	0.292
			20.8–46.3	0.03–1.7			19.9–41.9	0.7–2.8	
	Cohort	4	60.9	0.7	0.129	8	46.8	3.0	**0.002**
			37.0–80.4	0.5–1.2			28.6–65.9	1.5–6.0	
Sample size	>1,000	4	46.11	Ref.	-	11	31.2	Ref.	-
			21.6–72.7				19.1–46.7		
	101–1,000	15	43.5	0.9	0.836	40	25.5	0.8	0.397
			33.5–54.1	0.3–2.5			20.4–31.5	0.4–1.5	
	≤ 100	0	-	-	-	1	49.4	2.2	0.458
			-				39.2–59.7	0.3–16.3	
Sampling method	Probabilistic	2	50.6	Ref.	-	4	40.2	Ref.	-
			37.0–64.1				24.6–58.1		
	Non-probabilistic	12	39.4	0.6	0.514	38	22.8	0.4	0.084
			28.7–51.2	0.2–2.4			18.2–28.0	0.2–1.1	
	NR	5	53.1	1.1	0.874	10	41.5	1.1	0.923
			30.2–74.7	0.3–4.8			26.3–58.5	0.4–3.0	

The bold values are statistically significant results (*p* ≤ 0.05).

### Reporting bias and certainty of evidence

Overall, most of the included primary studies reported the information required to allow proper quality assessment. However, despite no statistically significant asymmetries were identified in the funnel plots exploring publication bias in the meta-analyses for adults and children (Supplementary Figures 1, 2, Peters test *p* = 0.2658 and 0.8041 in adults and children, respectively), our synthesis may have a reporting bias due to the limited number of primary studies conducted and their geographical coverage, even though coverage included 5 continents (48).

The overall risk of bias was assessed to be low among the primary studies, as the internal validity of the included primary studies was relatively good. However, identified heterogeneity between studies due to statistically significant differences between countries and study methodology has likely impacted the precision of the estimated pooled global prevalence. Consequently, this might explain the non-significant change observed between the two periods: prior to the pandemic and during MR. Considering that the lower PA prevalence during the pandemic period with MR is consistently observed in population subgroups, a true decrease in PA prevalence between these two periods is likely. Consequently, it appears that the COVID-19 pandemic has presumably negatively impacted the global prevalence of physically active people.

## Discussion

To summarize, we observed that in 2020, just prior to the COVID-19 pandemic, about two-thirds of the global adult population (65%) were physically active as per the WHO PA guidelines. This SR demonstrates that there was a decreasing trend in the proportion of physically active adults and children following MR as compared to the pre-COVID-19 era. Furthermore, the decrease in PA prevalence appears to have been more substantial among children than in adults, and was statistically significant. During the pandemic, our SR demonstrated that children had significantly lower odds to meet the WHO PA recommendations as compared to adults, which is consistent with the published data prior to the pandemic (12). The lower participation in PA among females as compared to males previously observed before the pandemic (13, 49, 50), was also demonstrated in our analyses during the pandemic. The pre-COVID-19 differences observed that the prevalence of people meeting the WHO PA guidelines by age group (children vs. adults) and sex have been persistent during the pandemic (11, 13, 51, 52). Additionally, the adult non-COVID-19 patient population was less active than the general population before and during the pandemic. Limited data was available for COVID-19 patients, allowing no conclusion to be made about changes in the PA prevalence in this population. Previously published data revealed a significant reduction in PA levels during the pandemic among patients with clinical diseases (18, 19). The evidence synthesized in this SR also suggests a decline in the prevalence of physically active adult patient populations during the pandemic. Overall, this study demonstrates that the reduction of PA prevalence was likely exacerbated by MR, which were implemented to prevent and control the spread of SARS-CoV-2 virus.

While MR may have contributed to reducing the number of COVID-19 cases and deaths, they appear to have negatively impacted PA levels among children and adults. Prevalence patterns of people meeting the WHO PA guidelines by age group (adults vs. children) and sex during the pandemic are likely due to the persistence of previously identified barriers to PA, such as lack of time, social support, and motivation. It has been demonstrated that personal and social barriers to PA are higher among females than males in several countries (13, 53–57). These barriers include the traditional roles and family obligations ascribed to women, as well as a lack of social support, less freedom, and less access to facilities to engage in PA as compared to males. Concerns and insecurities about stereotypes, body image, and cultural acceptability are some of the sociocultural factors that have been identified to explain lower PA prevalence among adult females (49, 50). During the pandemic, MR is likely to have led to additional barriers to PA, as work and transport-related PA was interrupted for many individuals when they shifted to working from home and to obtaining groceries and household supplies through home delivery. Indoor sport and fitness facilities were also closed, and outdoor PA was limited (58, 59). Additionally, barriers to PA may have been worsened by school and childcare closures for females in particular. Efforts to address the lower PA prevalence in females during the COVID-19 pandemic and beyond may start with better investment and attention to addressing the current sociocultural norms.

Maintaining sufficient levels of PA positively impacts an individual's physical (60, 61) and mental health and quality of life (62–65). Considering this, our findings that patient populations are generally less active than the general population, and that their PA levels further decreased during the COVID-19 pandemic, are of serious concern. Now, more than ever, PA should be proactively recommended to the general and patient populations as an effective and affordable non-pharmaceutical intervention to prevent and treat physical and mental health conditions. This recommendation is more relevant during the COVID-19 pandemic and post-COVID-19 era because of the reported increase in non-communicable diseases for which PA can be beneficial (66–73). Healthcare professionals should be mindful of the mental and physical impact of MR on the wellbeing of their patients. Targeting vulnerable populations, such as the elderly and patients with non-communicable diseases, to promote PA during the pandemic (especially during MR) and beyond can significantly reduce disease burden.

To our knowledge, this is the most comprehensive and up to date SR and meta-analysis covering both general and patient populations that assesses PA prevalence during the pandemic according to main determinants of PA worldwide. The country-, period-, age-, and sex-specific pooled PA prevalence measures are a consequential addition to the evidence on PA participation during the pandemic. Although the WHO PA guidelines for the general and patient populations are the same, we have separately assessed the prevalence of active people in the patient and general populations to take into account the potential impact of health conditions on PA participation. This compilation will serve as a benchmark for epidemiologists and those planning public health interventions. Overall, the quality of the primary studies included in our SR was good as reflected by their internal validity. All included studies have used the standard definition for being sufficiently active as per the WHO PA guidelines. Most of the included primary studies have used a validated instrument for measuring PA participation, which reinforces the robustness of our findings. Even though our SR search strategy was inclusive of all countries worldwide, our findings may not be generalized to the global population. This is due to the fact that primary studies assessing PA levels in the general and patient populations were conducted in a limited number of countries during the COVID-19 pandemic, which may not be representative of the all 195 countries (48). Additionally, PA prevalence reported in our SR as country-level prevalence may not be generalizable to the entire country since a limited number of prevalence measures was available. Limited numbers of prevalence measures could be explained by the relatively short data collection period (spring to summer 2020) when the studies were performed. However, some PA prevalence measures were based on national coverage surveys and probabilistic sampling, which allows a better generalizability of the results at country-level. Although most countries followed global recommendations on MR during the early phase of the COVID-19 pandemic, country-specific differences in the coverage or number of restrictive measures could exist, and these differences can impact the observed PA participation. Although data on PA participation prior to the pandemic was collected prospectively in some studies, in some others, this data was collected retrospectively using self-reported PA levels, which can introduce recall bias.

Although several subgroup and meta-regression analyses were performed to explore and reduce heterogeneity, the latter remains relatively high in some groups. This could be explained by other methodological differences such as data collection processes, cultural factors, setting, policy factors, or significant differences in PA participation between countries. Identified heterogeneity has likely impacted the precision of the estimated pooled global prevalence. Consequently, this might have resulted in the statistically non-significant change observed between the studied periods; however, a true decrease in PA prevalence is likely. We have observed consistency in the direction of the PA prevalence change. Thus, it appears that the COVID-19 pandemic has likely negatively impacted the global prevalence of physically active people.

## Conclusion

The COVID-19 pandemic has noticeably changed people's lifestyles. The evidence synthesized in this SR suggests a decline in the proportion of physically active children and adults in both the general and patient population during the COVID-19 pandemic. Lower PA prevalence observed in adult females and children is of concern. The SR demonstrated that the reductions of PA prevalence are likely exacerbated by MR measures, which were implemented to prevent and control the spread of the SARS-CoV-2 virus. This decline in PA participation may increase individual vulnerability to mental and physical health conditions. PA can improve the health of individuals suffering from non-communicable disease (e.g., cardiovascular disease, type 2 diabetes, and mental health conditions), many of which are associated with a higher risk of severe COVID-19. More studies using reliable measurement tools for assessing PA participation in low and middle-income countries are needed. Promoting PA surveillance utilizing standard measurement tools consistently at national and international levels is essential to allow meaningful comparisons and to implement effective evidence-based interventions.

## Data availability statement

The original contributions presented in the study are included in the article/Supplementary material, further inquiries can be directed to the corresponding author/s.

## Author contributions

KC, SCha, SD, RM, and SChe collectively contributed to the conception of the study and involved in the literature search. KC, SCha, RM, SChe, and AJ were involved in the screening and extraction steps. KC, SCha, and AJ were involved in table preparation. KC and SCha implemented the statistical analysis and manuscript drafting. All authors read, reviewed the manuscript, and approved its final version.
