# Supplementary material for: Effect of the COVID-19 pandemic on the proportion of physically active children and adults worldwide: A systematic review and meta-analysis
Source: Front Public Health. 2022 Dec 9;10:1009703. doi: 10.3389/fpubh.2022.1009703 (PMC9780669; doi:10.3389/fpubh.2022.1009703)
Supplement: Supplementary file 1 [file Data_Sheet_1.docx]

**Effect of the COVID-19 pandemic on the proportion of physically active children and adults worldwide: a systematic review and meta-analysis**

**Karima Chaabna^1*^, Sonia Chaabane^1^, Anupama Jithesh^1^, Sathyanarayanan Doraiswamy^1^, Ravinder Mamtani^1^, Sohaila Cheema^1^**

^1^Institute for Population Health, Weill Cornell Medicine – Qatar, Education City, Qatar foundation, P.O.Box. 24144, Qatar

***Correspondence:**

Dr. Karima Chaabna

kac2047@qatar-med.cornell.edu

Table S1 PRISMA checklist 2020

Table S2: PRISMA checklist for abstract

Figure S1: PRISMA flow chart 2020

Box S1: Search strategy

Table S3: Quality assessment (QA) checklist for the original studies

Box S2: List of excluded studies

Table S4: Prevalence of Physical activity among adults during COVID-19

Table S5: Prevalence of Physical activity among adults with clinical conditions

Table S6: Physical activity prevalence among children during COVID-19

Table S7: Quality assessment of the included studies

Figure S1: Funnel plot for the during pandemic meta-analysis among adults

Figure S2: Funnel plot for the during pandemic meta-analysis among children

# **Table S1 PRISMA checklist 2020**

| **Section and Topic** | **Item #** | **Checklist item** | **Location where item is reported** |
| --- | --- | --- | --- |
| **TITLE** | | |  |
| Title | 1 | Identify the report as a systematic review. | Page 1 |
| **ABSTRACT** | | |  |
| Abstract | 2 | See the PRISMA 2020 for Abstracts checklist. | Page 2 |
| **INTRODUCTION** | | |  |
| Rationale | 3 | Describe the rationale for the review in the context of existing knowledge. | Pages 3-4 |
| Objectives | 4 | Provide an explicit statement of the objective(s) or question(s) the review addresses. | Page 4 |
| **METHODS** | | |  |
| Eligibility criteria | 5 | Specify the inclusion and exclusion criteria for the review and how studies were grouped for the syntheses. | Pages 6-7 |
| Information sources | 6 | Specify all databases, registers, websites, organisations, reference lists and other sources searched or consulted to identify studies. Specify the date when each source was last searched or consulted. | Page 6 |
| Search strategy | 7 | Present the full search strategies for all databases, registers and websites, including any filters and limits used. | Page 6 |
| Selection process | 8 | Specify the methods used to decide whether a study met the inclusion criteria of the review, including how many reviewers screened each record and each report retrieved, whether they worked independently, and if applicable, details of automation tools used in the process. | Page 6 |
| Data collection process | 9 | Specify the methods used to collect data from reports, including how many reviewers collected data from each report, whether they worked independently, any processes for obtaining or confirming data from study investigators, and if applicable, details of automation tools used in the process. | Page 6 |
| Data items | 10a | List and define all outcomes for which data were sought. Specify whether all results that were compatible with each outcome domain in each study were sought (e.g. for all measures, time points, analyses), and if not, the methods used to decide which results to collect. | Page 6 |
|  | 10b | List and define all other variables for which data were sought (e.g. participant and intervention characteristics, funding sources). Describe any assumptions made about any missing or unclear information. | Page 6 |
| Study risk of bias assessment | 11 | Specify the methods used to assess risk of bias in the included studies, including details of the tool(s) used, how many reviewers assessed each study and whether they worked independently, and if applicable, details of automation tools used in the process. | Pages 6-7 |
| Effect measures | 12 | Specify for each outcome the effect measure(s) (e.g. risk ratio, mean difference) used in the synthesis or presentation of results. | Pages 7-8 |
| Synthesis methods | 13a | Describe the processes used to decide which studies were eligible for each synthesis (e.g. tabulating the study intervention characteristics and comparing against the planned groups for each synthesis (item #5)). | Pages 7-8 |
|  | 13b | Describe any methods required to prepare the data for presentation or synthesis, such as handling of missing summary statistics, or data conversions. | Pages 7-8 |
|  | 13c | Describe any methods used to tabulate or visually display results of individual studies and syntheses. | Pages 7-8 |
|  | 13d | Describe any methods used to synthesize results and provide a rationale for the choice(s). If meta-analysis was performed, describe the model(s), method(s) to identify the presence and extent of statistical heterogeneity, and software package(s) used. | Pages 7-8 |
|  | 13e | Describe any methods used to explore possible causes of heterogeneity among study results (e.g. subgroup analysis, meta-regression). | Pages 7-8 |
|  | 13f | Describe any sensitivity analyses conducted to assess robustness of the synthesized results. | Pages 7-8 |
| Reporting bias assessment | 14 | Describe any methods used to assess risk of bias due to missing results in a synthesis (arising from reporting biases). | Page 8 |
| Certainty assessment | 15 | Describe any methods used to assess certainty (or confidence) in the body of evidence for an outcome. | Pages 6-7 |
| **RESULTS** | | |  |
| Study selection | 16a | Describe the results of the search and selection process, from the number of records identified in the search to the number of studies included in the review, ideally using a flow diagram. | Page 8 |
|  | 16b | Cite studies that might appear to meet the inclusion criteria, but which were excluded, and explain why they were excluded. | Page 8 |
| Study characteristics | 17 | Cite each included study and present its characteristics. | Page 8 |
| Risk of bias in studies | 18 | Present assessments of risk of bias for each included study. | Pages 8-9 |
| Results of individual studies | 19 | For all outcomes, present, for each study: (a) summary statistics for each group (where appropriate) and (b) an effect estimate and its precision (e.g. confidence/credible interval), ideally using structured tables or plots. | Pages 9-11 |
| Results of syntheses | 20a | For each synthesis, briefly summarise the characteristics and risk of bias among contributing studies. | Pages 9-11 |
|  | 20b | Present results of all statistical syntheses conducted. If meta-analysis was done, present for each the summary estimate and its precision (e.g. confidence/credible interval) and measures of statistical heterogeneity. If comparing groups, describe the direction of the effect. | Pages 9-11 |
|  | 20c | Present results of all investigations of possible causes of heterogeneity among study results. | Pages 9-11 |
|  | 20d | Present results of all sensitivity analyses conducted to assess the robustness of the synthesized results. | Pages 9-11 |
| Reporting biases | 21 | Present assessments of risk of bias due to missing results (arising from reporting biases) for each synthesis assessed. | Page 12 |
| Certainty of evidence | 22 | Present assessments of certainty (or confidence) in the body of evidence for each outcome assessed. | Page 12 |
| **DISCUSSION** | | |  |
| Discussion | 23a | Provide a general interpretation of the results in the context of other evidence. | Pages 13-16 |
|  | 23b | Discuss any limitations of the evidence included in the review. | Pages 15-16 |
|  | 23c | Discuss any limitations of the review processes used. | Pages 15-16 |
|  | 23d | Discuss implications of the results for practice, policy, and future research. | Pages 13-16 |
| **OTHER INFORMATION** | | |  |
| Registration and protocol | 24a | Provide registration information for the review, including register name and registration number, or state that the review was not registered. | Page 4 |
|  | 24b | Indicate where the review protocol can be accessed, or state that a protocol was not prepared. | Page 4 |
|  | 24c | Describe and explain any amendments to information provided at registration or in the protocol. | NA |
| Support | 25 | Describe sources of financial or non-financial support for the review, and the role of the funders or sponsors in the review. | Page 21 |
| Competing interests | 26 | Declare any competing interests of review authors. | Page 21 |
| Availability of data, code and other materials | 27 | Report which of the following are publicly available and where they can be found: template data collection forms; data extracted from included studies; data used for all analyses; analytic code; any other materials used in the review. | Page 21 |

*From:*  Page MJ, McKenzie JE, Bossuyt PM, Boutron I, Hoffmann TC, Mulrow CD, et al. The PRISMA 2020 statement: an updated guideline for reporting systematic reviews. BMJ 2021;372:n71. doi: 10.1136/bmj.n71 For more information, visit: <http://www.prisma-statement.org/>

**Table S2: PRISMA checklist for abstract**

| **Section and Topic** | **Item #** | **Checklist item** | **Reported (Yes/No)** |
| --- | --- | --- | --- |
| **TITLE** | | |  |
| Title | 1 | Identify the report as a systematic review. | Yes |
| **BACKGROUND** | | |  |
| Objectives | 2 | Provide an explicit statement of the main objective(s) or question(s) the review addresses. | Yes |
| **METHODS** | | |  |
| Eligibility criteria | 3 | Specify the inclusion and exclusion criteria for the review. | Yes |
| Information sources | 4 | Specify the information sources (e.g. databases, registers) used to identify studies and the date when each was last searched. | Yes |
| Risk of bias | 5 | Specify the methods used to assess risk of bias in the included studies. | No |
| Synthesis of results | 6 | Specify the methods used to present and synthesise results. | Yes |
| **RESULTS** | | |  |
| Included studies | 7 | Give the total number of included studies and participants and summarise relevant characteristics of studies. | Yes |
| Synthesis of results | 8 | Present results for main outcomes, preferably indicating the number of included studies and participants for each. If meta-analysis was done, report the summary estimate and confidence/credible interval. If comparing groups, indicate the direction of the effect (i.e. which group is favoured). | Yes |
| **DISCUSSION** | | |  |
| Limitations of evidence | 9 | Provide a brief summary of the limitations of the evidence included in the review (e.g. study risk of bias, inconsistency and imprecision). | No |
| Interpretation | 10 | Provide a general interpretation of the results and important implications. | Yes |
| **OTHER** | | |  |
| Funding | 11 | Specify the primary source of funding for the review. | Yes in the text as per the author guidelines |
| Registration | 12 | Provide the register name and registration number. | Yes in the text as per the author guidelines |

*From:*  Page MJ, McKenzie JE, Bossuyt PM, Boutron I, Hoffmann TC, Mulrow CD, et al. The PRISMA 2020 statement: an updated guideline for reporting systematic reviews. BMJ 2021;372:n71. doi: 10.1136/bmj.n71

For more information, visit: <http://www.prisma-statement.org/>

# **Figure S1: PRISMA flow chart 2020**

**3405** Records identified from*:

PubMed (n= 2967)

Embase (n= 258)

WHO Global Literature on Coronavirus Disease (n= 180)

**2482** Records removed *before screening*:

Duplicate records removed (n = 165)

Records marked as ineligible by automation tools (n = 2317)

Records screened

(n = 923)

Records excluded**

(n = 649)

Reports sought for retrieval

(n = 274)

Reports not retrieved

(n = 1)

Reports assessed for eligibility

(n = 273 including 6 non-English reports)

Reports excluded (**n= 228**)

No PA prevalence (n =54)

Non-standard PA* definition (n = 44)

Mean Time (n= 29)

Wrong publication type (n = 25)

Difference (n=12)

Mean MET-min/day (n=12)

Wrong study design (n=11)

Mean step counts (n=10)

Duplicates (n=7)

Mean PA scores (n=6)

PA Mean weekly minutes (n=5)

PA not defined (n=4)

Data not retrievable (n=3)

Other language (n=2)

Wrong Outcome (n=2)

Numbers not reported (n=1)

Wrong population (n=1)

Records identified from:

Citation searching of 7 systematic reviews (n = 275)

Reports assessed for eligibility

(n = 275)

Reports excluded:

Already identified in the initial search (n = 176)

Irrelevant (n= 76)

Not meeting the eligibility criteria (n = 5)

Studies included in review

(n = 63)

**Identification of studies via databases and registers**

**Identification of studies via other methods**

**Identification**

**Screening**

**Included**

Reports sought for retrieval

(n = 275)

Reports not retrieved

(n = 0)

Reports eligible (n = 18)

PA- Physical activity

*From:*  Page MJ, McKenzie JE, Bossuyt PM, Boutron I, Hoffmann TC, Mulrow CD, et al. The PRISMA 2020 statement: an updated guideline for reporting systematic reviews. BMJ 2021;372:n71. doi: 10.1136/bmj.n71 For more information, visit: <http://www.prisma-statement.org/>

# **Box S1: Search strategy**

**Pubmed**:

Filter: January 1^st^, 2020- April 25, 2022

("Exercise"[MeSH Major Topic] OR ("sedentary behavior/epidemiology"[MeSH Major Topic] OR "sedentary behavior/statistics and numerical data"[MeSH Major Topic]) OR ("physical activity"[Text Word] OR "physical inactivity"[Text Word] OR "sedentary behavior"[Text Word])) AND ("severe acute respiratory syndrome coronavirus 2"[Supplementary Concept] OR "severe acute respiratory syndrome coronavirus 2"[All Fields] OR "2019 ncov"[All Fields] OR "2019nCoV"[All Fields] OR ("severe acute respiratory syndrome coronavirus 2"[Supplementary Concept] OR "severe acute respiratory syndrome coronavirus 2"[All Fields] OR "ncov"[All Fields] OR "2019 ncov"[All Fields] OR "covid 19"[All Fields] OR "sars cov 2"[All Fields] OR (("coronavirus"[All Fields] OR "cov"[All Fields]) AND 2019/11/01:3000/12/31[Date - Publication])) OR ("severe acute respiratory syndrome coronavirus 2"[Supplementary Concept] OR "severe acute respiratory syndrome coronavirus 2"[All Fields] OR "sars cov 2"[All Fields]) OR ("wuhan"[All Fields] AND ("coronavirus"[MeSH Terms] OR "coronavirus"[All Fields] OR "coronaviruses"[All Fields])) OR ("2019"[All Fields] AND ("novel"[All Fields] OR "novel s"[All Fields] OR "novels"[All Fields]) AND ("coronavirus"[MeSH Terms] OR "coronavirus"[All Fields] OR "coronaviruses"[All Fields])))

**Embase**:

Filter: human and exclude medline journals and yr="2020" and covid-19, January 1^st^ -November 18, 2020

(physical inactivity.mp. or exp exercise/ or exp physical inactivity/ or exp physical activity/) OR (physical activity.mp.)

**WHO Global literature on coronavirus disease**: January 1^st^-November 18, 2020

<https://www.who.int/emergencies/diseases/novel-coronavirus-2019/global-research-on-novel-coronavirus-2019-ncov>

Filter: 2020, Risk factors (157), Observational study (51), Prevalence study (46), Incidence study (14),

(tw:(Physical activity)) OR (tw:(inactivity))

- Type of study
  - Risk factors [(remover)](javascript:remove_filter('type_of_study_1'))
  - Prevalence study [(remover)](javascript:remove_filter('type_of_study_2'))
  - Observational study [(remover)](javascript:remove_filter('type_of_study_3'))
  - Controlled clinical trial [(remover)](javascript:remove_filter('type_of_study_4'))
  - Incidence study [(remover)](javascript:remove_filter('type_of_study_5'))
- Year
  - 2020 [(remover)](javascript:remove_filter('year_cluster_1'))

# **Table S3: Quality assessment (QA) checklist for the original studies**

| **QA domain name** | **QA domain classification** | **Definition** |
| --- | --- | --- |
| Population characteristics | **High ROB:** Not defined | Missing all characteristics listed below |
|  | **Low ROB:** Clearly defined | The following characteristics were specified:   1. Gender 2. Population type (general or clinical population) 3. Adults/youth/ age group/adolescents |
| Outcome definition | **Low ROB:** Standard definition (Clearly stated definition of PA/ inactivity consistent with international recommendations for the general population from the WHO and similar organizations with published physical activity guidelines) (1-3).  General population exclude pregnant and lactating women | Physical activity  Infants aged 3-5:  3 hours per day of high-impact, dynamic, short duration exercise  Youth aged 5–17:  = or > 60 minutes of moderate to vigorous intensity physical activity per day  Adults aged 18-64:  = or >150 minutes of moderate-intensity aerobic PA/week  **OR**  = or >75 minutes of vigorous-intensity aerobic physical activity/week  **OR**  An equivalent combination of moderate and vigorous-intensity activity  **OR**  = or > 30 minutes of moderate to vigorous activity, 3 or more days/week  **OR**  > 30 minutes of moderate-intensity physical activity on most days of the week  **OR**  500 to 1,000 MET-minutes per week of aerobic physical activity  **OR**  3 or more days of vigorous-intensity activity of at least 20 minutes per day  **OR**  5 or more days of moderate-intensity activity and/or walking of at least 30 minutes per day  **OR**  5 or more days of any combination of walking, moderate-intensity or vigorous intensity activities achieving a minimum  **OR**  Total physical activity of at least 600 MET-minutes/week.  Physical inactivity:  An absence of physical activity or exercise  **OR**  Insufficient amounts of PA (under the recommended levels (minutes or hours/d, minutes or hours/week, METS, PAL))  **OR**  Not achieving 180 minutes of PA of any intensity per day among 1-4 years |
| Measurement tool | **High ROB** Not defined | The used instrument was not defined |
|  | **High ROB :** A non-validated questionnaire (subjective) | Use of a non-validated and non-standard questionnaire |
|  | **Low ROB:** Validated questionnaire (subjective) | Use of a validated standard physical activity or inactivity/sedentary behavior instrument |
|  | **Low ROB:** A gold standard (objective) | Used an objective instrument, such as, accelerometer (gold standard) or pedometer |
| Setting | **High ROB:** Not defined/ not clearly defined | No definition |
|  | **Low ROB:** Setting clearly defined | Online survey or community, clinical |
| Timing | **High ROB:** Not defined | Data collection time not stated |
|  | **Moderate ROB:** Partly defined | Defined data collection time |
|  | **Moderate ROB:** Partly defined | Data collection time not stated but specified information on when it was done, e.g: before the pandemic or during the pandemic with details of restrictions |
|  | **Low ROB:** Clearly defined | Defined data collection time and the time when it was done, e.g: during the pandemic, before the pandemic with the details of restrictions |
| Sampling method | **High ROB:** Not defined | Not stated |
|  | **High ROB:** Unclear | Self-selection or unclear method |
|  | **Moderate ROB:** Non-random sampling | Clearly defined non-random sampling method (e.g. Convenient) |
|  | **Low ROB:** Random sampling | Clearly defined random sampling |
| Response rate  For each reported data point  if only global response rate is reported, the response rate per strata (male/female) would be missing | **High ROB:** Not defined | The response rate was not reported for the study population for which the prevalence was reported  e.g. the males and female’s response rate are different than the response rate for the whole study population. |
|  | **High ROB** <49% | Low response rate |
|  | **Moderate ROB:** 50-79% | Acceptable response rate in this context |
|  | **Low ROB:** >=80% | Good response rate |

Abbreviations:

QA: Quality assessment

MET Metabolic Equivalent of task

PA Physical activity

d day

ROB Risk of Bias

# Box S2: List of excluded studies

1. Abbas AM, Fathy SK, Fawzy AT, Salem AS, Shawky MS. The mutual effects of COVID-19 and obesity. Obesity Medicine. 2020;19(100250).

2. Abbas AM, Kamel MM. Dietary habits in adults during quarantine in the context of COVID-19 pandemic. Obesity Medicine. 2020;19(100254).

3. Ács P, Prémusz V, Morvay-Sey K, Pálvölgyi Á, Trpkovici M, Elbert G, et al. Effects Of COVID-19 on physical actvitiy behavior among university students: Results of a Hungarian online survey Health Problems of Civilization. 2020;14(3):174-82.

4. Ainsworth BE, Li F. Physical activity during the coronavirus disease-2019 global pandemic. Journal of Sport and Health Science. 2020;9(4):291-2.

5. Al Fagih A, Al Onazi M, Al Basiri S, Al-Kaf F, Dagriri K, Al Hebaishi Y, et al. Remotely monitored inactivity due to COVID-19 lockdowns. Potential hazard for heart failure patients. Saudi Med J. 2020;41(11):1211-6.

6. Al-Musharaf S. Prevalence and Predictors of Emotional Eating among Healthy Young Saudi Women during the COVID-19 Pandemic. Nutrients. 2020;12(10).

7. Almandoz JP, Xie L, Schellinger JN, Mathew MS, Gazda C, Ofori A, et al. Impact of COVID-19 stay-at-home orders on weight-related behaviours among patients with obesity. Clin Obes. 2020;10(5):e12386.

8. Alomari MA, Khabour OF, Alzoubi KH. Changes in physical activity and sedentary behavior amid confinement: The bksq-covid-19 project. Risk Management and Healthcare Policy. 2020;13:1757-64.

9. Alves JM, Yunker AG, DeFendis A, Xiang AH, Page KA. Prenatal exposure to gestational diabetes is associated with anxiety and physical inactivity in children during COVID-19. Clin Obes. 2020:e12422-e.

10. Amatriain-Fernandez S, Gronwald T, Murillo-Rodriguez E, Imperatori C, Solano AF, Latini A, et al. Physical Exercise Potentials Against Viral Diseases Like COVID-19 in the Elderly. Frontiers in Medicine. 2020;7(379).

11. Ammar A, Brach M, Trabelsi K, Chtourou H, Boukhris O, Masmoudi L, et al. Effects of COVID-19 Home Confinement on Eating Behaviour and Physical Activity: Results of the ECLB-COVID19 International Online Survey. Nutrients. 2020;12(6).

12. Angelini S, Pinto A, Hrelia P, Malaguti M, Buccolini F, Donini LM, et al. The "Elderly" Lesson in a "Stressful" Life: Italian Holistic Approach to Increase COVID-19 Prevention and Awareness. Front Endocrinol (Lausanne). 2020;11:579401.

13. Angosto S, Berengüí R, Vegara-Ferri JM, López-Gullón JM. Motives and Commitment to Sport in Amateurs during Confinement: A Segmentation Study. Int J Environ Res Public Health. 2020;17(20).

14. Antilao PL. [Physical activity and Coronavirus outbreak. What measures will be adopted for the elderly in Chile?]. Rev Med Chil. 2020;148(2):271-2.

15. Arcellana AE, Jimeno C. Challenges and opportunities for diabetes care in the philippines in the time of the covid-19 pandemic. Journal of the ASEAN Federation of Endocrine Societies. 2020;35(1):55-7.

16. Asiamah N, Opuni FF, Mends-Brew E, Mensah SW, Mensah HK, Quansah F. Short-Term Changes in Behaviors Resulting from COVID-19-Related Social Isolation and Their Influences on Mental Health in Ghana. Community Ment Health J. 2020:1-14.

17. Assaloni R, Pellino VC, Puci MV, Ferraro OE, Lovecchio N, Girelli A, et al. Coronavirus disease (Covid-19): How does the exercise practice in active people with type 1 diabetes change? A preliminary survey. Diabetes Res Clin Pract. 2020;166:108297-.

18. Aubertin-Leheudre M, Rolland Y. The Importance of Physical Activity to Care for Frail Older Adults During the COVID-19 Pandemic. J Am Med Dir Assoc. 2020;21(7):973-6.

19. Baceviciene M, Jankauskiene R. Changes in sociocultural attitudes towards appearance, body image, eating attitudes and behaviours, physical activity, and quality of life in students before and during COVID-19 lockdown. Appetite. 2021;166:105452.

20. Balanzá-Martínez V, Kapczinski F, de Azevedo Cardoso T, Atienza-Carbonell B, Rosa AR, Mota JC, et al. The assessment of lifestyle changes during the COVID-19 pandemic using a multidimensional scale. Rev psiquiatr salud ment (Barc, Ed impr). 2020.

21. Balducci S, Coccia EM. Sedentariness and physical activity in type 2 diabetes during the COVID-19 pandemic. Diabetes Metab Res Rev. 2020:e3378.

22. Barbu MG, Thompson DC, Popescu MN, Beiu C, Mihai MM, Enachescu CI. A Romanian survey on the impact of sars-cov-2 pandemic on dystonia patients. Romanian Journal of Legal Medicine. 2020;28(2):208-11.

23. Barchetta I, Cimini FA, Bertoccini L, Ceccarelli V, Spaccarotella M, Baroni MG, et al. Effects of work status changes and perceived stress onglycaemiccontrol in individuals with type 1 diabetes during COVID-19 lockdown in Italy. Diabetes Res Clin Pract. 2020;170:108513.

24. Barkley JE, Lepp A, Glickman E, Farnell G, Beiting J, Wiet R, et al. The Acute Effects of the COVID-19 Pandemic on Physical Activity and Sedentary Behavior in University Students and Employees. Int J Exerc Sci. 2020;13(5):1326-39.

25. Barone MTU, Harnik SB, de Luca PV, Lima BLS, Wieselberg RJP, Ngongo B, et al. The impact of COVID-19 on people with diabetes in Brazil. Diabetes Res Clin Pract. 2020;166:108304.

26. Bartlett L, Brady JJR, Farrow M, Kim S, Bindoff A, Fair H, et al. Change in modifiable dementia risk factors during COVID-19 lockdown: The experience of over 50s in Tasmania, Australia. Alzheimers Dement (N Y). 2021;7(1):e12169.

27. Barwais FA. Assessing physical activity and sedentary time during the COVID-19 pandemic using self-reported measurement. National Journal of Physiology, Pharmacy and Pharmacology. 2020;10(11):1019-24.

28. Bezerra ACV, Silva C, Soares FRG, Silva J. Factors associated with people's behavior in social isolation during the COVID-19 pandemic. Cien Saude Colet. 2020;25:2411-21.

29. Biviá-Roig G, La Rosa VL, Gómez-Tébar M, Serrano-Raya L, Amer-Cuenca JJ, Caruso S, et al. Analysis of the Impact of the Confinement Resulting from COVID-19 on the Lifestyle and Psychological Wellbeing of Spanish Pregnant Women: An Internet-Based Cross-Sectional Survey. Int j environ res public health (Online). 2020;17(16).

30. Bland KA, Bigaran A, Campbell KL, Trevaskis M, Zopf EM. Exercising in Isolation? The Role of Telehealth in Exercise Oncology During the COVID-19 Pandemic and Beyond. Phys Ther. 2020;100(10):1713-6.

31. Blom V, Lönn A, Ekblom B, Kallings LV, Väisänen D, Hemmingsson E, et al. Lifestyle habits and mental health in light of the two COVID-19 pandemic waves in Sweden, 2020. International Journal of Environmental Research and Public Health. 2021;18(6):3313.

32. Borges-Machado F, Barros D, Ribeiro Ó, Carvalho J. The effects of COVID-19 home confinement in dementia care: physical and cognitive decline, severe neuropsychiatric symptoms and increased caregiving burden. American Journal of Alzheimer's Disease & Other Dementias®. 2020;35:1533317520976720.

33. Bourassa KJ, Sbarra DA, Caspi A, Moffitt TE. Social Distancing as a Health Behavior: County-Level Movement in the United States During the COVID-19 Pandemic Is Associated with Conventional Health Behaviors. Ann Behav Med. 2020;54(8):548-56.

34. Bourdas DI, Zacharakis ED. Evolution of changes in physical activity over lockdown time: Physical activity datasets of four independent adult sample groups corresponding to each of the last four of the six COVID-19 lockdown weeks in Greece. Data Brief. 2020;32:106301.

35. Bowes A, Lomax L, Piasecki J. The impact of the COVID-19 lockdown on elite sportswomen. Managing Sport and Leisure. 2020:1-17.

36. Branley-Bell D, Talbot CV. Exploring the impact of the COVID-19 pandemic and UK lockdown on individuals with experience of eating disorders. J Eat Disord. 2020;8:44.

37. Brooks JHM, Tingay R, Varney J. Social distancing and COVID-19: an unprecedented active transport public health opportunity. Br J Sports Med. 2020.

38. Brown EG, Chahine LM, Goldman SM, Korell M, Mann E, Kinel DR, et al. The effect of the COVID-19 pandemic on people with Parkinson’s disease. Journal of Parkinson's disease. 2020;10(4):1365-77.

39. Brown L, Mossabir R, Harrison N, Brundle C, Smith J, Clegg A. Life in lockdown: A telephone survey to investigate the impact of COVID-19 lockdown measures on the lives of older people (≥ 75 years). Age Ageing. 2020.

40. Browne RAV, Macêdo GAD, Cabral LLP, Oliveira GTA, Vivas A, Fontes EB, et al. Initial impact of the COVID-19 pandemic on physical activity and sedentary behavior in hypertensive older adults: An accelerometer-based analysis. Exp Gerontol. 2020;142:111121.

41. Buoite Stella A, Ajčević M, Furlanis G, Cillotto T, Menichelli A, Accardo A, et al. Smart technology for physical activity and health assessment during COVID-19 lockdown. J Sports Med Phys Fitness. 2020.

42. Burtscher J, Burtscher M, Millet GP. (Indoor) isolation, stress, and physical inactivity: Vicious circles accelerated by COVID-19? Scand J Med Sci Sports. 2020;30(8):1544-5.

43. Calcaterra V, Vandoni M, Pellino VC, Cena H. Special Attention to Diet and Physical Activity in Children and Adolescents With Obesity During the Coronavirus Disease-2019 Pandemic. Front Pediatr. 2020;8:407.

44. Callow DD, Arnold-Nedimala NA, Jordan LS, Pena GS, Won J, Woodard JL, et al. The Mental Health Benefits of Physical Activity in Older Adults Survive the COVID-19 Pandemic. Am J Geriatr Psychiatry. 2020;28(10):1046-57.

45. Campennì P, Marra AA, Ferri L, Orefice R, Parello A, Litta F, et al. Impact of COVID-19 Quarantine on Advanced Hemorrhoidal Disease and the Role of Telemedicine in Patient Management. J Clin Med. 2020;9(11).

46. Cancello R, Soranna D, Zambra G, Zambon A, Invitti C. Determinants of the Lifestyle Changes during COVID-19 Pandemic in the Residents of Northern Italy. Int J Environ Res Public Health. 2020;17(17).

47. Carriedo A, Cecchini JA, Fernández-Río J, Méndez-Giménez A. Resilience and physical activity in people under home isolation due to COVID-19: A preliminary evaluation. Ment Health Phys Act. 2020;19:100361.

48. Carroll N, Sadowski A, Laila A, Hruska V, Nixon M, Ma DWL, et al. The Impact of COVID-19 on Health Behavior, Stress, Financial and Food Security among Middle to High Income Canadian Families with Young Children. Nutrients. 2020;12(8).

49. Caruso I, Di Molfetta S, Guarini F, Giordano F, Cignarelli A, Natalicchio A, et al. Reduction of hypoglycaemia, lifestyle modifications and psychological distress during lockdown following SARS-CoV-2 outbreak in type 1 diabetes. Diabetes Metab Res Rev. 2020:e3404.

50. Carvalho VO, Gois CO. COVID-19 pandemic and home-based physical activity. J Allergy Clin Immunol Pract. 2020;8(8):2833-4.

51. Castañeda-Babarro A, Arbillaga-Etxarri A, Gutiérrez-Santamaría B, Coca A. Physical Activity Change during COVID-19 Confinement. Int J Environ Res Public Health. 2020;17(18).

52. Castellini G, Cassioli E, Rossi E, Innocenti M, Gironi V, Sanfilippo G, et al. The impact of COVID-19 epidemic on eating disorders: A longitudinal observation of pre versus post psychopathological features in a sample of patients with eating disorders and a group of healthy controls. International Journal of Eating Disorders. 2020;53(11):1855-62.

53. Cavallieri F, Sireci F, Fioravanti V, Toschi G, Rispoli V, Antonelli F, et al. Parkinson's disease patients' needs during the COVID‐19 pandemic in a red zone: A framework analysis of open‐ended survey questions. European journal of neurology. 2021;28(10):3254-62.

54. Chagué F, Boulin M, Eicher JC, Bichat F, Saint Jalmes M, Cransac-Miet A, et al. Impact of lockdown on patients with congestive heart failure during the coronavirus disease 2019 pandemic. ESC Heart Fail. 2020.

55. Chang YK, Hung CL, Timme S, Nosrat S, Chu CH. Exercise Behavior and Mood during the COVID-19 Pandemic in Taiwan: Lessons for the Future. Int J Environ Res Public Health. 2020;17(19).

56. Cheikh Ismail L, Osaili TM, Mohamad MN, Al Marzouqi A, Jarrar AH, Abu Jamous DO, et al. Eating Habits and Lifestyle during COVID-19 Lockdown in the United Arab Emirates: A Cross-Sectional Study. Nutrients. 2020;12(11).

57. Cheikh Ismail L, Osaili TM, Mohamad MN, Al Marzouqi A, Jarrar AH, Zampelas A, et al. Assessment of Eating Habits and Lifestyle during Coronavirus Pandemic in the MENA region: A Cross-Sectional Study. Br J Nutr. 2020:1-30.

58. Chen P, Mao L, Nassis GP, Harmer P, Ainsworth BE, Li F. Coronavirus disease (COVID-19): The need to maintain regular physical activity while taking precautions. Journal of Sport and Health Science. 2020;9(2):103-4.

59. Chen Y, Osika W, Henriksson G, Dahlstrand J, Friberg P. Impact of COVID-19 pandemic on mental health and health behaviors in Swedish adolescents. Scand J Public Health. 2022;50(1):26-32.

60. Cheval B, Sivaramakrishnan H, Maltagliati S, Fessler L, Forestier C, Sarrazin P, et al. Relationships between changes in self-reported physical activity, sedentary behaviour and health during the coronavirus (COVID-19) pandemic in France and Switzerland. J Sports Sci. 2020:1-6.

61. Chirico A, Lucidi F, Galli F, Giancamilli F, Vitale J, Borghi S, et al. COVID-19 Outbreak and Physical Activity in the Italian Population: A Cross-Sectional Analysis of the Underlying Psychosocial Mechanisms. Front Psychol. 2020;11:2100.

62. Chouchou F, Augustini M, Caderby T, Caron N, Turpin NA, Dalleau G. The importance of sleep and physical activity on well-being during COVID-19 lockdown: reunion island as a case study. Sleep Med. 2020.

63. Colley RC, Bushnik T, Langlois K. Exercise and screen time during the COVID-19 pandemic. Health Rep. 2020;31(6):3-11.

64. Constandt B, Thibaut E, De Bosscher V, Scheerder J, Ricour M, Willem A. Exercising in Times of Lockdown: An Analysis of the Impact of COVID-19 on Levels and Patterns of Exercise among Adults in Belgium. Int j environ res public health (Online). 2020;17(11).

65. Constant A, Conserve DF, Gallopel-Morvan K, Raude J. Socio-Cognitive Factors Associated With Lifestyle Changes in Response to the COVID-19 Epidemic in the General Population: Results From a Cross-Sectional Study in France. Front Psychol. 2020;11:579460.

66. Cooper JA, vanDellen M, Bhutani S. Self-weighing Practices and Associated Health Behaviors during COVID-19. American Journal of Health Behavior. 2021;45(1):17-30.

67. Coughenour C, Gakh M, Pharr JR, Bungum T, Jalene S. Changes in Depression and Physical Activity Among College Students on a Diverse Campus After a COVID-19 Stay-at-Home Order. J Community Health. 2020:1-9.

68. Coyle C, Ghazi H, Georgiou I. The mental health and well-being benefits of exercise during the COVID-19 pandemic: a cross-sectional study of medical students and newly qualified doctors in the UK. Ir J Med Sci. 2020:1-2.

69. Cransac-Miet A, Zeller M, Chagué F, Faure AS, Bichat F, Danchin N, et al. Impact of COVID-19 lockdown on lifestyle adherence in stay-at-home patients with chronic coronary syndromes: Towards a time bomb. Int J Cardiol. 2020.

70. Crisafulli A, Pagliaro P. Physical activity/inactivity and COVID-19. Eur J Prev Cardiol. 2020:2047487320927597.

71. Curtis RG, Olds T, Ferguson T, Fraysse F, Dumuid D, Esterman A, et al. Changes in diet, activity, weight, and wellbeing of parents during COVID-19 lockdown. PLoS One. 2021;16(3):e0248008.

72. de Luis Román DA, Izaola O, Primo Martín D, Gómez Hoyos E, Torres Torres B, López Gómez JJ. Effect of lockdown for COVID-19 on self-reported body weight gain in a sample of obese patients. Nutr Hosp. 2020.

73. de Souza FR, Motta-Santos D, Dos Santos Soares D, de Lima JB, Cardozo GG, Guimarães LSP, et al. Association of physical activity levels and the prevalence of COVID-19-associated hospitalization. J Sci Med Sport. 2021;24(9):913-8.

74. Delisle Nyström C, Alexandrou C, Henström M, Nilsson E, Okely AD, Wehbe El Masri S, et al. International Study of Movement Behaviors in the Early Years (SUNRISE): Results from SUNRISE Sweden's Pilot and COVID-19 Study. Int J Environ Res Public Health. 2020;17(22).

75. Deng C-H, Wang J-Q, Zhu L-M, Liu H-W, Guo Y, Peng X-H, et al. Association of Web-Based Physical Education With Mental Health of College Students in Wuhan During the COVID-19 Outbreak: Cross-Sectional Survey Study. J Med Internet Res. 2020;22(10):e21301-e.

76. Deschasaux-Tanguy M, Druesne-Pecollo N, Esseddik Y, de Edelenyi FS, Allès B, Andreeva VA, et al. Diet and physical activity during the coronavirus disease 2019 (COVID-19) lockdown (March-May 2020): results from the French NutriNet-Santé cohort study. Am J Clin Nutr. 2021;113(4):924-38.

77. Dhivyadharshini J, Somasundaram J. Impact of physical inactivity and psychological interventions during the pandemic COVID 19 among chennai population. International Journal of Pharmaceutical Research. 2020;12:596-605.

78. Di Corrado D, Magnano P, Muzii B, Coco M, Guarnera M, De Lucia S, et al. Effects of social distancing on psychological state and physical activity routines during the COVID-19 pandemic. Sport Sci Health. 2020:1-6.

79. Di Dalmazi G, Maltoni G, Bongiorno C, Tucci L, Di Natale V, Moscatiello S, et al. Comparison of the effects of lockdown due to COVID-19 on glucose patterns among children, adolescents, and adults with type 1 diabetes: CGM study. BMJ Open Diabetes Res Care. 2020;8(2).

80. Di Renzo L, Gualtieri P, Pivari F, Soldati L, Attinà A, Cinelli G, et al. Eating habits and lifestyle changes during COVID-19 lockdown: an Italian survey. J Transl Med. 2020;18(1):229.

81. Di Santo SG, Franchini F, Filiputti B, Martone A, Sannino S. The Effects of COVID-19 and Quarantine Measures on the Lifestyles and Mental Health of People Over 60 at Increased Risk of Dementia. Frontiers in Psychiatry. 2020;11(578628).

82. Di Stefano V, Battaglia G, Giustino V, Gagliardo A, Aleo M, Giannini O, et al. Significant reduction of physical activity in patients with neuromuscular disease during COVID-19 pandemic: the long-term consequences of quarantine. J neurol. 2020.

83. Di Stefano V, Ornello R, Gagliardo A, Torrente A, Illuminato E, Caponnetto V, et al. Social Distancing in Chronic Migraine during the COVID-19 Outbreak: Results from a Multicenter Observational Study. Nutrients. 2021;13(4).

84. Ding D, Cheng M, Del Pozo Cruz B, Lin T, Sun S, Zhang L, et al. How COVID-19 lockdown and reopening affected daily steps: evidence based on 164,630 person-days of prospectively collected data from Shanghai, China. Int J Behav Nutr Phys Act. 2021;18(1):40.

85. Ding D, Del Pozo Cruz B, Green MA, Bauman AE. Is the COVID-19 lockdown nudging people to be more active: a big data analysis. Br J Sports Med. 2020;54(20):1183-4.

86. Do BN, Tran TV, Phan DT, Nguyen HC, Nguyen TTP, Nguyen HC, et al. Health Literacy, eHealth Literacy, Adherence to Infection Prevention and Control Procedures, Lifestyle Changes, and Suspected COVID-19 Symptoms Among Health Care Workers During Lockdown: Online Survey. J Med Internet Res. 2020;22(11):e22894.

87. Dogas Z, Lusic Kalcina L, Pavlinac Dodig I, Demirovic S, Madirazza K, Valic M, et al. The effect of COVID-19 lockdown on lifestyle and mood in Croatian general population: a cross-sectional study. Croat Med J. 2020;61(4):309-18.

88. Duncan GE, Avery AR, Seto E, Tsang S. Perceived change in physical activity levels and mental health during COVID-19: Findings among adult twin pairs. PLoS One. 2020;15(8):e0237695-e.

89. Dunton GF, Do B, Wang SD. Early effects of the COVID-19 pandemic on physical activity and sedentary behavior in children living in the U.S. BMC Public Health. 2020;20(1):1351.

90. Dunton GF, Wang SD, Do B, Courtney J. Early Effects of the COVID-19 Pandemic on Physical Activity Locations and Behaviors in Adults Living in the United States. Prev Med Rep. 2020:101241.

91. Elnaggar RK, Alqahtani BA, Mahmoud WS, Elfakharany MS. Physical Activity in Adolescents During the Social Distancing Policies of the COVID-19 Pandemic. Asia Pac J Public Health. 2020:1010539520963564.

92. Elran-Barak R, Mozeikov M. One Month into the Reinforcement of Social Distancing due to the COVID-19 Outbreak: Subjective Health, Health Behaviors, and Loneliness among People with Chronic Medical Conditions. Int j environ res public health (Online). 2020;17(15).

93. Elsalem L, Al-Azzam N, Jum'ah AA, Obeidat N, Sindiani AM, Kheirallah KA. Stress and behavioral changes with remote E-exams during the Covid-19 pandemic: A cross-sectional study among undergraduates of medical sciences. Annals of Medicine and Surgery. 2020;60:271-9.

94. Emerson KG. Coping with being cooped up: Social distancing during COVID-19 among 60+ in the United States. Rev Panam Salud Publica. 2020;44:e81.

95. Endstrasser F, Braito M, Linser M, Spicher A, Wagner M, Brunner A. The negative impact of the COVID-19 lockdown on pain and physical function in patients with end-stage hip or knee osteoarthritis. Knee Surg Sports Traumatol Arthrosc. 2020;28(8):2435-43.

96. Ernstsen L, Havnen A. Mental health and sleep disturbances in physically active adults during the COVID-19 lockdown in Norway: does change in physical activity level matter? Sleep medicine. 2020.

97. Esain I, Gil MS, Dunabeitia I, Rodriguez-Larrad A, Bidaurrazaga-Letona I. Effects of COVID-19 Lockdown on Physical Activity and Health-Related Quality of Life in Older Adults Who Regularly Exercise. Sustainability. 2021;7(13):3771.

98. FA B. Barwais FA.Physical activity at home during the COVID-19 pandemic in the two Most-affected cities in Saudi Arabia. Open Public Health J. 2020;13:470–6.

99. Fernandez-Rio J, Cecchini JA, Mendez-Gimenez A, Carriedo A. Weight changes during the COVID-19 home confinement. Effects on psychosocial variables. Obes Res Clin Pract. 2020;14(4):383-5.

100. Fillon A, Genin P, Larras B, Vanhelst J, Luiggi M, Aubert S, et al. France's 2020 Report Card on Physical Activity and Sedentary Behaviors in Children and Youth: Results and Progression. J Phys Act Health. 2021;18(7):811-7.

101. Flanagan EW, Beyl RA, Fearnbach SN, Altazan AD, Martin CK, Redman LM. The impact of COVID-19 stay-at-home orders on health behaviors in adults. Obesity (Silver Spring). 2020.

102. Folk AL, Wagner BE, Hahn SL, Larson N, Barr-Anderson DJ, Neumark-Sztainer D. Changes to Physical Activity during a Global Pandemic: A Mixed Methods Analysis among a Diverse Population-Based Sample of Emerging Adults in the U.S. Int J Environ Res Public Health. 2021;18(7).

103. Gallè F, Sabella EA, Da Molin G, De Giglio O, Caggiano G, Di Onofrio V, et al. Understanding Knowledge and Behaviors Related to CoViD-19 Epidemic in Italian Undergraduate Students: The EPICO Study. Int j environ res public health (Online). 2020;17(10).

104. Gallego-Gómez JI, Campillo-Cano M, Carrión-Martínez A, Balanza S, Rodríguez-González-Moro MT, Simonelli-Muñoz AJ, et al. The COVID-19 Pandemic and Its Impact on Homebound Nursing Students. Int J Environ Res Public Health. 2020;17(20).

105. Gallo LA, Gallo TF, Young SL, Moritz KM, Akison LK. The Impact of Isolation Measures Due to COVID-19 on Energy Intake and Physical Activity Levels in Australian University Students. Nutrients. 2020;12(6).

106. Gao Z, Lee JE, McDonough DJ, Albers C. Virtual reality exercise as a coping strategy for health and wellness promotion in older adults during the COVID-19 pandemic. Journal of Clinical Medicine. 2020;9(1986).

107. García-Fernández L, Romero-Ferreiro V, López-Roldán PD, Padilla S, Rodriguez-Jimenez R. Mental Health in Elderly Spanish People in Times of COVID-19 Outbreak. Am J Geriatr Psychiatry. 2020;28(10):1040-5.

108. García-Tascón M, Sahelices-Pinto C, Mendaña-Cuervo C, Magaz-González AM. The Impact of the COVID-19 Confinement on the Habits of PA Practice According to Gender (Male/Female): Spanish Case. Int J Environ Res Public Health. 2020;17(19).

109. Ghosh A, Arora B, Gupta R, Anoop S, Misra A. Effects of nationwide lockdown during COVID-19 epidemic on lifestyle and other medical issues of patients with type 2 diabetes in north India. Diabetes Metab Syndr. 2020;14(5):917-20.

110. Gilic B, Ostojic L, Corluka M, Volaric T, Sekulic D. Contextualizing Parental/Familial Influence on Physical Activity in Adolescents before and during COVID-19 Pandemic: A Prospective Analysis. Children (Basel). 2020;7(9).

111. Gilic B, Zenic N, Separovic V, Jurcev Savicevic A, Sekulic D. Evidencing the influence of pre-pandemic sports participation and substance misuse on physical activity during the COVID‑19 lockdown: a prospective analysis among older adolescents. Int J Occup Med Environ Health. 2021;34(2):151-63.

112. Giuntella O, Hyde K, Saccardo S, Sadoff S. Lifestyle and mental health disruptions during COVID-19. Proc Natl Acad Sci U S A. 2021;118(9).

113. Goethals L, Barth N, Guyot J, Hupin D, Celarier T, Bongue B. Impact of Home Quarantine on Physical Activity Among Older Adults Living at Home During the COVID-19 Pandemic: Qualitative Interview Study. JMIR Aging. 2020;3(1):e19007.

114. Gomes CM, Favorito LA, Henriques JVT, Canalini AF, Anzolch KMJ, de Carvalho Fernandes R, et al. Impact of COVID-19 on clinical practice, income, health and lifestyle behavior of Brazilian urologists. Int Braz J Urol. 2020;46(6):1042-71.

115. Górnicka M, Drywien ME, Zielinska MA, Hamulka J. Dietary and Lifestyle Changes During COVID-19 and the Subsequent Lockdowns among Polish Adults: A Cross-Sectional Online Survey PLifeCOVID-19 Study. Nutrients. 2020;12(8).

116. Grabia M, Markiewicz-Żukowska R, Puścion-Jakubik A, Bielecka J, Nowakowski P, Gromkowska-Kępka K, et al. The Nutritional and Health Effects of the COVID-19 Pandemic on Patients with Diabetes Mellitus. Nutrients. 2020;12(10).

117. Gupta R, Grover S, Basu A, Krishnan V, Tripathi A, Subramanyam A, et al. Changes in sleep pattern and sleep quality during COVID-19 lockdown. Indian Journal of Psychiatry. 2020;62(4):370-8.

118. Gupta S, Tang C, Higgs P. Social isolation during Covid-19: Boon or bane to diabetes management. Diabetes Metab Syndr. 2020;14(4):567-8.

119. Haddad C, Zakhour M, Bou Kheir M, Haddad R, Al Hachach M, Sacre H, et al. Association between eating behavior and quarantine/confinement stressors during the coronavirus disease 2019 outbreak. J Eat Disord. 2020;8:40.

120. Hadianfard AM, Mozaffari-Khosravi H, Karandish M, Azhdari M. Physical activity and sedentary behaviors (screen time and homework) among overweight or obese adolescents: a cross-sectional observational study in Yazd, Iran. BMC Pediatr. 2021;21(1):421.

121. Hamer M, Kivimäki M, Gale CR, Batty GD. Lifestyle risk factors, inflammatory mechanisms, and COVID-19 hospitalization: A community-based cohort study of 387,109 adults in UK. Brain Behav Immun. 2020;87:184-7.

122. He M, Xian Y, Lv X, He J, Ren Y. Changes in Body Weight, Physical Activity, and Lifestyle During the Semi-lockdown Period After the Outbreak of COVID-19 in China: An Online Survey. Disaster Med Public Health Prep. 2020:1-6.

123. Helm EE, Kempski KA, Galantino MLA. Effect of disrupted rehabilitation services on distress and quality of life in breast cancer survivors during the COVID-19 pandemic. Rehabilitation Oncology. 2020;38(4):153-8.

124. Hemphill NM, Kuan MTY, Harris KC. Reduced Physical Activity During COVID-19 Pandemic in Children With Congenital Heart Disease. Can J Cardiol. 2020;36(7):1130-4.

125. Hino K, Asami Y. Change in walking steps and association with built environments during the COVID-19 state of emergency: A longitudinal comparison with the first half of 2019 in Yokohama, Japan. Health Place. 2021;69:102544.

126. Hirase T, Okita M, Nakai Y, Akaida S, Shono S, Makizako H. Pain and physical activity changes during the COVID-19 state of emergency among Japanese adults aged 40 years or older: A cross-sectional study. Medicine (Baltimore). 2021;100(41):e27533.

127. Huber BC, Steffen J, Schlichtiger J, Graupe T, Deuster E, Strouvelle VP, et al. Alteration of physical activity during COVID-19 pandemic lockdown in young adults. J Transl Med. 2020;18(1):410.

128. Huckins JF, daSilva AW, Wang W, Hedlund E, Rogers C, Nepal SK, et al. Mental Health and Behavior of College Students During the Early Phases of the COVID-19 Pandemic: Longitudinal Smartphone and Ecological Momentary Assessment Study. J Med Internet Res. 2020;22(6):e20185-e.

129. Hudson GM, Sprow K. Promoting Physical Activity During the COVID-19 Pandemic: Implications for Obesity and Chronic Disease Management. J Phys Act Health. 2020:1-3.

130. Husain W, Ashkanani F. Does COVID-19 change dietary habits and lifestyle behaviours in Kuwait: a community-based cross-sectional study. Environ Health Prev Med. 2020;25(1):61-.

131. Ingram J, Maciejewski G, Hand CJ. Changes in Diet, Sleep, and Physical Activity Are Associated With Differences in Negative Mood During COVID-19 Lockdown. Front Psychol. 2020;11:588604.

132. Jeong H-C, Lee E-J, Youn H-S, So W-Y. Development and Implementation of a &quot;Music Beeps&quot; Program to Promote Physical Fitness in Adolescents. Int j environ res public health (Online). 2020;17(17).

133. Jia P, Zhang L, Yu W, Yu B, Liu M, Zhang D, et al. Impact of COVID-19 lockdown on activity patterns and weight status among youths in China: the COVID-19 Impact on Lifestyle Change Survey (COINLICS). International Journal of Obesity. 2021;45(3):695-9.

134. Jiménez-Pavón D, Carbonell-Baeza A, Lavie CJ. Physical exercise as therapy to fight against the mental and physical consequences of COVID-19 quarantine: Special focus in older people. Prog Cardiovasc Dis. 2020;63(3):386-8.

135. Jose P, Emanuel GD, Sebastian W, Veronica G, David W. Psychosocial aspects of the COVID-19 pandemic in the mendoza population. Revista Argentina de Cardiologia. 2020;88(3):203-6.

136. Kahyaoglu Sut H, Kucukkaya B. Anxiety, depression, and related factors in pregnant women during the COVID-19 pandemic in Turkey: A web-based cross-sectional study. Perspect psychiatr care. 2020.

137. Kalron A, Dolev M, Greenberg-Abrahami M, Menascu S, Frid L, Avrech-Shezifi S, et al. Physical activity behavior in people with multiple sclerosis during the COVID-19 pandemic in Israel: Results of an online survey. Multiple Sclerosis and Related Disorders. 2021;47:102603.

138. Kang S, Sun Y, Zhang X, Sun F, Wang B, Zhu W. Is Physical Activity Associated with Mental Health among Chinese Adolescents during Isolation in COVID-19 Pandemic? Journal of epidemiology and global health (Online). 2020.

139. Karuc J, Sorić M, Radman I, Mišigoj-Duraković M. Moderators of Change in Physical Activity Levels during Restrictions Due to COVID-19 Pandemic in Young Urban Adults. Sustainability. 2020;12(16):6392.

140. Katewongsa P, Widyastari DA, Saonuam P, Haemathulin N, Wongsingha N. The effects of the COVID-19 pandemic on the physical activity of the Thai population: Evidence from Thailand's Surveillance on Physical Activity 2020. J Sport Health Sci. 2020.

141. Kaux JF, Francaux M. [Physical activity during the Covid-19 pandemic]. Sci Sports. 2020;35(3):117-8.

142. Keel PK, Gomez MM, Harris L, Kennedy GA, Ribeiro J, Joiner TE. Gaining "The Quarantine 15:" Perceived versus observed weight changes in college students in the wake of COVID-19. Int J Eat Disord. 2020;53(11):1801-8.

143. Khader MA, Jabeen T, Namoju R. A cross sectional study reveals severe disruption in glycemic control in people with diabetes during and after lockdown in India. Diabetes Metab Syndr. 2020;14(6):1579-84.

144. Khan AH, Sultana MS, Hossain S, Hasan MT, Ahmed HU, Sikder MT. The impact of COVID-19 pandemic on mental health &amp; wellbeing among home-quarantined Bangladeshi students: A cross-sectional pilot study. J Affect Disord. 2020;277:121-8.

145. Kishimoto M, Ishikawa T, Odawara M. Behavioral changes in patients with diabetes during the COVID-19 pandemic. Diabetology International. 2020.

146. Koohsari MJ, Nakaya T, McCormack GR, Shibata A, Ishii K, Oka K. Changes in Workers' Sedentary and Physical Activity Behaviors in Response to the COVID-19 Pandemic and Their Relationships With Fatigue: Longitudinal Online Study. JMIR Public Health Surveill. 2021;7(3):e26293.

147. Kriaucioniene V, Bagdonaviciene L, Rodríguez-Pérez C, Petkeviciene J. Associations between Changes in Health Behaviours and Body Weight during the COVID-19 Quarantine in Lithuania: The Lithuanian COVIDiet Study. Nutrients. 2020;12(10).

148. Kumar N, Gupta R, Kumar H, Mehta S, Rajan R, Kumar D, et al. Impact of home confinement during COVID-19 pandemic on sleep parameters in Parkinson's disease. Sleep medicine. 2021;77:15-22.

149. Lazzeroni P, Motta M, Monaco S, Laudisio SR, Furoncoli D, Maffini V, et al. Improvement in glycaemic control in paediatric and young adult type 1 diabetes patients during COVID-19 pandemic: role of telemedicine and lifestyle changes. Acta Biomed. 2021;92(5):e2021399.

150. Lebel C, MacKinnon A, Bagshawe M, Tomfohr-Madsen L, Giesbrecht G. Elevated depression and anxiety symptoms among pregnant individuals during the COVID-19 pandemic. J Affect Disord. 2020;277:5-13.

151. Lehtisalo J, Palmer K, Mangialasche F, Solomon A, Kivipelto M, Ngandu T. Changes in lifestyle, behaviors, and risk factors for cognitive impairment in older persons during the first wave of the coronavirus disease 2019 pandemic in Finland: results from the FINGER study. Frontiers in Psychiatry. 2021;12:21.

152. Levinger P, Panisset M, Dunn J, Haines T, Dow B, Batchelor F, et al. Exercise interveNtion outdoor proJect in the cOmmunitY for older people - results from the ENJOY Seniors Exercise Park project translation research in the community. BMC Geriatr. 2020;20(1):446.

153. Lim S, Lim H, Després JP. Collateral Damage of the COVID-19 Pandemic on Nutritional Quality and Physical Activity: Perspective from South Korea. Obesity (Silver Spring). 2020;28(10):1788-90.

154. Limbers CA, McCollum C, Greenwood E. Physical activity moderates the association between parenting stress and quality of life in working mothers during the COVID-19 pandemic. Mental Health and Physical Activity. 2020;19(100358).

155. Lippi G, Henry BM, Sanchis-Gomar F. Physical inactivity and cardiovascular disease at the time of coronavirus disease 2019 (COVID-19). Eur J Prev Cardiol. 2020;27(9):906-8.

156. Liu Q, Zhou Y, Xie X, Xue Q, Zhu K, Wan Z, et al. The prevalence of behavioral problems among school-aged children in home quarantine during the COVID-19 pandemic in china. J Affect Disord. 2020;279:12550-.

157. Lopez-Bueno R, Calatayud J, Ezzatvar Y, Casajus JA, Smith L, Andersen LL, et al. Association Between Current Physical Activity and Current Perceived Anxiety and Mood in the Initial Phase of COVID-19 Confinement. Frontiers in Psychiatry. 2020;11(729).

158. López-Bueno R, López-Sánchez GF, Casajús JA, Calatayud J, Gil-Salmerón A, Grabovac I, et al. Health-Related Behaviors Among School-Aged Children and Adolescents During the Spanish Covid-19 Confinement. Front Pediatr. 2020;8:573.

159. López-Sánchez GF, López-Bueno R, Gil-Salmerón A, Zauder R, Skalska M, Jastrzębska J, et al. Comparison of physical activity levels in Spanish adults with chronic conditions before and during COVID-19 quarantine. Eur J Public Health. 2020.

160. Luciano F, Cenacchi V, Vegro V, Pavei G. COVID-19 lockdown: physical activity, sedentary behaviour and sleep in Italian medicine students. Eur J Sport Sci. 2020:1-22.

161. Machado CLF, Pinto RS, Brusco CM, Cadore EL, Radaelli R. COVID-19 pandemic is an urgent time for older people to practice resistance exercise at home. Exp Gerontol. 2020;141:111101.

162. Magnavita N, Soave PM, Ricciardi W, Antonelli M. Occupational Stress and Mental Health among Anesthetists during the COVID-19 Pandemic. Int J Environ Res Public Health. 2020;17(21).

163. Mahanty C, Kumar R, Mishra BK. Analyses the effects of COVID-19 outbreak on human sexual behaviour using ordinary least-squares based multivariate logistic regression. Qual Quant. 2020:1-21.

164. Maher JP, Hevel DJ, Reifsteck EJ, Drollette ES. Physical activity is positively associated with college students' positive affect regardless of stressful life events during the COVID-19 pandemic. Psychol Sport Exerc. 2021;52:101826.

165. Malanchini G, Malacrida M, Ferrari P, Leidi C, Ferrari G, Racheli M, et al. Impact of the Coronavirus Disease-19 Outbreak on Physical Activity of Patients With Implantable Cardioverter Defibrillators. J Card Fail. 2020;26(10):898-9.

166. Malta DC, Szwarcwald CL, Barros MBdA, Gomes CS, Machado ÍE, Souza Júnior PRBd, et al. A pandemia da COVID-19 e as mudanças no estilo de vida dos brasileiros adultos: um estudo transversal, 2020. Epidemiol Serv Saude. 2020;29(4):e2020407-e.

167. Mandelkorn U, Genzer S, Choshen-Hillel S, Reiter J, Meira E Cruz M, Hochner H, et al. Escalation of sleep disturbances amid the COVID-19 pandemic: a cross-sectional international study. J clin sleep med. 2020.

168. Marco-Ahulló A, Montesinos-Magraner L, González L-M, Morales J, Bernabéu-García JA, García-Massó X. Impact of COVID-19 on the self-reported physical activity of people with complete thoracic spinal cord injury full-time manual wheelchair users. The Journal of Spinal Cord Medicine. 2021:1-5.

169. Martinez EZ, Silva FM, Morigi TZ, Zucoloto ML, Silva TL, Joaquim AG, et al. Physical activity in periods of social distancing due to COVID-19: a cross-sectional survey. Cien Saude Colet. 2020;25:4157-68.

170. Matvienko-Sikar K, Pope J, Cremin A, Carr H, Leitao S, Olander EK, et al. Differences in levels of stress, social support, health behaviours, and stress-reduction strategies for women pregnant before and during the COVID-19 pandemic, and based on phases of pandemic restrictions, in Ireland. Women Birth. 2020.

171. McGuine TA, Biese KM, Petrovska L, Hetzel SJ, Reardon C, Kliethermes S, et al. The Health Of Us Adolescent Athletes During Covid-19 Related School Closures And Sport Cancellations. J Athl Train. 2020.

172. McQueenie R, Foster HME, Jani BD, Katikireddi SV, Sattar N, Pell JP, et al. Multimorbidity, polypharmacy, and COVID-19 infection within the UK Biobank cohort. PLoS One. 2020;15(8):e0238091.

173. Membrilla JA, de Lorenzo Í, Sastre M, Díaz de Terán J. Headache as a Cardinal Symptom of Coronavirus Disease 2019: A Cross-Sectional Study. Headache. 2020.

174. Meyer J, McDowell C, Lansing J, Brower C, Smith L, Tully M, et al. Changes in Physical Activity and Sedentary Behavior in Response to COVID-19 and Their Associations with Mental Health in 3052 US Adults. Int j environ res public health (Online). 2020;17(18).

175. Minsky NC, Pachter D, Zacay G, Chishlevitz N, Ben-Hamo M, Weiner D, et al. Managing Obesity in Lockdown: Survey of Health Behaviors and Telemedicine. Nutrients. 2021;13(4):1359.

176. Minuto N, Bassi M, Montobbio C, Vinci F, Mercuri C, Perri FN, et al. The Effect of Lockdown and Physical Activity on Glycemic Control in Italian Children and Young Patients With Type 1 Diabetes. Front Endocrinol (Lausanne). 2021;12:690222.

177. Mishra R, Park C, York MK, Kunik ME, Wung SF, Naik AD, et al. Decrease in Mobility during the COVID-19 Pandemic and Its Association with Increase in Depression among Older Adults: A Longitudinal Remote Mobility Monitoring Using a Wearable Sensor. Sensors (Basel). 2021;21(9).

178. Miyahara S, Tanikawa Y, Hirai H, Togashi S. Impact of the state of emergency enacted due to the COVID-19 pandemic on the physical activity of the elderly in Japan. J Phys Ther Sci. 2021;33(4):345-50.

179. Mon-López D, Bernardez-Vilaboa R, Fernandez-Balbuena AA, Sillero-Quintana M. The Influence of COVID-19 Isolation on Physical Activity Habits and Its Relationship with Convergence Insufficiency. Int J Environ Res Public Health. 2020;17(20).

180. Mon-López D, de la Rubia Riaza A, Hontoria Galán M, Refoyo Roman I. The Impact of Covid-19 and the Effect of Psychological Factors on Training Conditions of Handball Players. International Journal of Environmental Research and Public Health. 2020;17(18):6471.

181. Moore SA, Faulkner G, Rhodes RE, Brussoni M, Chulak-Bozzer T, Ferguson LJ, et al. Impact of the COVID-19 virus outbreak on movement and play behaviours of Canadian children and youth: a national survey. Int J Behav Nutr Phys Act. 2020;17(1):85-.

182. Mota IA, Oliveira Sobrinho GD, Morais IPS, Dantas TF. Impact of COVID-19 on eating habits, physical activity and sleep in Brazilian healthcare professionals. Arq Neuropsiquiatr. 2021;79(5):429-36.

183. Müller P, Achraf A, Zou L, Apfelbacher C, Erickson KI, Müller NG. COVID-19, physical (in-)activity, and dementia prevention. Alzheimers Dement (N Y). 2020;6(1):e12091-e.

184. Muniz-Pardos B, Shurlock J, Debruyne A, Steinacker JM, Borjesson M, Wolfarth B, et al. Collateral Health Issues Derived from the Covid-19 Pandemic. Sports Medicine - Open. 2020;6(35).

185. Muriel X C-IJ, Cerezuela-Espejo V, et al. Training load and performance impairments in professional cyclists during COVID-19 Lockdown. Int J Sports Physiol Perform 2020;1:1–41.

186. Nachimuthu S, Vijayalakshmi R, Sudha M, Viswanathan V. Coping with diabetes during the COVID - 19 lockdown in India: Results of an online pilot survey. Diabetes Metab Syndr. 2020;14(4):579-82.

187. Narici M, De Vito G, Franchi M, Paoli A, Moro T, Marcolin G, et al. Impact of sedentarism due to the COVID-19 home confinement on neuromuscular, cardiovascular and metabolic health: Physiological and pathophysiological implications and recommendations for physical and nutritional countermeasures. Eur J Sport Sci. 2020:1-22.

188. Nigg C, Oriwol D, Wunsch K, Burchartz A, Kolb S, Worth A, et al. Population density predicts youth's physical activity changes during Covid-19 - Results from the MoMo study. Health Place. 2021;70:102619.

189. Nilima N, Kaushik S, Tiwary B, Pandey PK. Psycho-social factors associated with the nationwide lockdown in India during COVID- 19 pandemic. 2020.

190. Nyenhuis SM, Greiwe J, Zeiger JS, Nanda A, Cooke A. Exercise and Fitness in the Age of Social Distancing During the COVID-19 Pandemic. J Allergy Clin Immunol Pract. 2020;8(7):2152-5.

191. Obuchi SP, Kawai H, Ejiri M, Ito K, Murakawa K. Change in outdoor walking behavior during the coronavirus disease pandemic in Japan: A longitudinal study. Gait Posture. 2021;88:42-6.

192. Okeahalam C, Williams V, Otwombe K. Factors associated with COVID-19 infections and mortality in Africa: a cross-sectional study using publicly available data. BMJ Open. 2020;10(11):e042750.

193. Okely JA, Corley J, Welstead M, Taylor AM, Page D, Skarabela B, et al. Change in Physical Activity, Sleep Quality, and Psychosocial Variables during COVID-19 Lockdown: Evidence from the Lothian Birth Cohort 1936. Int J Environ Res Public Health. 2020;18(1).

194. Onchonga D, Omwoyo J, Nyamamba D. Assessing the Prevalence of Self-Medication among Healthcare Workers before and during the 2019 SARS-CoV-2 (COVID-19) Pandemic in Kenya. Saudi pharmaceutical journal. 2020.

195. Ong JL, Lau T, Massar SAA, Chong ZT, Ng BKL, Koek D, et al. COVID-19 Related Mobility Reduction: Heterogenous Effects on Sleep and Physical Activity Rhythms. Sleep. 2020.

196. Paltrinieri S, Bressi B, Costi S, Mazzini E, Cavuto S, Ottone M, et al. Beyond lockdown: the potential side effects of the SARS-CoV-2 pandemic on public health. Nutrients. 2021;13(5):1600.

197. Pańczyk J, Kamecki H, Tchórzewska-Korba H, Szulerecki P, Rosa A, Sosnowski R. Maintaining physical activity in the era of COVID-19 pandemic: a chair-based exercise program for home-isolated elderly prostate cancer patients. Cent European J Urol. 2020;73(3):385-6.

198. Parcha V, Malla G, Suri SS, Kalra R, Heindl B, Berra L, et al. Geographic Variation of Racial Disparities in Health and COVID-19 Mortality. Mayo Clin Proc Innov Qual Outcomes. 2020.

199. Park JH, Yoo E, Kim Y, Lee JM. What Happened Pre- and during COVID-19 in South Korea? Comparing Physical Activity, Sleep Time, and Body Weight Status. Int J Environ Res Public Health. 2021;18(11).

200. Park S, Kim B, Lee J. Social Distancing and Outdoor Physical Activity During the COVID-19 Outbreak in South Korea: Implications for Physical Distancing Strategies. Asia Pac J Public Health. 2020;32(6):360-2.

201. Paz C, Mascialino G, Adana-Díaz L, Rodríguez-Lorenzana A, Simbaña-Rivera K, Gómez-Barreno L, et al. Behavioral and sociodemographic predictors of anxiety and depression in patients under epidemiological surveillance for COVID-19 in Ecuador. PLoS One. 2020;15(9):e0240008-e.

202. Pellegrini M, Ponzo V, Rosato R, Scumaci E, Goitre I, Benso A, et al. Changes in Weight and Nutritional Habits in Adults with Obesity during the “Lockdown” Period Caused by the COVID-19 Virus Emergency. Nutrients. 2020;12(7):2016.

203. Pépin JL, Bruno RM, Yang R-Y, Vercamer V, Jouhaud P, Escourrou P, et al. Wearable activity trackers for monitoring adherence to home confinement during the COVID-19 pandemic worldwide: data aggregation and analysis. Journal of medical Internet research. 2020;22(6):e19787.

204. Persiani P, De Meo D, Giannini E, Calogero V, Varsamis TS, Cavallo AU, et al. The aftermath of COVID-19 lockdown on daily life activities in orthopaedic patients. Journal of Pain Research. 2021;14:575.

205. Philip K, Cumella A, Farrington-Douglas J, Laffan M, Hopkinson N. Respiratory patient experience of measures to reduce risk of COVID-19: findings from a descriptive cross-sectional UK wide survey. BMJ Open. 2020;10(9):e040951-e.

206. Phillipou A, Meyer D, Neill E, Tan EJ, Toh WL, Van Rheenen TE, et al. Eating and exercise behaviors in eating disorders and the general population during the COVID-19 pandemic in Australia: Initial results from the COLLATE project. Int J Eat Disord. 2020;53(7):1158-65.

207. Pieh C, Budimir S, Delgadillo J, Barkham M, Fontaine JRJ, Probst T. Mental health during COVID-19 lockdown in the United Kingdom. Psychosom Med. 2020.

208. Pieh C, Budimir S, Probst T. The effect of age, gender, income, work, and physical activity on mental health during coronavirus disease (COVID-19) lockdown in Austria. J Psychosom Res. 2020;136:110186-.

209. Pietrobelli A, Pecoraro L, Ferruzzi A, Heo M, Faith M, Zoller T, et al. Effects of COVID-19 Lockdown on Lifestyle Behaviors in Children with Obesity Living in Verona, Italy: A Longitudinal Study. Obesity (Silver Spring). 2020;28(8):1382-5.

210. Pigaiani Y, Zoccante L, Zocca A, Arzenton A, Menegolli M, Fadel S, et al. Adolescent Lifestyle Behaviors, Coping Strategies and Subjective Wellbeing during the COVID-19 Pandemic: An Online Student Survey. Healthcare (Basel). 2020;8(4).

211. Pillay L, Janse van Rensburg DCC, Jansen van Rensburg A, Ramagole DA, Holtzhausen L, Dijkstra HP, et al. Nowhere to hide: The significant impact of coronavirus disease 2019 (COVID-19) measures on elite and semi-elite South African athletes. Journal of Science and Medicine in Sport. 2020;23(7):670-9.

212. Pinto AJ, Dunstan DW, Owen N, Bonfá E, Gualano B. Combating physical inactivity during the COVID-19 pandemic. Nat Rev Rheumatol. 2020;16(7):347-8.

213. Pišot S, Milovanović I, Šimunič B, Gentile A, Bosnar K, Prot F, et al. Maintaining everyday life praxis in the time of COVID-19 pandemic measures (ELP-COVID-19 survey). Eur J Public Health. 2020.

214. Pitanga FJG, Beck CC, Pitanga CPS. Physical Activity And Reducing Sedentary Behavior During The Coronavirus Pandemic. Arq Bras Cardiol. 2020.

215. Planchuelo-Gómez Á, Odriozola-González P, Irurtia MJ, de Luis-García R. Longitudinal evaluation of the psychological impact of the COVID-19 crisis in Spain. J Affect Disord. 2020;277:842-9.

216. Pombo A, Luz C, Rodrigues LP, Ferreira C, Cordovil R. Correlates of children&#039;s physical activity during the COVID-19 confinement in Portugal. Public Health. 2020;189:14-9.

217. Prada V, Hamedani M, Genovese F, Zuppa A, Benedetti L, Bellone E, et al. People with Charcot-Marie-Tooth disease and COVID-19: Impaired physical conditions due to the lockdown. An International cross-sectional survey. Annals of physical and rehabilitation medicine. 2020;63(6):557.

218. Pu B, Zhang L, Tang Z, Qiu Y. The Relationship between Health Consciousness and Home-Based Exercise in China during the COVID-19 Pandemic. Int J Environ Res Public Health. 2020;17(16).

219. Puhl RM, Lessard LM, Larson N, Eisenberg ME, Neumark-Stzainer D. Weight Stigma as a Predictor of Distress and Maladaptive Eating Behaviors During COVID-19: Longitudinal Findings From the EAT Study. Ann Behav Med. 2020;54(10):738-46.

220. Radtke T, Haile SR, Dressel H, Benden C. Recommended shielding against COVID-19 impacts physical activity levels in adults with cystic fibrosis. J Cyst Fibros. 2020.

221. Rastogi A, Hiteshi P, Bhansali A. Improved glycemic control amongst people with long-standing diabetes during COVID-19 lockdown: a prospective, observational, nested cohort study. Int J Diabetes Dev Ctries. 2020:1-6.

222. Reyes-Olavarría D, Latorre-Román PÁ, Guzmán-Guzmán IP, Jerez-Mayorga D, Caamaño-Navarrete F, Delgado-Floody P. Positive and Negative Changes in Food Habits, Physical Activity Patterns, and Weight Status during COVID-19 Confinement: Associated Factors in the Chilean Population. Int j environ res public health (Online). 2020;17(15).

223. Rezende DAN, Pinto AJ, Goessler KF, Nicoletti CF, Sieczkowska SM, Meireles K, et al. Influence of Adherence to Social Distancing Due to the COVID-19 Pandemic on Physical Activity Level in Post-bariatric Patients. Obes Surg. 2020:1-4.

224. Ribeiro de Lima JG, Abud GF, Freitas EC, Bueno Júnior CR. Effects of the COVID-19 pandemic on the global health of women aged 50 to 70 years. Exp Gerontol. 2021;150:111349.

225. Richardson DL, Duncan MJ, Clarke ND, Myers TD, Tallis J. The influence of COVID-19 measures in the United Kingdom on physical activity levels, perceived physical function and mood in older adults: A survey-based observational study. J Sports Sci. 2021;39(8):887-99.

226. Robinson E, Boyland E, Chisholm A, Harrold J, Maloney NG, Marty L, et al. Obesity, eating behavior and physical activity during COVID-19 lockdown: A study of UK adults. Appetite. 2021;156:104853.

227. Robinson E, Gillespie S, Jones A. Weight-related lifestyle behaviours and the COVID-19 crisis: An online survey study of UK adults during social lockdown. Obesity Science and Practice. 2020.

228. Rodríguez-Nogueira Ó, Leirós-Rodríguez R, Benítez-Andrades JA, Álvarez-Álvarez MJ, Marqués-Sánchez P, Pinto-Carral A. Musculoskeletal pain and teleworking in times of the COVID-19: analysis of the impact on the workers at two Spanish universities. International Journal of Environmental Research and Public Health. 2021;18(1):31.

229. Rogers NT, Waterlow NR, Brindle H, Enria L, Eggo RM, Lees S, et al. Behavioral Change Towards Reduced Intensity Physical Activity Is Disproportionately Prevalent Among Adults With Serious Health Issues or Self-Perception of High Risk During the UK COVID-19 Lockdown. Front Public Health. 2020;8:575091.

230. Romero-Blanco C, Rodríguez-Almagro J, Onieva-Zafra MD, Parra-Fernández ML, Prado-Laguna MDC, Hernández-Martínez A. Physical Activity and Sedentary Lifestyle in University Students: Changes during Confinement Due to the COVID-19 Pandemic. Int j environ res public health (Online). 2020;17(18).

231. Rossinot H, Fantin R, Venne J. Behavioral Changes During COVID-19 Confinement in France: A Web-Based Study. Int J Environ Res Public Health. 2020;17(22).

232. Rowlands AV, Henson JJ, Coull NA, Edwardson CL, Brady E, Hall A, et al. The impact of COVID‐19 restrictions on accelerometer‐assessed physical activity and sleep in individuals with type 2 diabetes. Diabetic Medicine. 2021;38(10):e14549.

233. Ruiz de Angulo D, Balaguer Román A, Munitiz Ruiz V, Gil Vázquez PJ, Ruiz Merino G, Ortiz Escandell M, et al. Influence of the lockdown due to COVID-19 on ponderal results during the first year after vertical gastrectomy. Cir Esp. 2020.

234. Ruiz-Roso MB, Knott-Torcal C, Matilla-Escalante DC, Garcimartín A, Sampedro-Nuñez MA, Dávalos A, et al. COVID-19 Lockdown and Changes of the Dietary Pattern and Physical Activity Habits in a Cohort of Patients with Type 2 Diabetes Mellitus. Nutrients. 2020;12(8).

235. Šagát P, Bartík P, Prieto González P, Tohănean DI, Knjaz D. Impact of COVID-19Quarantine on Low Back Pain Intensity, Prevalence, and Associated Risk Factors among Adult Citizens Residing in Riyadh (Saudi Arabia): A Cross-Sectional Study. Int J Environ Res Public Health. 2020;17(19).

236. Sanchez-Larsen A, Gonzalez-Villar E, Díaz-Maroto I, Layos-Romero A, Martínez-Martín Á, Alcahut-Rodriguez C, et al. Influence of the COVID-19 outbreak in people with epilepsy: Analysis of a Spanish population (EPICOVID registry). Epilepsy Behav. 2020;112:107396-.

237. Sánchez-Sánchez E, Ramírez-Vargas G, Avellaneda-López Y, Orellana-Pecino JI, García-Marín E, Díaz-Jimenez J. Eating Habits and Physical Activity of the Spanish Population during the COVID-19 Pandemic Period. Nutrients. 2020;12(9).

238. Sankar P, Ahmed WN, Mariam Koshy V, Jacob R, Sasidharan S. Effects of COVID-19 lockdown on type 2 diabetes, lifestyle and psychosocial health: A hospital-based cross-sectional survey from South India. Diabetes Metab Syndr. 2020;14(6):1815-9.

239. Sassone B, Mandini S, Grazzi G, Mazzoni G, Myers J, Pasanisi G. Impact of COVID-19 Pandemic on Physical Activity in Patients With Implantable Cardioverter-Defibrillators. J Cardiopulm Rehabil Prev. 2020;40(5):285-6.

240. Savage MJ, Hennis PJ, Magistro D, Donaldson J, Healy LC, James RM. Nine Months into the COVID-19 Pandemic: A Longitudinal Study Showing Mental Health and Movement Behaviours Are Impaired in UK Students. Int J Environ Res Public Health. 2021;18(6).

241. Savage MJ, James R, Magistro D, Donaldson J, Healy LC, Nevill M, et al. Mental health and movement behaviour during the COVID-19 pandemic in UK university students: Prospective cohort study. Mental Health and Physical Activity. 2020;19(100357).

242. Schirinzi T, Di Lazzaro G, Salimei C, Cerroni R, Liguori C, Scalise S, et al. Physical Activity Changes and Correlate Effects in Patients with Parkinson's Disease during COVID-19 Lockdown. Movement Disorders Clinical Practice. 2020;7(7):797-802.

243. Schlegl S, Maier J, Meule A, Voderholzer U. Eating disorders in times of the COVID-19 pandemic-Results from an online survey of patients with anorexia nervosa. Int J Eat Disord. 2020;53(11):1791-800.

244. Schlichtiger J, Brunner S, Steffen J, Huber BC. Mental health impairment triggered by the COVID-19 pandemic in a sample population of German students. J invest med. 2020.

245. Schlichtiger J, Steffen J, Huber BC, Brunner S. Physical activity during COVID-19 lockdown in older adults. J Sports Med Phys Fitness. 2020.

246. Schnitzer M, Schöttl SE, Kopp M, Barth M. COVID-19 stay-at-home order in Tyrol, Austria: sports and exercise behaviour in change? Public Health. 2020;185:218-20.

247. Schuch FB, Bulzing RA, Meyer J, Vancampfort D, Firth J, Stubbs B, et al. Associations of moderate to vigorous physical activity and sedentary behavior with depressive and anxiety symptoms in self-isolating people during the COVID-19 pandemic: A cross-sectional survey in Brazil. Psychiatry Res. 2020;292:113339-.

248. Sekulic D, Blazevic M, Gilic B, Kvesic I, Zenic N. Prospective Analysis of Levels and Correlates of Physical Activity during COVID-19 Pandemic and Imposed Rules of Social Distancing; Gender Specific Study among Adolescents from Southern Croatia. Sustainability. 2020;12(10):4072.

249. Serin E, Koc MC. Examination of the eating behaviours and depression states of the university students who stay at home during the coronavirus pandemic in terms of different variables. Progress in Nutrition. 2020;22:33-43.

250. Sfendla A, Hadrya F. Factors Associated with Psychological Distress and Physical Activity During the COVID-19 Pandemic. Health Secur. 2020.

251. Shah T, Purohit G. COVID-19-awareness, attitude & life style modifications among undergraduate health professional students of sumandeep vidyapeeth deemed to be university: A questionnaire based online survey. International Journal of Pharmaceutical Research. 2020;12:552-7.

252. Shalash A, Roushdy T, Essam M, Fathy M, Dawood NL, Abushady EM, et al. Mental Health, Physical Activity, and Quality of Life in Parkinson&#039;s Disease During COVID-19 Pandemic. Mov Disord. 2020;35(7):1097-9.

253. Song J, Ahn JH, Choi I, Mun JK, Cho JW, Youn J. The changes of exercise pattern and clinical symptoms in patients with Parkinson's disease in the era of COVID-19 pandemic. Parkinsonism Relat Disord. 2020;80:148-51.

254. Sonza A, da Cunha de Sá-Caputo D, Sartorio A, Tamini S, Seixas A, Sanudo B, et al. COVID-19 Lockdown and the behavior change on physical Exercise, pain and psychological well-being: an international multicentric study. International Journal of Environmental Research and Public Health. 2021;18(7):3810.

255. Srivastav AK, Sharma N, Samuel AJ. Impact of Coronavirus disease-19 (COVID-19) lockdown on physical activity and energy expenditure among physiotherapy professionals and students using web-based open E-survey sent through WhatsApp, Facebook and Instagram messengers: Impact of COVID-19 lockdown on physical activity and energy expenditure. Clinical Epidemiology and Global Health. 2020.

256. Stanton R, To QG, Khalesi S, Williams SL, Alley SJ, Thwaite TL, et al. Depression, Anxiety and Stress during COVID-19: Associations with Changes in Physical Activity, Sleep, Tobacco and Alcohol Use in Australian Adults. Int j environ res public health (Online). 2020;17(11).

257. Stein R. Physical Exercise in Patients with Heart Disease and in the General Population in Times of Coronavirus. Arq Bras Cardiol. 2020;114(5):827-8.

258. Suzuki Y, Maeda N, Hirado D, Shirakawa T, Urabe Y. Physical Activity Changes and Its Risk Factors among Community-Dwelling Japanese Older Adults during the COVID-19 Epidemic: Associations with Subjective Well-Being and Health-Related Quality of Life. Int j environ res public health (Online). 2020;17(18).

259. Szabó T, Stocker M, Ács P, Morvay-Sey K, Pálvölgyi Á, Laczkó T. Impact of COVID-19 on the physical activity and well-being of hungarian athletes and sports professionals Health Problems of Civilization. 2020;14(3):165-73.

260. Tanaka N, Hamamoto Y, Kurotobi Y, Yamasaki Y, Nakatani S, Matsubara M, et al. Lifestyle changes as a result of COVID-19 containment measures: Bodyweight and glycemic control in patients with diabetes in the Japanese declaration of a state of emergency. J Diabetes Investig. 2021;12(9):1718-22.

261. Taylor JK, Ndiaye H, Daniels M, Ahmed F. Lockdown, slow down: impact of the COVID-19 pandemic on physical activity-an observational study. Open Heart. 2021;8(1).

262. Ten Velde G, Lubrecht J, Arayess L, van Loo C, Hesselink M, Reijnders D, et al. Physical activity behaviour and screen time in Dutch children during the COVID-19 pandemic: Pre-, during- and post-school closures. Pediatr Obes. 2021;16(9):e12779.

263. Tison GH, Avram R, Kuhar P, Abreau S, Marcus GM, Pletcher MJ, et al. Worldwide Effect of COVID-19 on Physical Activity: A Descriptive Study. Ann Intern Med. 2020;173(9):767-70.

264. To QG, Duncan MJ, Van Itallie A, Vandelanotte C. Impact of COVID-19 on Physical Activity Among 10,000 Steps Members and Engagement With the Program in Australia: Prospective Study. J Med Internet Res. 2021;23(1):e23946.

265. Tornese G, Ceconi V, Monasta L, Carletti C, Faleschini E, Barbi E. Glycemic Control in Type 1 Diabetes Mellitus During COVID-19 Quarantine and the Role of In-Home Physical Activity. Diabetes Technol Ther. 2020;22(6):462-7.

266. Traunmüller C, Stefitz R, Gaisbachgrabner K, Schwerdtfeger A. Psychological correlates of COVID-19 pandemic in the Austrian population. BMC Public Health. 2020;20(1):1395.

267. Tucker JM, Welk GJ, Beyler NK. Physical activity in U.S.: adults compliance with the Physical Activity Guidelines for Americans. Am J Prev Med. 2011;40(4):454-61.

268. Turgut M, Soylu Y, Metin SN. Physical activity, night eating, and mood state profiles of athletes during the COVID-19 pandemic. Progress in Nutrition. 2020;22.

269. van Bakel BMA, Bakker EA, de Vries F, Thijssen DHJ, Eijsvogels TMH. Impact of COVID-19 lockdown on physical activity and sedentary behaviour in Dutch cardiovascular disease patients. Neth Heart J. 2021;29(5):273-9.

270. van de Venis L, van de Warrenburg BP, Weerdesteyn V, van Lith BJ, Geurts AC, Nonnekes J. COVID-19 reveals influence of physical activity on symptom severity in hereditary spastic paraplegia. Journal of Neurology. 2020;267(12):3462-4.

271. van der Heide A, Meinders MJ, Bloem BR, Helmich RC. The Impact of the COVID-19 Pandemic on Psychological Distress, Physical Activity, and Symptom Severity in Parkinson's Disease. J Parkinsons Dis. 2020;10(4):1355-64.

272. Vancini RL, Camargo-Neto L, de Lira CAB, Andrade MS, Viana RB, Nikolaidis PT, et al. Physical Activity and Sociodemographic Profile of Brazilian People during COVID-19 Outbreak: An Online and Cross-Sectional Survey. Int j environ res public health (Online). 2020;17(21).

273. Verma A, Rajput R, Verma S, Balania VKB, Jangra B. Impact of lockdown in COVID 19 on glycemic control in patients with type 1 Diabetes Mellitus. Diabetes Metab Syndr. 2020;14(5):1213-6.

274. Vetrovsky T, Frybova T, Gant I, Semerad M, Cimler R, Bunc V, et al. The detrimental effect of COVID-19 nationwide quarantine on accelerometer-assessed physical activity of heart failure patients. ESC Heart Fail. 2020;7(5):2093-7.

275. von Humboldt S, Mendoza-Ruvalcaba NM, Arias-Merino ED, Costa A, Cabras E, Low G, et al. Smart technology and the meaning in life of older adults during the Covid-19 public health emergency period: a cross-cultural qualitative study. Int Rev Psychiatry. 2020:1-10.

276. Waki K, Sankoda A, Amano S, Ogawa M, Ohe K. Responding to COVID-19: Agile Use of Information Technology to Serve Patients With Diabetes. J Diabetes Sci Technol. 2020;14(4):807-8.

277. Wang J, Gong Y, Chen Z, Wu J, Feng J, Yan S, et al. Sleep disturbances among Chinese residents during the Coronavirus Disease 2019 outbreak and associated factors. Sleep Med. 2020;74:199-203.

278. Wang X, Lei SM, Le S, Yang Y, Zhang B, Yao W, et al. Bidirectional Influence of the COVID-19 Pandemic Lockdowns on Health Behaviors and Quality of Life among Chinese Adults. Int J Environ Res Public Health. 2020;17(15).

279. Wang Y, Zhang Y, Bennell K, White DK, Wei J, Wu Z, et al. Physical Distancing Measures and Walking Activity in Middle-aged and Older Residents in Changsha, China, During the COVID-19 Epidemic Period: Longitudinal Observational Study. J Med Internet Res. 2020;22(10):e21632.

280. Weaver RH, Jackson A, Lanigan J, Power TG, Anderson A, Cox AE, et al. Health Behaviors at the Onset of the COVID-19 Pandemic. Am J Health Behav. 2021;45(1):44-61.

281. Werneck AO, Silva DR, Malta DC, Lima MG, Souza-Júnior PRB, Azevedo LO, et al. The mediation role of sleep quality in the association between the incidence of unhealthy movement behaviors during the COVID-19 quarantine and mental health. Sleep Med. 2020;76:10-5.

282. Werneck AO, Silva DR, Malta DC, Souza-Júnior PRB, Azevedo LO, Barros MBA, et al. Changes in the clustering of unhealthy movement behaviors during the COVID-19 quarantine and the association with mental health indicators among Brazilian adults. Translational behavioral medicine (Internet). 2020.

283. Wilson OWA, Holland KE, Elliott LD, Duffey M, Bopp M. The Impact of the COVID-19 Pandemic on US College Students' Physical Activity and Mental Health. J Phys Act Health. 2021;18(3):272-8.

284. Woodruff SJ, Coyne P, St-Pierre E. Stress, physical activity, and screen-related sedentary behaviour within the first month of the COVID-19 pandemic. Appl Psychol Health Well Being. 2021;13(2):454-68.

285. World Health Organization. Impact of COVID-19 on people's livelihoods, their health and our food systems. Joint statement by ILO, FAO, IFAD and WHO Switwerland2020 [Available from: <https://www.who.int/news/item/13-10-2020-impact-of-covid-19-on-people's-livelihoods-their-health-and-our-food-systems>.

286. Wu X, Luo S, Zheng X, Ding Y, Wang S, Ling P, et al. Glycemic control in children and teenagers with type 1 diabetes around lockdown for COVID-19: A continuous glucose monitoring-based observational study. J Diabetes Investig. 2021;12(9):1708-17.

287. Wunsch K, Nigg C, Niessner C, Schmidt SCE, Oriwol D, Hanssen-Doose A, et al. The Impact of COVID-19 on the Interrelation of Physical Activity, Screen Time and Health-Related Quality of Life in Children and Adolescents in Germany: Results of the Motorik-Modul Study. Children (Basel). 2021;8(2).

288. Wurm R, Parvizi T, Silvaeih S, Berger-Sieczkowski E, Goeschl S, König T, et al. Reduction of physical activity during the COVID-19 pandemic is related to increased neuropsychiatric symptoms in memory clinic patients. Clin Med (Lond). 2022;22(2):177-80.

289. Xiao S, Yan Z, Zhao L. Physical Activity, Screen Time, and Mood Disturbance Among Chinese Adolescents During COVID-19. J Psychosoc Nurs Ment Health Serv. 2020:1-7.

290. Yamada M, Kimura Y, Ishiyama D, Otobe Y, Suzuki M, Koyama S, et al. Effect of the COVID-19 Epidemic on Physical Activity in Community-Dwelling Older Adults in Japan: A Cross-Sectional Online Survey. J Nutr Health Aging. 2020;24(9):948-50.

291. Yan AF, Sun X, Zheng J, Mi B, Zuo H, Ruan G, et al. Perceived risk, behavior changes and Health-related outcomes during COVID-19 pandemic: Findingsamong adults with and without diabetesin China. Diabetes Res Clin Pract. 2020;167:108350-.

292. Yang S, Guo B, Ao L, Yang C, Zhang L, Zhou J, et al. Obesity and activity patterns before and during COVID-19 lockdown among youths in China. Clin Obes. 2020;10(6):e12416.

293. Yang Y, Koenigstorfer J. Determinants of physical activity maintenance during the Covid-19 pandemic: a focus on fitness apps. Transl Behav Med. 2020;10(4):835-42.

294. Zachary Z, Brianna F, Brianna L, Garrett P, Jade W, Alyssa D, et al. Self-quarantine and weight gain related risk factors during the COVID-19 pandemic. Obes Res Clin Pract. 2020;14(3):210-6.

295. Zaworski K, Kubińska Z, Dziewulska A, Walasek O. Physical activity of Poles in the care for their health potential before and during the COVID-19 pandemic. Disaster Med Public Health Prep. 2020:1-13.

296. Zenic N, Taiar R, Gilic B, Blazevic M, Maric D, Pojskic H, et al. Levels and Changes of Physical Activity in Adolescents during the COVID-19 Pandemic: Contextualizing Urban vs. Rural Living Environment. Applied sciences. 2020;10(11):3997.

297. Zhang J, Zhang Y, Huo S, Ma Y, Ke Y, Wang P, et al. Emotional Eating in Pregnant Women during the COVID-19 Pandemic and Its Association with Dietary Intake and Gestational Weight Gain. Nutrients. 2020;12(8).

298. Zhang S, Yu L. Practical home-based physical activity programs for university students during the COVID-19 pandemic. J Sports Med Phys Fitness. 2020.

299. Zhang X, Liu J, Han N, Yin J. Social Media Use, Unhealthy Lifestyles, and the Risk of Miscarriage Among Pregnant Women During the COVID-19 Pandemic: Prospective Observational Study. JMIR Public Health Surveill. 2021;7(1):e25241.

300. Zhang X, Zhu W, Kang S, Qiu L, Lu Z, Sun Y. Association between Physical Activity and Mood States of Children and Adolescents in Social Isolation during the COVID-19 Epidemic. Int J Environ Res Public Health. 2020;17(20).

301. Zhu Q, Li M, Ji Y, Shi Y, Zhou J, Li Q, et al. “Stay-at-Home” lifestyle effect on weight gain during the COVID-19 outbreak confinement in China. International Journal of Environmental Research and Public Health. 2021;18(4):1813.

302. Znazen H, Slimani M, Bragazzi NL, Tod D. The Relationship between Cognitive Function, Lifestyle Behaviours and Perception of Stress during the COVID-19 Induced Confinement: Insights from Correlational and Mediation Analyses. Int J Environ Res Public Health. 2021;18(6).

303. Zorcec T, Jakovska T, Micevska V, Boskovska K, Cholakovska VC. Pandemic with COVID-19 and Families with Children with Chronic Respiratory Diseases. Pril (Makedon Akad Nauk Umet Odd Med Nauki). 2020;41(2):95-101.

# **Table S4: Physical activity prevalence among children during COVID-19**

| **Reference** | **Country** | **Study design** | **Sampling method** | **Setting** | **Pre or during COVID19** | **Restrictions during the COVID19 Pandemic** | **Data collection time** | | **Population description** | **Physical activity instrument or used item** | **Level of physical activity** | | **Prevalence of physical activity (%)** | |  |
| --- | --- | --- | --- | --- | --- | --- | --- | --- | --- | --- | --- | --- | --- | --- | --- |
| Alonso-Martinez, 2021(4) | Spain | Cohort | NR | Community | Pre | No restrictions | Sep to Dec, 2019 | | GP, M: 70 (56.9%)  F: 53 (43.1%), Mean age (SD): 4.27 (0.84) | GENE Activ tri-axial accelerometer | MVPA | | 77.9 | |  |
| Alonso-Martinez, 2021(4) | Spain | Cohort | NR | Community | During | Mandatory home lockdown | Mar to Apr, 2020 | | GP, M: 73 (50.4%)  F: 53 (49.6%), Mean age (SD): 4.29 (0.76) | GENE Activ tri-axial accelerometer | MVPA | | 85.7 | |  |
| Bronikowska, 2021 (5) | Poland | Longitudinal | NR | Community | Pre | No restrictions | Feb 2020 | | GP, M: 61(48%)  F: 66 (52%), Mean age (SD): 15.4(0.5) | Physical Activity Screening Measure (electronic survey) | MVPA | | 23.6 | |  |
| Bronikowska, 2021 (5) | Poland | Longitudinal | NR | Community | During | National restrictions | End of Jun, 2020 | | GP, M: 61(48%)  F: 66 (52%), Mean age (SD): 15.4(0.5) | Physical Activity Screening Measure (electronic survey) | MVPA | | 22 | |  |
| Brze ̨k, 2021(6) | Poland | Cohort | NR | Community | Pre | No restrictions | NR | | GP, M: 584 (44.4%)  F: 732 (55.6%), 3-5years | Questionnaire survey | MVPA | | 81 | |  |
| Brze ̨k, 2021 (6) | Poland | Cohort | NR | Community | Pre | No restrictions | NR | | GP, 3-year-old | Questionnaire survey | MVPA | | 24.62 | |  |
| Brze ̨k, 2021 (6) | Poland | Cohort | NR | Community | Pre | No restrictions | NR | | GP, 4-year-olds | Questionnaire survey | MVPA | | 38.97 | |  |
| Brze ̨k, 2021 (6) | Poland | Cohort | NR | Community | Pre | No restrictions | NR | | GP, 5-year-olds | Questionnaire survey | MVPA | | 34.51 | |  |
| Brze ̨k, 2021 (6) | Poland | Cohort | NR | Community | During | Restrictions: ban on social gatherings outside of the household | May-Jun, 2020 | | GP, M: 584 (44.4%)  F: 732 (55.6%), 3-5years | Questionnaire survey | MVPA | | 67 | |  |
| Brze ̨k, 2021 (6) | Poland | Cohort | NR | Community | During | Restrictions: ban on social gatherings outside of the household | May-Jun, 2020 | | GP, 3-year-old | Questionnaire survey | MVPA | | 18.46 | |  |
| Brze ̨k, 2021 (6) | Poland | Cohort | NR | Community | During | Restrictions: ban on social gatherings outside of the household | May-Jun, 2020 | | GP, 4-year-olds | Questionnaire survey | MVPA | | 22.08 | |  |
| Brze ̨k, 2021(6) | Poland | Cohort | NR | Community | During | Restrictions: ban on social gatherings outside of the household | May-Jun, 2020 | | GP, 5-year-olds | Questionnaire survey | MVPA | | 22.2 | |  |
| Chaffee, 2021 (7) | Northern California | Prospective cohort analysis | NR | Community | Pre | No restrictions | Mar 2019- Feb 2020 | | GP, 14-15 years | Questionnaire survey | MVPA | | 53.5 | |  |
| Chaffee, 2021(7) | Northern California | Prospective cohort analysis | NR | Community | During | Restrictions, stay-at-home-order | Mar 2020 to Jun, 2020 | | GP, 14-15 years | Questionnaire survey | MVPA | | 39 | |  |
| Constantini, 2021 (8) | Israel | CS | Non-probability (self-selection online survey) | Community | During | Restrictions, lockdown | 10 Apr 2020 to 5 May, 2020 | | GP, M: 231 (69.4%)  F: 102 (30.6%), Mean age (SD): 17.4 (0.8) | The Health Behaviours in School-aged Children survey (HBSC) | MVPA | | 10.6 | |  |
| Francisco, 2020 (9) | Italy | CS | snowball sampling | Online survey | Before | NR | NR | | GP, F: 351(49.3%)  M: 361(50.7%), 9.40 (4.46) | Not a Validated Questionnaire | MVPA | | 47.2 | |  |
| Francisco, 2020 (9) | Italy | CS | snowball sampling | Online survey | During | During the early phase of the quarantine associated with COVID-19 from parents’ perspective | Mar-Apr | | GP, F: 351(49.3%)  M: 361(50.7%), 9.40 (4.46) | Not a Validated Questionnaire | MVPA | | 15.0 | |  |
| Francisco, 2020 (9) | Spain | CS | snowball sampling | Online Survey | Before | NR | NR | | GP, F: 192 (44.5%)  M: 239(55.5%), 8.55 (3.73) | Non validated questionnaire | MVPA | | 65.7 | |  |
| Francisco, 2020 (9) | Spain | CS | snowball sampling | Online Survey | During | During the early phase of the quarantine associated with COVID-19 from parents’ perspective | Mar-Apr | | GP, F:192(44.5%)  M:239(55.5%), 8.55 (3.73) | Non validated questionnaire | MVPA | | 14.4 | |  |
| Francisco, 2020 (9) | Portugal | CS | snowball sampling | Online Survey | Before | NR | NR | | GP, F:156 (46.6)  M:179 (53.4%), 9.42 (4.45) | Non validated questionnaire | MVPA | | 54.3 | |  |
| Francisco, 2020 (9) | Portugal | CS | snowball sampling | Online Survey | During | During the early phase of the quarantine associated with COVID-19 from parents’ perspective | Mar-Apr | | GP, F:156 (46.6)  M:179 (53.4%), 9.42 (4.45) | Non validated questionnaire | MVPA | | 13.7 | |  |
| Francisco, 2020 (9) | Italy, Spain, Portugal | CS | snowball sampling | Online Survey | Before | No restrictions | Mar-Apr | | GP, F:699 (47.2%)  M:781 (52.8), Mean (SD): 9.15 (4.27) | Non validated questionnaire | MVPA | | 54.1 | |  |
| Francisco, 2020 (9) | Italy, Spain, Portugal | CS | snowball sampling | Online Survey | During | During the early phase of the quarantine associated with COVID-19 from parents’ perspective | Mar-Apr | | GP, F:699 (47.2%)  M:781 (52.8), Mean age (SD): 9.15 (4.27) | Non validated questionnaire | MVPA | | 14.7 | |  |
| Kovacs, 2021(10) | 10 Countries:  Germany  Hungary  Poland  Russia Federation  Slovenia  Spain  France  Italy  Portugal  Romania | CS | Convenience | Online questionnaire | During COVID | Restrictions | 15 May to 22 Jun 2020 | | GP, M: 3940 (46.9%)  F: 4455 (53.1%), Age: 10-18 Years | PAQ | MVPA | | 19 | |  |
| Kovacs, 2021 (10) | Germany | CS | Convenience | Online questionnaire | During COVID | Restrictions:  Outdoor sports without physical contact resumed and schools re-opened with special rules | 15 May to 22 Jun 2020 | | GP, M: 140 (58.1%)  F: 101 (41.9%), Age: 8-12 Years | PAQ | MVPA | | 17.8 | |  |
| Kovacs, 2021(10) | Hungary | CS | Convenience | Online questionnaire | During COVID | Restrictions:  All places and school places remained closed except with some reopening in the capital | 15 May to 22 Jun 2020 | | GP, M: 1243 (47.3%)  F: 1383 (52.7%, Age: 11-16 Years | PAQ | MVPA | | 19.9 | |  |
| Kovacs, 2021 (10) | Russian Federation | CS | Convenience | Online questionnaire | During COVID | Restrictions:  Schools closed nationwide | 15 May to 22 Jun 2020 | | GP, M: 150 (47.6%)  F: 165 (52.4%), Age: 9-14 Years | PAQ | MVPA | | 21 | |  |
| Kovacs, 2021 (10) | Poland | CS | Convenience | Online questionnaire | During COVID | Restrictions:  Conservative stay-at-home order | 15 May to 22 Jun 2020 | | GP, M: 264 (50.4%)  F: 259 (49.6%), Age: 8-13 Years | PAQ | MVPA | | 17.2 | |  |
| Kovacs, 2021 (10) | Slovenia | CS | Convenience | Online questionnaire | During COVID | Restrictions:  Mid-May re-opened playgrounds, parks, school for younger children and indoor facilities stayed closed | 15 May to 22 Jun 2020 | | GP, M: 802 (42.3%)  F: 1095 (57.7%), Age: 11-14 Years | PAQ | MVPA | | 26.7 | |  |
| Kovacs, 2021 (10) | Spain | CS | Convenience | Online questionnaire | During COVID | Restrictions | 15 May to 22 Jun 2020 | | GP, M: 446 (49.9%)  F: 448 (50.1%), Age: 9-14 Years | PAQ | MVPA | | 18.1 | |  |
| Kovacs, 2021 (10) | France | CS | Convenience | Online questionnaire | During COVID | Restrictions:  55-day home confinement with closure of public places ended on May 11, 2020 | 15 May to 22 Jun 2020 | | GP, M: 116 (55.5%)  F: 90 (44.5%), Age: 9-16 Years | PAQ | MVPA | | 15.3 | |  |
| Kovacs, 2021 (10) | Italy | CS | Convenience | Online questionnaire | During COVID | Restrictions | 15 May to 22 Jun 2020 | | GP, M: 83(34.6%)  F: 157(65.4%), Age: 11-17 Years | PAQ | MVPA | | 7.5 | |  |
| Kovacs, 2021 (10) | Portugal | CS | Convenience | Online questionnaire | During COVID | Restrictions:  Partial school openings | 15 May to 22 Jun 2020 | | GP, M: 558(48.3%)  F: 598(51.7%), Age: 11-16 Years | PAQ | MVPA | | 7.6 | |  |
| Kovacs, 2021 (10) | Romania | CS | Convenience | Online questionnaire | During COVID | Restrictions:  Schools closed nationwide | 15 May to 22 Jun 2020 | | GP, M: 138(46.9%)  F: 156(53.1%), Age: 11-14 Years | PAQ | MVPA | | 23.5 | |  |
| Medrano, 2020 (11) | Spain | LOS | Probabilistic, random four-stage sampling | Community | Before | No restrictions | Sep-Dec, 2019 | | GP, M: 149 (51.3%)  F: 142 (48.7%), Mean age (SD): 12.1 (2.4)  Age range: 8-16 | Youth Activity Profile questionnaire (YAP) & wrist-worn accelerometer (ActiGraph, Pensacola, FL) | MVPA | | 40.2 | |  |
| Medrano, 2020 (11) | Spain | LOS | Probabilistic, random four-stage sampling | Online survey | During | Confinement | Mar-Apr, 2020 | | GP, M: 58 (51.3%)  F: 55 (48.7%), Mean age (SD): 12.0 (2.6)  Age range: 8-16 | Youth Activity Profile” questionnaire (YAP) & wrist-worn accelerometer (ActiGraph, Pensacola, FL) | MVPA | | 49.4 | |  |
| Mitra, 2020 (12) | Canada | CS | Non-probabilistic, Quota sampling | Online survey | During | One month after the announcement of the global pandemic and at the height of restrictions | Apr 2020 | | GP, M: 780 (53%)  F: 692 (47%), Age range: 5-17 | Not reported | MVPA | | 18.2 | |  |
| Munasinghe, 2020 (13) | Australia | Prospective Cohort study | . | Online survey | Before | No restrictions | Nov 18, 2019- Apr 19, 2020 | | GP, M: 102 (17.5%)  F: 465 (79.9%)  Unk: 2.6%, Median age (IQR): 17 (16-18)  Age range: 13-19 | PACE + Adolescent Physical Activity Measures | MVPA | | 48.61 | |  |
| Munasinghe, 2020 (13) | Australia | Prospective Cohort study | . | Online survey | During | Physical distancing policies and school closures | Nov 18, 2019-Apr 19, 2020 | | GP, M: 102 (17.5%)  F: 465 (79.9%)  Unk: 2.6%, Median age (IQR): 17 (16-18)  Age range: 13-19 | PACE + Adolescent Physical Activity Measures | MVPA | | 43.64 | |  |
| Okely, 2021 (14) | Australia, Bangladesh, China, Hong Kong, India, Indonesia, Malaysia, Morocco, Pakistan, Spain, Sri Lanka, Sweden, United States, Vietnam | Longitudinal | Multistage stratified Random | Online survey/self-administrated | Pre | No restrictions | Apr-Mar | | GP, F: 415(49%)  M: 432(51%), 3–4-year-old | The Sunrise study: International study of 24 hr movement behaviours in the early years’ parent/ caregiver survey | MVPA | | 50.8 | |  |
| Okely, 2021 (14) | Australia, Bangladesh, China, Hong Kong, India, Indonesia, Malaysia, Morocco, Pakistan, Spain, Sri Lanka, Sweden, United States, Vietnam | Longitudinal | Multistage stratified Random | Online survey/self-administrated | During | At the height of COVID-19 restrictions | May–Jun | | GP, F: 415 (49%)  M: 432 (51%), 6-18 years | The Sunrise study: International study of 24 hr movement behaviours in the early years’ parent/ caregiver survey | MVPA | | 48.7 | |  |
| Orgilés, 2020 (15) | Italy | CS | Snowball sampling strategy | Online survey | During | Before the Quarantine | Mar 25-Apr 7 | | GP, F: 543, Age 3-18 Y | Piloted Questionnaire | MVPA | | 47 | |  |
| Orgilés, 2020 (15) | Spain | CS | Snowball sampling strategy | Online survey | During | Before the Quarantine | Mar 31-Apr 15 | | GP, F: 543 (47.5)  M: 600 (52.5), Age: 3-18 Y | Piloted Questionnaire | MVPA | | 66 | |  |
| Orgilés, 2020 (15) | Italy | CS | Snowball sampling strategy | Online survey | During | During Quarantine | Mar 25-Apr 7 | | GP, F: 543 (47.5)  M: 600 (52.5), Age: 3-18 Y | Piloted Questionnaire | MVPA | | 13 | |  |
| Orgilés, 2020 (15) | Spain | CS | Snowball sampling strategy | Online survey | During | During Quarantine | Mar 31-Apr 15 | | GP, F: 543 (47.5)  M: 600 (52.5), Age: 3-18 Y | Piloted Questionnaire | MVPA | | 14 | |  |
| Parker, 2021 (16) | Australia | Longitudinal | Snowball sampling technique | Online survey | During | Stay-at-home period | Apr or May | | GP, F: 685 (71.1)  M:278 (28.9), 16.2 (SD 1.2) | Valid and reliable survey items | MVPA | | 7.2 | |  |
| Ruíz-Roso 2020 (17) | Brazil | CS | Non-probabilistic, convenient sampling | Online survey | Before | Before confinement and lockdown | Apr 17-May 20, 2020 | | GP, M: 50 (43.5%)  F: 65 (56.5%), Age range:10-19 | IPAQ, Spanish | MVPA | | 59.1 | |  |
| Ruíz-Roso 2020 (17) | Chile | CS | Non-probabilistic, convenient sampling | Online survey | Before | Before confinement and lockdown | Apr 17-May 20, 2020 | | GP, M: 72 (42.4%)  F: 97 (57.1%), Age range:10-19 | IPAQ, Spanish | MVPA | | 20 | |  |
| Ruíz-Roso 2020 (17) | Colombia | CS | Non-probabilistic, convenient sampling | Online survey | Before | Before confinement and lockdown | Apr 17-May 20, 2020 | | GP, M: 69 (42.9%)  F: 91 (56.5%), Age range:10-19 | IPAQ, Spanish | MVPA | | 27.3 | |  |
| Ruíz-Roso 2020 (17) | Italy | CS | Non-probabilistic, convenient sampling | Online survey | Before | Before confinement and lockdown | Apr 17-May 20, 2020 | | GP, M: 38 (28.6%)  F: 93 (69.9%), Age range:10-19 | IPAQ, Spanish | MVPA | | 14.3 | |  |
| Ruíz-Roso 2020 (17) | Spain | CS | Non-probabilistic, convenient sampling | Online survey | Before | Before confinement and lockdown | Apr 17-May 20, 2020 | | GP, M: 60 (40.8%)  F: 87 (59.2%), Age range:10-19 | IPAQ, Spanish | MVPA | | 21.1 | |  |
| Ruíz-Roso 2020 (17) | Brazil | CS | Non-probabilistic, convenient sampling | Online survey | During | Confinement and lockdown | Apr 17-May 20, 2020 | | GP, M: 50 (43.5%)  F: 65 (56.5%), Age range:10-19 | IPAQ, Spanish | MVPA | | 7 | |  |
| Ruíz-Roso 2020 (17) | Chile | CS | Non-probabilistic, convenient sampling | Online survey | During | Confinement and lockdown | Apr 17-May 20, 2020 | | GP, M: 72 (42.4%)  F: 97 (57.1%), Age range:10-19 | IPAQ, Spanish | MVPA | | 9.4 | |  |
| Ruíz-Roso 2020 (17) | Colombia | CS | Non-probabilistic, convenient sampling | Online survey | During | Confinement and lockdown | Apr 17-May 20, 2020 | | GP, M: 69 (42.9%)  F: 91 (56.5%), Age range:10-19 | IPAQ, Spanish | MVPA | | 29.2 | |  |
| Ruíz-Roso 2020 (17) | Italy | CS | Non-probabilistic, convenient sampling | Online survey | During | Confinement and lockdown | Apr 17-May 20, 2020 | | GP, M: 38 (28.6%)  F: 93 (69.9%), Age range:10-19 | IPAQ, Spanish | MVPA | | 26.3 | |  |
| Ruíz-Roso 2020 (17) | Spain | CS | Non-probabilistic, convenient sampling | Online survey | During | Confinement and lockdown | Apr 17-May 20, 2020 | | GP, M: 60 (40.8%)  F: 87 (59.2%), Age range:10-19 | IPAQ, Spanish | MVPA | | 29.3 | |  |
| Schmidt, 2020 (18) | Germany | Longitudinal | Multi-stage sampling | Questionnaire | Pre | No restrictions | Aug 2018 to Apr 2020 | | GP, M: 859 (50.2%)  F: 852 (49.8%), Ages: 4-17 | MoMo PA questionnaire | MVPA | | 18.9 | |  |
| Schmidt, 2020 (18) | Germany | Longitudinal | Multi-stage sampling | Questionnaire | Pre | No restrictions | Aug 2018 to Apr 2020 | | GP, M: 859 (50.2%)  , Ages: 4-17 | MoMo PA questionnaire | MVPA | | 21.4 | |  |
| Schmidt, 2020 (18) | Germany | Longitudinal | Multi-stage sampling | Questionnaire | Pre | No restrictions | Aug 2018 to Apr 2020 | | GP, F: 852 (49.8%), Ages: 4-17 | MoMo PA questionnaire | MVPA | | 16.4 | |  |
| Schmidt, 2020 (18) | Germany | Longitudinal | Multi-stage sampling | Questionnaire | Pre | No restrictions | Aug 2018 to Apr 2020 | | GP, M& F, Ages: 4-5 | MoMo PA questionnaire | MVPA | | 62.2 | |  |
| Schmidt, 2020 (18) | Germany | Longitudinal | Multi-stage sampling | Questionnaire | Pre | No restrictions | Aug 2018 to Apr 2020 | | GP, M, Ages: 4-5 | MoMo PA questionnaire | MVPA | | 30 | |  |
| Schmidt, 2020 (18) | Germany | Longitudinal | Multi-stage sampling | Questionnaire | Pre | No restrictions | Aug 2018 to Apr 2020 | | GP, F, Ages: 4-5 | MoMo PA questionnaire | MVPA | | 32.2 | |  |
| Schmidt, 2020 (18) | Germany | Longitudinal | Multi-stage sampling | Questionnaire | Pre | No restrictions | Aug 2018 to Apr 2020 | | GP, M& F, Ages: 6-10 | MoMo PA questionnaire | MVPA | | 51.4 | |  |
| Schmidt, 2020 (18) | Germany | Longitudinal | Multi-stage sampling | Questionnaire | Pre | No restrictions | Aug 2018 to Apr 2020 | | GP, M, Ages: 6-10 | MoMo PA questionnaire | MVPA | | 28.7 | |  |
| Schmidt, 2020 (18) | Germany | Longitudinal | Multi-stage sampling | Questionnaire | Pre | No restrictions | Aug 2018 to Apr 2020 | | GP, F, Ages: 6-10 | MoMo PA questionnaire | MVPA | | 22.7 | |  |
| Schmidt, 2020 (18) | Germany | Longitudinal | Multi-stage sampling | Questionnaire | Pre | No restrictions | Aug 2018 to Apr 2020 | | GP, M &F, Ages: 11-13 | MoMo PA questionnaire | MVPA | | 22.1 | |  |
| Schmidt, 2020 (18) | Germany | Longitudinal | Multi-stage sampling | Questionnaire | Pre | No restrictions | Aug 2018 to Apr 2020 | | GP, M, Ages: 11-13 | MoMo PA questionnaire | MVPA | | 15.2 | |  |
| Schmidt, 2020 (18) | Germany | Longitudinal | Multi-stage sampling | Questionnaire | Pre | No restrictions | Aug 2018 to Apr 2020 | | GP, F, Ages: 11-13 | MoMo PA questionnaire | MVPA | | 6.9 | |  |
| Schmidt, 2020 (18) | Germany | Longitudinal | Multi-stage sampling | Questionnaire | Pre | No restrictions | Aug 2018 to Apr 2020 | | GP, M& F, Ages: 14-17 | MoMo PA questionnaire | MVPA | | 11.6 | |  |
| Schmidt, 2020 (18) | Germany | Longitudinal | Multi-stage sampling | Questionnaire | Pre | No restrictions | Aug 2018 to Apr 2020 | | GP, M, Ages: 14-17 | MoMo PA questionnaire | MVPA | | 5.7 | |  |
| Schmidt, 2020 (18) | Germany | Longitudinal | Multi-stage sampling | Questionnaire | Pre | No restrictions | Aug 2018 to Apr 2020 | | GP, F, Ages: 14-17 | MoMo PA questionnaire | MVPA | | 5.9 | |  |
| Schmidt, 2020 (18) | Germany | Longitudinal | Multi-stage sampling | Questionnaire | During | Restrictions | 20 Apr -1 May | | GP, M: 859 (50.2%)  F: 852 (49.8%), Age Range: 4-17 | MoMo PA questionnaire | MVPA | | 30.2 | |  |
| Schmidt, 2020 (18) | Germany | Longitudinal | Multi-stage sampling | Questionnaire | During | Restrictions | 20 Apr -1 May | | GP, M: 859, Ages: 4-17 | MoMo PA questionnaire | MVPA | | 34 | |  |
| Schmidt, 2020 (18) | Germany | Longitudinal | Multi-stage sampling | Questionnaire | During | Restrictions | 20 Apr -1 May | | GP, F: 852 (49.8%), Ages: 4-17 | MoMo PA questionnaire | MVPA | | 26.3 | |  |
| Schmidt, 2020 (18) | Germany | Longitudinal | Multi-stage sampling | Questionnaire | During | Restrictions | 20 Apr -1 May | | GP, M& F, Ages: 4-5 | MoMo PA questionnaire | MVPA | | 91.2 | |  |
| Schmidt, 2020 (18) | Germany | Longitudinal | Multi-stage sampling | Questionnaire | During | Restrictions | 20 Apr -1 May | | GP, M, Ages: 4-5 | MoMo PA questionnaire | MVPA | | 47.7 | |  |
| Schmidt, 2020 (18) | Germany | Longitudinal | Multi-stage sampling | Questionnaire | During | Restrictions | 20 Apr -1 May | | GP, F, Ages: 4-5 | MoMo PA questionnaire | MVPA | | 43.5 | |  |
| Schmidt, 2020 (18) | Germany | Longitudinal | Multi-stage sampling | Questionnaire | During | Restrictions | 20 Apr -1 May | | GP, M& F, Ages: 6-10 | MoMo PA questionnaire | MVPA | | 81.5 | |  |
| Schmidt, 2020 (18) | Germany | Longitudinal | Multi-stage sampling | Questionnaire | During | Restrictions | 20 Apr -1 May | | GP, M, Ages: 6-10 | MoMo PA questionnaire | MVPA | | 44.1 | |  |
| Schmidt, 2020 (18) | Germany | Longitudinal | Multi-stage sampling | Questionnaire | During | Restrictions | 20 Apr -1 May | | GP, F, Ages: 6-10 | MoMo PA questionnaire | MVPA | | 37.4 | |  |
| Schmidt, 2020 (18) | Germany | Longitudinal | Multi-stage sampling | Questionnaire | During | Restrictions | 20 Apr -1 May | | GP, M& F, Ages: 11-13 | MoMo PA questionnaire | MVPA | | 37.6 | |  |
| Schmidt, 2020 (18) | Germany | Longitudinal | Multi-stage sampling | Questionnaire | During | Restrictions | 20 Apr -1 May | | GP, M, Ages: 11-13 | MoMo PA questionnaire | MVPA | | 23.5 | |  |
| Schmidt, 2020 (18) | Germany | Longitudinal | Multi-stage sampling | Questionnaire | During | Restrictions | 20 Apr -1 May | | GP, F, Ages: 11-13 | MoMo PA questionnaire | MVPA | | 14.1 | |  |
| Schmidt, 2020 (18) | Germany | Longitudinal | Multi-stage sampling | Questionnaire | During | Restrictions | 20 Apr -1 May | | GP, M& F, Ages: 14-17 | MoMo PA questionnaire | MVPA | | 20.9 | |  |
| Schmidt, 2020 (18) | Germany | Longitudinal | Multi-stage sampling | Questionnaire | During | Restrictions | 20 Apr -1 May | | GP, M, Ages: 14-17 | MoMo PA questionnaire | MVPA | | 11.2 | |  |
| Schmidt, 2020 (18) | Germany | Longitudinal | Multi-stage sampling | Questionnaire | During | Restrictions | 20 Apr -1 May | | GP, F, Ages: 14-17 | MoMo PA questionnaire | MVPA | | 9.7 | |  |
| Xiang, 2020 (19) | China | CS | Probabilistic, random sampling | Community | Before | No restrictions | Jan 3-21, 2020 | | GP, M: 1242 (51.2%)  F: 1184 (48.8%), Age range: 6–17 | GPAQ | MVPA | | 60 | |  |
| Xiang, 2020 (19) | China | CS | Probabilistic, random sampling | Community | During | Public health emergency | Mar 13-23, 2020 | | GP, M: 1242 (51.2%)  F: 1184 (48.8%), Age range: 6–17 | GPAQ | MVPA | | 17.7 | |  |
| **Characteristics of studies with mixed population (adults & children)** | | | | | | | | | | | | | | | |
| **Reference** | **Country** | **Study design** | **Sampling method** | **Setting** | **Pre or during COVID-19** | **Restrictions during the COVID-19 Pandemic** | **Data collection time** | **Population description** | | **Physical activity instrument or used item** | | **Level of physical activity** | | **Prevalence of physical activity (%)** |  |
| de Matos, 2020 (20) | Brazil | CS | NR | Online Survey | Before | No | 12 Jun and 12th of Jul | GP, M: 217 (50.9%)  F: 209 (49.1%), Age Range: 7-80 | | IPAQ | | Moderate | | 48 |  |
| de Matos, 2020 (20) | Brazil | CS | NR | Online Survey | Before | No | 12 Jun and 12th of Ju | GP, M: 217 (50.9%)  F: 209 (49.1%),  Age range: 7-80 | | IPAQ | | High | | 48 |  |
| de Matos, 2020 (20) | Brazil | CS | NR | Online Survey | During | Restrictions: Social distancing | 12 Jun and 12th of Jul 2020 | GP, M: 217 (50.9%)  F: 209 (49.1%),  Age Range: 7-80 | | IPAQ | | Moderate | | 13 |  |
| de Matos, 2020 (20) | Brazil | CS | NR | Online Survey | During | Restrictions: Social distancing | 12 Jun and 12^th^ of Jul 2020 | GP, M: 217 (50.9%)  F: 209 (49.1%),  Age Range: 7-80 | | IPAQ | | High | | 3 |  |
| Zhou, 2021 (21) | China | CS | Snowball sampling | Online | Before | No restrictions: Recall information Jan 2020 | Jan 2020 | GP, M: 2427 (100%), Age range: 15-33 | | IPAQ | | MVPA | | 38.6 |  |
| Zhou, 2021 (21) | China | CS | Snowball sampling | Online | During | Restriction: lockdown, Feb 2020 | Feb 2020 | GP, M: 2427 (100%), Age range: 15-33 | | IPAQ | | MVPA | | 19.4 |  |
| Zhou, 2021 (21) | China | CS | Snowball sampling | Online | During | Restriction: 3 months **after lockdown** lifted, May 2020 | May 2020 | GP, M: 2427 (100%), Age range: 15-33 | | IPAQ | | MVPA | | 25.3 |  |
| Zhou, 2021(21) | China | CS | Snowball sampling | Online | Before | No restrictions: Recall information Jan 2020 | Jan 2020 | GP, F: 5688 (100%), Age range: 15-33 | | IPAQ | | MVPA | | 50.9 |  |
| Zhou, 2021 (21) | China | CS | Snowball sampling | Online | During | Restriction: lockdown, Feb 2020 | Feb 2020 | GP, F: 5688 (100%), Age range: 15-33 | | IPAQ | | MVPA | | 33.4 |  |
| Zhou, 2021 (21) | China | CS | Snowball sampling | Online | During | Restriction: 3 months **after** **lockdown lifted**, May 2020 | May 2020 | GP, F: 5688 (100%), Age range: 15-33 | | IPAQ | | MVPA | | 42.1 |  |

NR-Not reported; CS-Cross-sectional; LOS-Longitudinal Observational study; GP-General population; M-Male; F-Female; MVPA-Moderate to Vigorous Physical Activity; PAQ-Physical Activity Questionnaire; IPAQ- International Physical Activity Questionnaire; SF-IPAQ- Short Form International Physical Activity Questionnaire; MLTPAQ- Minnesota Leisure Time Physical Activity Questionnaire; GPAQ-Global Physical Activity Questionnaire

# **Table S5: Prevalence of Physical activity among adults during COVID-19**

| **References** | **Country** | **Study design** | **Sampling method** | **Setting** | **Pre or during COVID-19** | **Restrictions**  **during the COVID-19 Pandemic** | **Data collection time** | **Population**  **description** | **Physical activity instrument** | **Level of physical activity** | **Prevalence of physical activity (%)** |
| --- | --- | --- | --- | --- | --- | --- | --- | --- | --- | --- | --- |
| Aegerter, 2021 (22) | Switzerland | Longitudinal | NR | Community | Before | No restriction | Jan 2020 | GP, M: 54 (71.1%)  F: 22 (28.9%), Mean age (SD): 42.7  Range: 21.8 to 62.7 | IPAQ-SF, German version | High | 29 |
| Aegerter, 2021 (22) | Switzerland | Longitudinal | NR | Community | Before | No restriction | Jan 2020 | GP, M: 54 (71.1%)  F: 22 (28.9%), Mean age (SD): 42.7  Range: 21.8 to 62.8 | IPAQ-SF, German version | Moderate | 42 |
| Aegerter, 2021 (22) | Switzerland | Longitudinal | NR | Community | During | Fourth and fifth week of lockdown | Apr 2020 | GP, M: 54 (71.1%)  F: 22 (28.9%), Mean age (SD): 42.7  Range: 21.8 to 62.7 | IPAQ-SF, German version | High | 38 |
| Aegerter, 2021 (22) | Switzerland | Longitudinal | NR | Community | During | Fourth and fifth week of lockdown | Apr 2020 | GP, M: 54 (71.1%)  F: 22 (28.9%), Mean age (SD): 42.7  Range: 21.8 to 62.8 | IPAQ-SF, German version | Moderate | 37 |
| Alsalhe, 2020 (23) | Algeria, Libya, Saudi Arabia, & Tunisia | CS | Non-probabilistic, convenient sampling | Online survey | During | With restrictions | May 4-15, 2019 | GP, M: 222 (48.4%)  F: 237 (51.6%), Mean age (SD): 33.02 (8.46) | SF-IPAQ, Arabic | Moderate | 30.7 |
| Alsalhe, 2020 (23) | Algeria, Libya, Saudi Arabia, & Tunisia | CS | Non-probabilistic, convenient sampling | Online survey | During | With restrictions | May 4-15, 2020 | GP, M: 222 (48.4%)  F: 237 (51.6%), Mean age (SD): 33.02 (8.46) | SF-IPAQ, Arabic | High | 30.3 |
| Amini, 2020 (24) | Iran | CS | Non-probabilistic, convenient sampling | Online survey | Before | No restriction | May 4-15, 2020 | GP, M: 152 (22.6%)  F: 518 (77.4%), Mean age (SD): 29.24 (9.5) | SF-IPAQ | Moderate | 21.1 |
| Amini, 2020 (24) | Iran | CS | Non-probabilistic, convenient sampling | Online survey | Before | No restriction | May 4-15, 2020 | GP, M: 152 (22.6%)  F: 518 (77.4%), Mean age (SD): 29.24 (9.5) | SF-IPAQ | High | 28 |
| Amini, 2020 (24) | Iran | CS | Non-probabilistic, convenient sampling | Online survey | During | Social distancing was implementing. It means maintaining distance (approximately 2 m from others when possible), avoiding mass gatherings, remaining out of congregate settings, closure of gym, parks, pools, and so on | May 20-29, 2020 | GP, M: 152 (22.6%)  F: 518 (77.4%), Mean age (SD): 29.24 (9.5) | SF-IPAQ | Moderate | 13.4 |
| Amini, 2020 (24) | Iran | CS | Non-probabilistic, convenient sampling | Online survey | During | Social distancing was implementing. It means maintaining distance (approximately 2 m from others when possible), avoiding mass gatherings, remaining out of congregate settings, closure of gym, parks, pools, and so on | May 20-29, 2020 | GP, M: 152 (22.6%)  F: 518 (77.4%), Mean age (SD): 29.24 (9.5) | SF-IPAQ | High | 8.6 |
| Amini, 2020 (24) | Iran | CS | Non-probabilistic, convenient sampling | Online survey | Before | No restriction | Jan 20, 2020 | GP, F: 518 (100%) | SF-IPAQ | Moderate | 21.2 |
| Amini, 2020 (24) | Iran | CS | Non-probabilistic, convenient sampling | Online survey | Before | No restriction | Jan 20, 2020 | GP, F: 518 (100%) | SF-IPAQ | High | 28.6 |
| Amini, 2020 (24) | Iran | CS | Non-probabilistic, convenient sampling | Online survey | During | Social distancing was implementing. It means maintaining distance (approximately 2 m from others when possible), avoiding mass gatherings, remaining out of congregate settings, closure of gym, parks, pools, and so on | May 20-29, 2020 | GP, F: 518 (100%) | SF-IPAQ | Moderate | 13.5 |
| Amini, 2020 (24) | Iran | CS | Non-probabilistic, convenient sampling | Online survey | During | Social distancing was implementing. It means maintaining distance (approximately 2 m from others when possible), avoiding mass gatherings, remaining out of congregate settings, closure of gym, parks, pools, and so on | May 20-29, 2020 | GP, F: 518 (100%) | SF-IPAQ | High | 7.5 |
| Amini, 2020 (24) | Iran | CS | Non-probabilistic, convenient sampling | Online survey | Before | No restriction | Jan 20, 2020 | GP, M: 152 (100%) | SF-IPAQ | Moderate | 20.4 |
| Amini, 2020 (24) | Iran | CS | Non-probabilistic, convenient sampling | Online survey | Before | No restriction | Jan 20, 2020 | GP, M: 152 (100%) | SF-IPAQ | High | 26.3 |
| Amini, 2020 (24) | Iran | CS | Non-probabilistic, convenient sampling | Online survey | During | Social distancing was implementing. It means maintaining distance (approximately 2 m from others when possible), avoiding mass gatherings, remaining out of congregate settings, closure of gym, parks, pools, and so on | May 20-29, 2020 | GP, M: 152 (100%) | SF-IPAQ | Moderate | 12.7 |
| Amini, 2020 (24) | Iran | CS | Non-probabilistic, convenient sampling | Online survey | During | Social distancing was implementing. It means maintaining distance (approximately 2 m from others when possible), avoiding mass gatherings, remaining out of congregate settings, closure of gym, parks, pools, and so on | May 20-29, 2020 | GP, M: 152 (100%) | SF-IPAQ | High | 12.3 |
| Antunes, 2020 (25) | Portugal | CS | Non-probabilistic, convenient sampling | Online survey | During | State of emergency | Apr 1-15, 2020 | GP, M: 426 (30.3%)  F: 977 (69.6%), Mean (SD): 36.4 (11.7)  Range: 18-89 | SF-IPAQ | Moderate | 49.6 |
| Antunes, 2020 (25) | Portugal | CS | Non-probabilistic, convenient sampling | Online survey | During | State of emergency | Apr 1-15, 2020 | GP, M: 426 (30.3%)  F: 977 (69.6%), Mean (SD): 36.4 (11.7)  Range: 18-89 | SF-IPAQ | High | 18.5 |
| Boukrim, 2021 (26) | Morocco | CS | NR | Community | During | Mandatory home confinement | Apr 01 to Jun 10, 2020 | GP, M: 104 (25.6%)  F: 302 (74.4%), Mean age (SD):20.10 (1.36) | IPAQ-SF | Moderate | 30 |
| Bourdas, 2020 (27) | Greece | CS | Non-probabilistic, convenient sampling | Online survey | Before | No restriction | Mar-Apr, 2020 | GP, M:3255 (38.3%)  F: 5240 (61.7%), Mean age (95%CI): 37.2 (36.9-37.5) | Active-Q, Greek version | Moderate | 11.3 |
| Bourdas, 2020 (27) | Greece | CS | Non-probabilistic, convenient sampling | Online survey | Before | No restriction | Mar-Apr, 2020 | GP, M:3255 (38.3%)  F: 5240 (61.7%), Mean age (95%CI): 37.2 (36.9-37.5) | Active-Q, Greek version | High | 54.8 |
| Bourdas, 2020 (27) | Greece | CS | Non-probabilistic, convenient sampling | Online survey | During | Lockdown | Mar-Apr, 2020 | GP, M:3255 (38.3%)  F: 5240 (61.7%), Mean age (95%CI): 37.2 (36.9-37.5) | Active-Q, Greek version | Moderate | 9.9 |
| Bourdas, 2020 (27) | Greece | CS | Non-probabilistic, convenient sampling | Online survey | During | Lockdown | Mar-Apr, 2020 | GP, M:3255 (38.3%)  F: 5240 (61.7%), Mean age (95%CI): 37.2 (36.9-37.5) | Active-Q, Greek version | High | 47.6 |
| Bourdas, 2020 (27) | Greece | CS | Non-probabilistic, convenient sampling | Online survey | Before | No restriction | Mar-Apr, 2020 | GP, M:3255 (100%), Mean age (95%CI): 37.2 (36.9-37.5) | Active-Q, Greek version | Moderate | 9.5 |
| Bourdas, 2020 (27) | Greece | CS | Non-probabilistic, convenient sampling | Online survey | Before | No restriction | Mar-Apr, 2020 | GP, M:3255 (100%), Mean age (95%CI): 37.2 (36.9-37.5) | Active-Q, Greek version | High | 62.4 |
| Bourdas, 2020 (27) | Greece | CS | Non-probabilistic, convenient sampling | Online survey | Before | No restriction | Mar-Apr, 2020 | GP, M:3255 (100%), Mean age (95%CI): 37.2 (36.9-37.5) | Active-Q, Greek version | MVPA | 72 |
| Bourdas, 2020 (27) | Greece | CS | Non-probabilistic, convenient sampling | Online survey | During | Lockdown | Mar-Apr, 2020 | GP, M:3255 (100%), Mean age (95%CI): 37.2 (36.9-37.5) | Active-Q, Greek version | Moderate | 9.2 |
| Bourdas, 2020 (27) | Greece | CS | Non-probabilistic, convenient sampling | Online survey | During | Lockdown | Mar-Apr, 2020 | GP, M:3255 (100%), Mean age (95%CI): 37.2 (36.9-37.5) | Active-Q, Greek version | High | 49.9 |
| Bourdas, 2020 (27) | Greece | CS | Non-probabilistic, convenient sampling | Online survey | Before | No restriction | Mar-Apr, 2020 | GP, F: 5240 (100%), Mean age (95%CI): 37.2 (36.9-37.5) | Active-Q, Greek version | Moderate | 12.5 |
| Bourdas, 2020 (27) | Greece | CS | Non-probabilistic, convenient sampling | Online survey | Before | No restriction | Mar-Apr, 2020 | GP, F: 5240 (100%), Mean age (95%CI): 37.2 (36.9-37.5) | Active-Q, Greek version | High | 50 |
| Bourdas, 2020 (27) | Greece | CS | Non-probabilistic, convenient sampling | Online survey | During | Lockdown | Mar-Apr, 2020 | GP, F: 5240 (100%), Mean age (95%CI): 37.2 (36.9-37.5) | Active-Q, Greek version | Moderate | 10.3 |
| Bourdas, 2020 (27) | Greece | CS | Non-probabilistic, convenient sampling | Online survey | During | Lockdown | Mar-Apr, 2020 | GP, F: 5240 (100%), Mean age (95%CI): 37.2 (36.9-37.5) | Active-Q, Greek version | High | 46.3 |
| Carriedo, 2020 (28) | Spain | CS | Non-probabilistic, snowball sampling | Online survey | During | Home isolated | NR | GP, M: 236 (49.3%)  F:243 (50.7%), Mean age (SD): 65.49 (5.14)  Age range: 60-92 | SF-IPAQ, Spanish | MVPA | 39.9 |
| Carriedo, 2020 (28) | Spain | CS | Non-probabilistic, snowball sampling | Online survey | During | Home isolated | NR | GP, M: 236 (100%), Mean age (SD): 66.03 (5.54) | SF-IPAQ, Spanish | MVPA | 41.1 |
| Carriedo, 2020 (28) | Spain | CS | Non-probabilistic, snowball sampling | Online survey | During | Home isolated | NR | GP, F: 243 (100%), Mean age (SD): 64.98 (4.67) | SF-IPAQ, Spanish | MVPA | 38.7 |
| Chopra, 2020 (29) | India | CS | Non-probabilistic, quota sampling | Online survey | Before | No restriction | Aug 15-30, 2020 | GP, M: 583 (58.5)  F: 429 (43.1%), Mean age (SD): 33.33 (14.5) | Electronic survey questionnaire | MVPA | 31 |
| Chopra, 2020 (29) | India | CS | Non-probabilistic, quota sampling | Online survey | During | During the unlock phase | Aug 15-30, 2020 | GP, M: 583 (58.5)  F: 429 (43.1%), Mean age (SD): 33.33 (14.5) | Electronic survey questionnaire | MVPA | 30.7 |
| Constantini, 2021 (8) | Israel | CS | Non-probability (self-selection online survey) | Community | During | With restrictions, lockdown | Apr 10 to 5 May, 2020 | GP, M: 231 (69.4%)  F: 102 (30.6%), more than 17 Y | The Health Behaviours in School-aged Children survey (HBSC) | MVPA | 83.1 |
| Crochemore-Silva, 2020 (30) | Brazil | CS | Probabilistic, cluster sampling | Community | During | Period of social detachment | May 7-9, 2020 | GP, M: 37.1%  F: 62.9%, Age range: >18 | IPAQ | MVPA | 7.7 |
| Crochemore-Silva, 2020 (30) | Brazil | CS | Probabilistic, cluster sampling | Community | During | Period of social detachment | May 7-9, 2020 | GP, M: 37.1%, Age range: >18 | IPAQ | MVPA | 12.9 |
| Crochemore-Silva, 2020 (30) | Brazil | CS | Probabilistic, cluster sampling | Community | During | Period of social detachment | May 7-9, 2020 | GP, F: 62.9%, Age range: >18 | IPAQ | MVPA | 4.6 |
| Di Sebastiano, 2020 (31) | Canada | CS | Non-probabilistic, convenient sampling | Community | Before | No restriction | 10 to 16 Feb 2020 | GP, M: 229 (9.8%)  F: 2109 (90.2%), Age range: >18 | PAC app developed by Participation | MVPA | 43.9 |
| Faulkner, 2020 (32) | UK | CS | Non-probabilistic, convenient sampling | Online survey | Before | No restriction | Apr-May, 2020 | GP, M:1024 (32.8%)  F: 2094 (67.1%)  unk: 3 (0.1%), Mean age (SD): 43.9 (14.9) | SF-IPAQ | MVPA | 73.3 |
| Faulkner, 2020 (32) | UK | CS | Non-probabilistic, convenient sampling | Online survey | Before | No restriction | Apr-May, 2020 | GP, M: 1024 (100%), | SF-IPAQ | MVPA | 79.7 |
| Faulkner, 2020 (32) | UK | CS | Non-probabilistic, convenient sampling | Online survey | Before | No restriction | Apr-May, 2020 | GP, F: 2094 (100%), | SF-IPAQ | MVPA | 70.2 |
| Faulkner, 2020 (32) | UK | CS | Non-probabilistic, convenient sampling | Online survey | During | Physical distancing(>2m), self-isolation for >70y & people with pre-existing health conditions, or COVID-19 symptoms or diagnosis, only one form of exercise/day, only shopping for basic necessities (e.g., food/medicine), travel for specific medical needs, essential travel for key workers, everyone else works from home; pubs/gyms/playgrounds/cinemas/restaurants/places of worship closed | Apr-May, 2020 | GP, M:1024 (32.8%)  F: 2094 (67.1%)  unk: 3 (0.1%), Mean age (SD): 43.9 (14.9) | SF-IPAQ | Moderate | 52.8 |
| Faulkner, 2020 (32) | UK | CS | Non-probabilistic, convenient sampling | Online survey | During | Physical distancing(>2m), self-isolation for >70y & people with pre-existing health conditions, or COVID-19 symptoms or diagnosis, only one form of exercise/day, only shopping for necessities (e.g., food/medicine), travel for specific medical needs, essential travel for key workers, everyone else works from home; pubs/gyms/playgrounds/cinemas/restaurants/places of worship closed | Apr-May, 2020 | GP, M:1024 (32.8%)  F: 2094 (67.1%)  unk: 3 (0.1%), Mean age (SD): 43.9 (14.9) | SF-IPAQ | High | 38.1 |
| Faulkner, 2020 (32) | Ireland | CS | Non-probabilistic, convenient sampling | Online survey | Before | No restriction | Apr-May, 2020 | GP, M: 232 (25.7%)  F: 668 (74.0%)  unk: 3 (0.3%), Mean age (SD): 38.7 (13.3) | SF-IPAQ | MVPA | 69.7 |
| Faulkner, 2020 (32) | Ireland | CS | Non-probabilistic, convenient sampling | Online survey | Before | No restriction | Apr-May, 2020 | GP, M: (100%) | SF-IPAQ | MVPA | 78.4 |
| Faulkner, 2020 (32) | Ireland | CS | Non-probabilistic, convenient sampling | Online survey | Before | No restriction | Apr-May, 2020 | GP, F: (100%) | SF-IPAQ | MVPA | 66.7 |
| Faulkner, 2020 (32) | Ireland | CS | Non-probabilistic, convenient sampling | Online survey | During | Physical distancing(>2m), self-isolation for >70y & people with pre-existing health conditions, or COVID-19 symptoms or diagnosis, only one form of exercise/day, only shopping for basic necessities (e.g., food/medicine), travel for specific medical needs, essential travel for key workers, everyone else works from home; pubs/gyms/playgrounds/cinemas/restaurants/places of worship closed | Apr-May, 2020 | GP, M: 232 (25.7%)  F: 668 (74.0%)  unk: 3 (0.3%), Mean age (SD): 38.7 (13.3) | SF-IPAQ | Moderate | 55 |
| Faulkner, 2020 (32) | Ireland | CS | Non-probabilistic, convenient sampling | Online survey | During | Physical distancing(>2m), self-isolation for >70y & people with pre-existing health conditions, or COVID-19 symptoms or diagnosis, only one form of exercise/day, only shopping for basic necessities (e.g., food/medicine), travel for specific medical needs, essential travel for key workers, everyone else works from home; pubs/gyms/playgrounds/cinemas/restaurants/places of worship closed | Apr-May, 2020 | GP, M: 232 (25.7%)  F: 668 (74.0%)  unk: 3 (0.3%), Mean age (SD): 38.7 (13.3) | SF-IPAQ | High | 36.1 |
| Faulkner, 2020 (32) | New Zealand | CS | Non-probabilistic, convenient sampling | Online survey | Before | No restriction | Apr-May, 2020 | GP, M: 1087 (27.1%)  F: 2886 (72.0%)  unk: 24 (0.9%), Mean age (SD): 46.5 (14.7) | SF-IPAQ | MVPA | 78.2 |
| Faulkner, 2020 (32) | New Zealand | CS | Non-probabilistic, convenient sampling | Online survey | Before | No restriction | Apr-May, 2020 | GP, M: (100%) | SF-IPAQ | MVPA | 82.9 |
| Faulkner, 2020 (32) | New Zealand | CS | Non-probabilistic, convenient sampling | Online survey | Before | No restriction | Apr-May, 2020 | GP, F: (100%) | SF-IPAQ | MVPA | 76.5 |
| Faulkner, 2020 (32) | New Zealand | CS | Non-probabilistic, convenient sampling | Online survey | During | Physical distancing(>2m), self-isolation for >70y & people with pre-existing health conditions, or COVID-19 symptoms or diagnosis, only one form of exercise/day, only shopping for basic necessities (e.g., food/medicine), travel for specific medical needs, essential travel for key workers, everyone else works from home; pubs/gyms/playgrounds/cinemas/restaurants/places of worship closed | Apr-May, 2020 | GP, M: 1087 (27.1%)  F: 2886 (72.0%)  unk: 24 (0.9%), Mean age (SD): 46.5 (14.7) | SF-IPAQ | Moderate | 54.4 |
| Faulkner, 2020 (32) | New Zealand | CS | Non-probabilistic, convenient sampling | Online survey | During | Physical distancing(>2m), self-isolation for >70y & people with pre-existing health conditions, or COVID-19 symptoms or diagnosis, only one form of exercise/day, only shopping for basic necessities (e.g., food/medicine), travel for specific medical needs, essential travel for key workers, everyone else works from home; pubs/gyms/playgrounds/cinemas/restaurants/places of worship closed | Apr-May, 2020 | GP, M: 1087 (27.1%)  F: 2886 (72.0%)  unk: 24 (0.9%), Mean age (SD): 46.5 (14.7) | SF-IPAQ | High | 38 |
| Faulkner, 2020 (32) | Australia | CS | Non-probabilistic, convenient sampling | Online survey | Before | No restriction | Apr-May, 2020 | GP, M: 89 (22.6%)  F: 304 (77.2%)  unk: 1 (0.3%), Mean age (SD): 41.1 (14.0) | SF-IPAQ | MVPA | 74.4 |
| Faulkner, 2020 (32) | Australia | CS | Non-probabilistic, convenient sampling | Online survey | Before | No restriction | Apr-May, 2020 | GP, M: 89 (100%) | SF-IPAQ | MVPA | 80.9 |
| Faulkner, 2020 (32) | Australia | CS | Non-probabilistic, convenient sampling | Online survey | Before | No restriction | Apr-May, 2020 | GP, F: 304(100%) | SF-IPAQ | MVPA | 72.7 |
| Faulkner, 2020 (32) | Australia | CS | Non-probabilistic, convenient sampling | Online survey | During | Physical distancing(>2m), self-isolation for >70y & people with pre-existing health conditions, or COVID-19 symptoms or diagnosis, only one form of exercise/day, only shopping for basic necessities (e.g., food/medicine), travel for specific medical needs, essential travel for key workers, everyone else works from home; pubs/gyms/playgrounds/cinemas/restaurants/places of worship closed | Apr-May, 2020 | GP, M: 89 (22.6%)  F: 304 (77.2%)  unk: 1 (0.3%), Mean age (SD): 46.5 (14.7) | SF-IPAQ | Moderate | 49.1 |
| Faulkner, 2020 (32) | Australia | CS | Non-probabilistic, convenient sampling | Online survey | During | Physical distancing(>2m), self-isolation for >70y & people with pre-existing health conditions, or COVID-19 symptoms or diagnosis, only one form of exercise/day, only shopping for basic necessities (e.g., food/medicine), travel for specific medical needs, essential travel for key workers, everyone else works from home; pubs/gyms/playgrounds/cinemas/restaurants/places of worship closed | Apr-May, 2020 | GP, M: 89 (22.6%)  F: 304 (77.2%)  unk: 1 (0.3%), Mean age (SD): 46.5 (14.7) | SF-IPAQ | High | 41 |
| Franco, 2020 (33) | Spain | Longitudinal | NR | Online Survey | Before | No restriction | Oct | GP, M:149 (50.2%)  F:148(49.8%), M=42.76  SD=7.79 | IPAQ, | MVPA | 50.2 |
| Franco, 2020 (33) | Spain | Longitudinal | NR | Online Survey | Before | No restriction | Oct | GP, M:149, M=42.76  SD=7.79 | IPAQ, | MVPA | 59.7 |
| Franco, 2020 (33) | Spain | Longitudinal | NR | Online Survey | Before | No restriction | Oct | GP, F:148, M=42.76  SD=7.79 | IPAQ, | MVPA | 40.5 |
| Franco, 2020 (33) | Spain | Longitudinal | NR | Online Survey | During | During confinement due to COVID19 restrictions | May 2020 | GP, M:149 (50.2%)  F:148(49.8%), Mean age (SD)= 42.76  (7.79) | IPAQ, | MVPA | 51.9 |
| Franco, 2020 (33) | Spain | Longitudinal | NR | Online Survey | During | During confinement due to COVID19 restrictions | May 2020 | GP, M:149, Mean age (SD)= 42.76(7.79) | IPAQ, | MVPA | 53.7 |
| Franco, 2020 (33) | Spain | Longitudinal | NR | Online Survey | During | During confinement due to COVID19 restrictions | May 2020 | GP, F:148, M=42.76  SD=7.79 | IPAQ, | MVPA | 46.6 |
| Franco, 2020 (33) | Spain | Longitudinal | NR | Online Survey | Before | No restriction | Oct | GP, M=138(49.3%)  F=142 (50.7%), M=42.76  SD=7.79 | IPAQ, | MVPA | *49.6 |
| Franco, 2020 (33) | Spain | Longitudinal | NR | Online Survey | After | During confinement due to COVID19 restrictions | May 2020 | GP, M=138(49.3%)  F=142 (50.7%), M=42.76  SD=7.79 | IPAQ, | MVPA | *49.3 |
| Fuentes-García, 2020 (34) | 29 different countries of Asia, America, Africa, and Europe | CS | Non-probabilistic, convenient sampling | Community | Before | No restriction | Mar 3-Apr 14, 2020 | GP, Mean age (SD): 38.31 (13.70) | No | MVPA | 63.3 |
| Fuentes-García, 2020 (34) | 29 different countries of Asia, America, Africa, and Europe | CS | Non-probabilistic, convenient sampling | Community | During | Confinement | Mar 3-Apr 14, 2020 | GP, Mean age (SD): 38.31 (13.70) | No | MVPA | 41.6 |
| Gallè, 2020 (35) | Italy | CS | Non-probabilistic, convenient sampling | Online survey | During | Lockdown | Apr 20, 2020 | GP, M: 494 (34.5%)  F: 936 (65.5%), Mean age (SD): 22.9 (3.5) | IPAQ | MVPA | 44.7 |
| Giustino, 2020 (36) | Italy | CS | Snowball sampling | Online survey | Before | No restrictions | Mar 30 - Apr 2, 2020 | GP, M: 391 (49%)  F: 411 (51%), Mean age (SD): 32.27 (12.81) | SF-IPAQ | Moderate | 44 |
| Giustino, 2020 (36) | Italy | CS | Snowball sampling | Online survey | Before | No restrictions | Mar 30 - Apr 2, 2020 | GP, M: 391 (49%)  F: 411 (51%), Mean age (SD): 32.27 (12.81) | SF-IPAQ | High | 50 |
| Giustino, 2020 (36) | Italy | CS | Snowball sampling | Online survey | During | Restrictions | Mar 30 - Apr 2, 2020 | GP, M: 391 (49%)  F: 411 (51%), Mean age (SD): 32.27 (12.81) | SF-IPAQ | Moderate | 51 |
| Giustino, 2020 (36) | Italy | CS | Snowball sampling | Online survey | During | Restrictions | Mar 30 – Apr 2, 2020 | GP, M: 391 (49%)  F: 411 (51%), Mean age (SD): 32.27 (12.81) | SF-IPAQ | High | 24 |
| Hall-López, 2020 (37) | Mexico | CS | Non-probability | Online survey | Before | No restrictions | NR | GP, M: 22 (59%)  F: 15 (41%), Mean age (SD): 27.8 (6.1) | IPAQ | Moderate | 37.8 |
| Hall-López, 2020 (37) | Mexico | CS | Non-probability | Online survey | Before | No restrictions | NR | GP, M: 22 (59%)  F: 15 (41%), Mean age (SD): 27.8 (6.1) | IPAQ | High | 37 |
| Hall-López, 2020 (37) | Mexico | CS | Non-probability | Online survey | During | Restrictions | NR | GP, M: 22 (59%)  F: 15 (41%), Mean age (SD): 27.8 (6.1) | IPAQ | Moderate | 25.5 |
| Hall-López, 2020 (37) | Mexico | CS | Non-probability | Online survey | During | Restrictions | NR | GP, M: 22 (59%)  F: 15 (41%), Mean age (SD): 27.8 (6.1) | IPAQ | High | 24.7 |
| Hu, 2020 (38) | China | CS | Non-probabilistic, convenient sampling | Online survey | During | 4 months Immediately after the COVID-19 outbreak announced | May 10-15, 2020 | GP, M:535 (51.8%)  F: 498 (48.2%), Age range: 18-60 (18 and 30 years: 61.7%) | SF-IPAQ | MVPA | 58.8 |
| Jacob, 2020 (39) | UK | CS | Non-probabilistic, convenient sampling | Online survey | During | Social distancing (i.e., following UK government enforced restrictions that limited movement of people) | Mar 17, 2020 | GP, M: 36.2%  F: 63.8%, Age range: >18 (35-64:50.1%) | No | MVPA | 63.4 |
| Jacob, 2020 (39) | UK | CS | Non-probabilistic, convenient sampling | Online survey | During | Social distancing (i.e., following UK government enforced restrictions that limited movement of people) | Mar 17, 2020 | GP, M: 100%, Age range: >18 (35-64:50.1%) | No | MVPA | 61.3 |
| Jacob, 2020 (39) | UK | CS | Non-probabilistic, convenient sampling | Online survey | During | Social distancing (i.e., following UK government enforced restrictions that limited movement of people) | Mar 17, 2020 | GP, F: 100%, Age range: >18 (35-64:50.1%) | No | MVPA | 64.6 |
| Knell, 2020 (40) | US | CS | Non-probabilistic, convenient sampling | Online survey | During | “Stay-at-Home” orders | Apr 15-May 5, 2020 | GP, M: 589 32.6%  F: 1220 67.4%, Age range: >18 (35-49:39.8%) | SF-IPAQ | High | 45.4 |
| Knell, 2020 (40) | US | CS | Non-probabilistic, convenient sampling | Online survey | During | “Stay-at-Home” orders | Apr 15-May 5, 2020 | GP, M: 589 32.6%  F: 1220 67.4%, Age range: >18 (35-49:39.8%) | SF-IPAQ | Moderate | 30.6 |
| López-Bueno, 2020 (41) | Spain | CS | Non-probabilistic, convenient sampling | Online survey | During | From the seventh day of national confinement in Spain being enacted). | Mar 22–Apr 5, 2020 | GP, M: 1320 (48.2%)  F: 1421 (51.8%), Mean age (SD): 34.2 (13.0)  Age range: >18 | Physical Activity Vital Sign (PAVS) short version, | MVPA | 55.5 |
| López-Bueno, 2020 (41) | Spain | CS | Non-probabilistic, convenient sampling | Online survey | Before | No restriction | Mar 22–Apr 5, 2020 | GP, M: 1320 (48.2%)  F: 1421 (51.8%), Mean age (SD): 34.2 (13.0)  Age range: >18 | Physical Activity Vital Sign (PAVS) short version, | MVPA | 35.1 |
| Lesser, 2020 (41) | Canada |  | Non-probabilistic, snowball sampling | Online survey | During | Strictest public health restrictions in Canada (nationwide restrictions were in place for 50 days) | Apr-early May, 2020 | GP, M: 215 (19.6%)  F: (53.6%), Mean age (SD): 42 (15)  Age range: >19 | Godin questionnaire | MVPA | 36.6 |
| López-Bueno, 2020 (41) | Spain | CS | Convenience | Online survey | Before | No restrictions | Mar 2-29, 2020 | GP, M: 938 (45.9%)  F: 1104 (54.1%), Mean age (SD): 35.9 (13.6) | Physical Activity vital Sign- SF (PAVS) | MVPA | 60.6 |
| López-Bueno, 2020 (41) | Spain | CS | Convenience | Online survey | Before | No restrictions | Mar 2-29, 2020 | GP, M: 938 (45.9%) | Physical Activity vital Sign- SF (PAVS) | MVPA | 70.6 |
| López-Bueno, 2020 (41) | Spain | CS | Convenience | Online survey | Before | No restrictions | Mar 2-29, 2020 | GP, F: 1104 (54.1) | Physical Activity vital Sign- SF (PAVS) | MVPA | 52.2 |
| López-Bueno, 2020 (41) | Spain | CS | Convenience | Online survey | During | Restrictions | Mar 2-29, 2020 | GP, M: 938 (45.9%)  F: 1104 (54.1%), Mean age (SD): 35.9 (13.6) | Physical Activity vital Sign- SF (PAVS) | MVPA | 48.9 |
| López-Bueno, 2020 (41) | Spain | CS | Convenience | Online survey | During | Restrictions | Mar 2-29, 2020 | GP, M: 938 (45.9%) | Physical Activity vital Sign- SF (PAVS) | MVPA | 53.5 |
| López-Bueno, 2020 (41) | Spain | CS | Convenience | Online survey | During | Restrictions | Mar 2-29, 2020 | GP, F: 1104 (54.1) | Physical Activity vital Sign- SF (PAVS) | MVPA | 45 |
| Malta, 2020 (42) | Brazil | CS | Non-probabilistic, chain sampling | Online survey | Before | No restriction | Apr 24-May 24, 2020. | GP, M: 20955 (46.4%)  F: 24206 (53.6%), Age range: >18 (24.7% 18-29) | Online questionnaire | MVPA | 30.1 |
| Malta, 2020 (42) | Brazil | CS | Non-probabilistic, chain sampling | Online survey | Before | No restriction | Apr 24-May 24, 2020. | GP, M: 20955 (100%)  , Age range: >18 (24.7% 18-29) | Online questionnaire | MVPA | 33 |
| Malta, 2020 (42) | Brazil | CS | Non-probabilistic, chain sampling | Online survey | Before | No restriction | Apr 24-May 24, 2020. | GP, F: 24206 (100%), Age range: >18 (24.7% 18-29) | Online questionnaire | MVPA | 27.6 |
| Malta, 2020 (42) | Brazil | CS | Non-probabilistic, chain sampling | Online survey | During | Social restriction intended to prevent and control the COVID-19 pandemic | Apr 24-May 24, 2020. | GP, M: 20955 (46.4%)  F: 24206 (53.6%), Age range: >18 (24.7% 18-29) | Online questionnaire | MVPA | 12 |
| Malta, 2020 (42) | Brazil | CS | Non-probabilistic, chain sampling | Online survey | During | Social restriction intended to prevent and control the COVID-19 pandemic | Apr 24-May 24, 2020. | GP, M: 20955 (100%), Age range: >18 (24.7% 18-29) | Online questionnaire | MVPA | 14 |
| Malta, 2020 (42) | Brazil | CS | Non-probabilistic, chain sampling | Online survey | During | Social restriction intended to prevent and control the COVID-19 pandemic | Apr 24-May 24, 2020. | GP, F: 24206 (100%), Age range: >18 (24.7% 18-29) | Online questionnaire | MVPA | 10.3 |
| Martínez-de-Quel, 2020 (43) | Spain | LOS (Longitudinal observation study) | Non-probabilistic, convenient sampling | Online survey | Before | No restriction | Mar 16-31, 2020 | GP, M: 101 (63%)  F: 60 (37%), Mean age (SD): 35.0 (11.2)  Age range: 19–65 | Minnesota Leisure Time PA Questionnaire (MLTPAQ), Spanish | MVPA | 85.7 |
| Martínez-de-Quel, 2020 (43) | Spain | LOS (Longitudinal observation study) | Non-probabilistic, convenient sampling | Online survey | During | Lockdown, State of emergency | Apr 30-May 11, 2020 | GP, M: 101 (63%)  F: 60 (37%), Mean age (SD): 35.0 (11.2)  Age range: 19–65 | Minnesota Leisure Time PA Questionnaire (MLTPAQ), Spanish | MVPA | 73.3 |
| Maugeri, 2020 (44) | Italy | CS | Non-probabilistic, convenient sampling | Online survey | Before | No restriction | Apr 1-30, 2020 | GP, M: 1098 (43.6%)  F: 1426 (56.4%), young aged <21 (n=346),  young adult aged 21–40 (n=1178),  adults aged 41–60 (n=704),  over 60 years (n=296) | SF-IPAQ | Moderate | 35.18 |
| Maugeri, 2020 (44) | Italy | CS | Non-probabilistic, convenient sampling | Online survey | Before | No restriction | Apr 1-30, 2020 | GP, M: 1098 (43.6%)  F: 1426 (56.4%), young aged <21 (n=346),  young adult aged 21–40 (n=1178),  adults aged 41–60 (n=704),  over 60 years (n=296) | SF-IPAQ | High | 41.76 |
| Maugeri, 2020 (44) | Italy | CS | Non-probabilistic, convenient sampling | Online survey | During | COVID-19 emergency, self-quarantine | Apr 1-30, 2020 | GP, M: 1098 (43.6%)  F: 1426 (56.4%), young aged <21 (n=346),  young adult aged 21–40 (n=1178),  adults aged 41–60 (n=704),  over 60 years (n=296) | SF-IPAQ | Moderate | 29.75 |
| Maugeri, 2020 (44) | Italy | CS | Non-probabilistic, convenient sampling | Online survey | During | COVID-19 emergency, self-quarantine | Apr 1-30, 2020 | GP, M: 1098 (43.6%)  F: 1426 (56.4%), young aged <21 (n=346),  young adult aged 21–40 (n=1178),  adults aged 41–60 (n=704),  over 60 years (n=296) | SF-IPAQ | High | 30.63 |
| Meza, 2021(45) | Mexico | CS | Non-probability | Questionnaire | Before | No restrictions | NR | GP, M: 17 (53.1%)  F: 15 (46.9%), Mean age: 21.4 ± 3.6 | IPAQ | Moderate | 5.2 |
| Meza, 2021(45) | Mexico | CS | Non-probability | Questionnaire | Before | No restrictions | NR | GP, M: 17 (53.1%)  F: 15 (46.9%), Mean age: 21.4 ± 3.6 | IPAQ | High | 91.2 |
| Meza, 2021(45) | Mexico | CS | Non-probability | Questionnaire | During | Confinement | NR | GP, M: 17 (53.1%)  F: 15 (46.9%), Mean age: 21.4 ± 3.6 | IPAQ | Moderate | 6.8 |
| Meza, 2021(45) | Mexico | CS | Non-probability | Questionnaire | During | Confinement | NR | GP, M: 17 (53.1%)  F: 15 (46.9%), Mean age: 21.4 ± 3.6 | IPAQ | High | 82.3 |
| Newby, 2020 (46) | Australia | CS | Non-probabilistic, convenient sampling | Online survey | During | Initial COVID-19 outbreak (and enforcement of social distancing laws) | 27 Mar-Apr 7, 2020 | GP, M: 656 (12.9%)  F: 4348 (86%)  Un Known: 1.3%, Age range: 18-Over 75 | Physical Activity Vital Sign | MVPA | 42.7 |
| Orlandi, 2021 (47) | Italy | CS | Snowball sampling technique | Online survey | Before | No restrictions | May 2020 | GP, M: 761 (34.3%), F: 1457 (65.7), M: 37.9 +/- 15.0 years and F: 38.4 +/- 14.9 years; age range 18-70 years | IPAQ | MVPA | 56.1 |
| Orlandi, 2021 (47) | Italy | CS | Snowball sampling technique | Online survey | During lockdown | Fifth week of the home confinement | May 2020 | GP, M: 761 (34.3%), F: 1457 (65.7), M: 37.9 +/- 15.0 years and F: 38.4 +/- 14.9 years; age range 18-70 years | IPAQ | MVPA | 52.1 |
| Ozdemir, 2020 (48) | Turkey | CS | Non-probabilistic, virtual snowball sampling | Online survey | During | Eight weeks after the first case of COVID-19, quarantine, isolation and social isolation in the scope of combating the COVID-19 outbreak | May 2020 | GP, M: 895 (38.9%)  F: 1406 (61.1%), Mean age (SD): 35.0 (10.5) for females and 38.2 (11.3) males  Age range: 20-75 | IPAQ, Turkish | MVPA | 6.9 |
| Parker, 2021 (16) | Australia | Longitudinal | Snowball sampling technique | Online survey | During | Stay-at-home period | Apr or May | GP, F: 980 (82.5)  M:208 (17.5), 37.4 (15.1) | Valid and reliable survey items | MVPA | 33 |
| Qi, 2020 (49) | China | CS | Non-probabilistic, convenient sampling | Online survey | During | Home confinement restrictions | Feb 25-Mar 15, 2020 | GP, M: 250 (38.8%)  F: 395 (61.2%), Mean age (SD): 31.8 (8.6)  Age range: 18-66 | SF-IPAQ, Chinese | Moderate | 18 |
| Qi, 2020 (49) | China | CS | Non-probabilistic, convenient sampling | Online survey | During | Home confinement restrictions | Feb 25-Mar 15, 2020 | GP, M: 250 (38.8%)  F: 395 (61.2%), Mean age (SD): 31.8 (8.6)  Age range: 18-66 | SF-IPAQ, Chinese | High | 17.2 |
| Qi, 2020 (49) | China | CS | Non-probabilistic, convenient sampling | Online survey | Before | No restriction | Feb 25-Mar 15, 2020 | GP, M: 250 (38.8%)  F: 395 (61.2%), Mean age (SD): 31.8 (8.6)  Age range: 18-66 | SF-IPAQ, Chinese | Moderate | 49.3 |
| Qi, 2020 (49) | China | CS | Non-probabilistic, convenient sampling | Online survey | Before | No restriction | Feb 25-Mar 15, 2020 | GP, M: 250 (38.8%)  F: 395 (61.2%), Mean age (SD): 31.8 (8.6)  Age range: 18-66 | SF-IPAQ, Chinese | High | 26.4 |
| Qin, 2020 (50) | China | CS | Non-probabilistic, snowball and quotas sampling | Online survey | During | A series of prevention and control strategies, such as locking down entire cities, and travel warnings, home-based medical observation, and home quarantine. Furthermore, national holidays were extended, and family reunions discouraged | Jan 24-Feb 2, 2020 | GP, M: 5633 (46.5%)  F: 6474 (53.5%), Age range 18-80 | SF-IPAQ, Chinese and Tibetan | MVPA | 42.5 |
| Qin, 2020 (50) | China | CS | Non-probabilistic, snowball and quotas sampling | Online survey | During | A series of prevention and control strategies, such as locking down entire cities, and travel warnings, home-based medical observation, and home quarantine. Furthermore, national holidays were extended, and family reunions discouraged | Jan 24-Feb 2, 2020 | GP, M: 5633 (100%), Age range 18-80 | SF-IPAQ, Chinese and Tibetan | Vigorous | 23 |
| Qin, 2020 (50) | China | CS | Non-probabilistic, snowball and quotas sampling | Online survey | During | A series of prevention and control strategies, such as locking down entire cities, and travel warnings, home-based medical observation, and home quarantine. Furthermore, national holidays were extended, and family reunions discouraged | Jan 24-Feb 2, 2020 | GP, F: 6474 (53.5%), Age range 18-80 | SF-IPAQ, Chinese and Tibetan | Vigorous | 19.4 |
| Qin, 2020 (50) | China | CS | Non-probabilistic, snowball and quotas sampling | Online survey | During | A series of prevention and control strategies, such as locking down entire cities, and travel warnings, home-based medical observation, and home quarantine. Furthermore, national holidays were extended, and family reunions discouraged | Jan 24-Feb 2, 2020 | GP, M: 5633 (100%), Age range 18-80 | SF-IPAQ, Chinese and Tibetan | Moderate | 21.9 |
| Qin, 2020 (50) | China | CS | Non-probabilistic, snowball and quotas sampling | Online survey | During | A series of prevention and control strategies, such as locking down entire cities, and travel warnings, home-based medical observation, and home quarantine. Furthermore, national holidays were extended, and family reunions discouraged | Jan 24-Feb 2, 2020 | GP, F: 6474 (53.5%), Age range 18-80 | SF-IPAQ, Chinese and Tibetan | Moderate | 21.2 |
| Qin, 2020 (50) | China | CS | Non-probabilistic, snowball and quotas sampling | Online survey | During | A series of prevention and control strategies, such as locking down entire cities, and travel warnings, home-based medical observation, and home quarantine. Furthermore, national holidays were extended, and family reunions discouraged | Jan 24-Feb 2, 2020 | GP, M: 5633 (100%), Age range 18-80 | SF-IPAQ, Chinese and Tibetan | MVPA | 44.9 |
| Qin, 2020 (50) | China | CS | Non-probabilistic, snowball and quotas sampling | Online survey | During | A series of prevention and control strategies, such as locking down entire cities, and travel warnings, home-based medical observation, and home quarantine. Furthermore, national holidays were extended, and family reunions discouraged | Jan 24-Feb 2, 2020 | GP, F: 6474 (53.5%), Age range 18-80 | SF-IPAQ, Chinese and Tibetan | MVPA | 40.6 |
| Rahman, 2020 (51) | Bangladesh | CS | Non-probabilistic, convenient sampling | Online survey | During | Lockdown | Jun 20-30, 2020 | GP, M: 1161 (57.2%)  F: 867 (42.8%), Mean age (SD): 25.9 (8.1)  Age range 18-65 | SF-IPAQ, Bangla | MVPA | 62.1 |
| Rahman, 2020 (51) | Bangladesh | CS | Non-probabilistic, convenient sampling | Online survey | During | Lockdown | Jun 20-30, 2020 | GP, M: 1161 (100%), Mean age (SD): 25.9 (8.1)  Age range 18-65 | SF-IPAQ, Bangla | MVPA | 61.1 |
| Rahman, 2020 (51) | Bangladesh | CS | Non-probabilistic, convenient sampling | Online survey | During | Lockdown | Jun 20-30, 2020 | GP, F: 867 (100%), Mean age (SD): 25.9 (8.1)  Age range 18-65 | SF-IPAQ, Bangla | MVPA | 63.6 |
| Rahman, 2020 (51) | Bangladesh | CS | Non-probabilistic, convenient sampling | Online survey | During | Lockdown | Jun 20-30, 2020 | Healthy, Mean age (SD): 25.9 (8.1)  Age range 18-65 | SF-IPAQ, Bangla | MVPA | 60 |
| Rahman, 2020 (51) | Bangladesh | CS | Non-probabilistic, convenient sampling | Online survey | During | Lockdown | Jun 20-30, 2020 | GP, M: 1169 (57.2%)  F: 868 (42.8%), Mean age (SD): 25.9 (8.1)  Age range 18-65 | SF-IPAQ, Bangla | Moderate | 38.3 |
| Rahman, 2020 (51) | Bangladesh | CS | Non-probabilistic, convenient sampling | Online survey | During | Lockdown | Jun 20-30, 2020 | GP, M: 1169 (57.2%)  F: 868 (42.8%), Mean age (SD): 25.9 (8.1)  Age range 18-65 | SF-IPAQ, Bangla | High | 23.9 |
| Rhodes, 2020 (52) | Canada | CS | Comparable with the Canadian census | Online and offline | During | Physical or social distancing | May 1-7, 2020 | GP, M: 517 (49%)  F: 538 (51%)  Unknown: 0.6%, Mean age (SD)=48.82 (16.66)  Age range:>18 | Godin Leisure-Time Questionnaire | MVPA | 37.5 |
| Rogowska, 2020 (53) | Ukraine | CS | Non-probabilistic, convenient sampling | Community | Before | No restriction | May 14-Jun 4, 2020 | GP, M: 474 (31.35%)  F: 1038 (68.65%), Mean age (SD)=20.06 (3.05)  Age range:18-51 | Online questionnaire | MVPA | 46.5 |
| Rogowska, 2020 (53) | Ukraine | CS | Non-probabilistic, convenient sampling | Community | During | During the first two months of the Corona Virus Disease 2019 (COVID-19) outbreak (Mar-Apr). Quarantine and lockdown, school closure and e-learning, State borders closed. Social distancing and the wearing of face masks were introduced as obligatory requirements. | May 14-Jun 4, 2020 | GP, M: 474 (31.35%)  F: 1038 (68.65%), Mean age (SD)=20.06 (3.05)  Age range:18-51 | Online questionnaire | MVPA | 43.2 |
| Sanudo, 2020 (54) | Spain | Longitudinal | NR | Online questionnaire | Before | No restrictions | Feb 2020 | GP, F: 9 (47)  M: NR | IPAQ- Spanish version | MVPA | 84 |
| Sanudo, 2020 (54) | Spain | Longitudinal | NR | Online questionnaire | During | Restrictions | Mar 24- Apr 3, 2020 | GP, F: 9 (47)  M: NR | IPAQ- Spanish version | MVPA | 74 |
| Silva, 2021(55) | Portugal | CS | Non-probabilistic sampling | Online & telephone survey | During | Social confinement | Apr 9- May 4, 2020 | GP, M: 3361 (57.4%)  F: 2495 (42.6%), Age range: >=16 years  Mean age: 45.8 years | IPAQ | Moderate | 20.5 |
| Silva, 2021(55) | Portugal | CS | Non-probabilistic sampling | Online & telephone survey | During | Social confinement | Apr 9- May 4, 2020 | GP, 49.9% men and 42.6% women, Age range: >=16 years  Mean age: 45.8 years | IPAQ | High | 46 |
| Silva, 2021(55) | Portugal | CS | Non-probabilistic sampling | Online & telephone survey | During | Social confinement | Apr 9- May 4, 2020 | GP, M: 100%, Age range: >=16 years  Mean age: 45.5 years | IPAQ | Moderate | 20.7 |
| Silva, 2021(55) | Portugal | CS | Non-probabilistic sampling | Online & telephone survey | During | Social confinement | Apr 9- May 4, 2020 | GP, M: 100%, Age range: >=16 years  Mean age: 45.5 years | IPAQ | High | 49.9 |
| Silva, 2021(55) | Portugal | CS | Non-probabilistic sampling | Online & telephone survey | During | Social confinement | Apr 9- May 4, 2020 | GP, F: 100%, Age range: >=16 years  Mean age: 46.2 years | IPAQ | Moderate | 20.3 |
| Silva, 2021(55) | Portugal | CS | Non-probabilistic sampling | Online & telephone survey | During | Social confinement | Apr 9- May 4, 2020 | GP, F: 100%, Age range: >=16 years  Mean age: 46.2 years | IPAQ | High | 42.6 |
| Silva, 2021 (55) | Portugal | CS | Non-probabilistic sampling | Online & telephone survey | During | Social confinement | Apr 9- May 4, 2020 | GP, M & F, Age range: 16-34 | IPAQ | Moderate | 18.6 |
| Silva, 2021(55) | Portugal | CS | Non-probabilistic sampling | Online & telephone survey | During | Social confinement | Apr 9- May 4, 2020 | GP, M & F, Age range: 16-34 | IPAQ | High | 53.9 |
| Silva, 2021(55) | Portugal | CS | Non-probabilistic sampling | Online & telephone survey | During | Social confinement | Apr 9- May 4, 2020 | GP, M & F, Age range:35-54 | IPAQ | Moderate | 16.3 |
| Silva, 2021(55) | Portugal | CS | Non-probabilistic sampling | Online & telephone survey | During | Social confinement | Apr 9- May 4, 2020 | GP, M & F, Age range:35-54 | IPAQ | High | 46.6 |
| Silva, 2021(55) | Portugal | CS | Non-probabilistic sampling | Online & telephone survey | During | Social confinement | Apr 9- May 4, 2020 | GP, M & F, Age range: >=55 | IPAQ | Moderate | 25.8 |
| Silva, 2021(55) | Portugal | CS | Non-probabilistic sampling | Online & telephone survey | During | Social confinement | Apr 9- May 4, 2020 | GP, M & F, Age range: >=55 | IPAQ | High | 39.4 |
| Slimani, 2020 (56) | Tunisia | CS | Non-probabilistic, convenient sampling | Online survey | During | 4 weeks after the implementation of the quarantine | NR | GP, M: 112 (51.9%)  F: 104 (48.1%), Mean age (SD)=27.9 (8.1) | SF-IPAQ, French | MVPA | 39.4 |
| Souza, 2021 (57) | Brazil | OS | Non-probabilistic sampling | Online survey | Before | No restriction (before Jan  2020) | Before Jan 2020 | GP, M:269 (19.7%)  F: 1094 (80%)  Unknown: 5 (0.3%), Age range: =>18  Median age (Q1-Q3): 31·0 (24·0–  39·0) | Online questionnaire | MVPA | 35.3 |
| Souza, 2021 (57) | Brazil | OS | Non-probabilistic sampling | Online survey | During | 5 months after the start of social distance measures implementation | Aug-Sep 2020 | GP, M:269 (19.7%)  F: 1094 (80%)  Unknown: 5 (0.3%), Age range: =>18  Median age (Q1-Q3): 31·0 (24·0–  39·0) | Online questionnaire | MVPA | 23.3 |
| Werneck, 2020 (58) | Brazil | CS | Non-probabilistic, chain sampling | Community | During | Before quarantine | Apr 24-May 24, 2020 | GP, M: 17241 (49.9%)  F: 17801 (50.8%), Age range:18-over 60 | No | MVPA | 31.2 |
| Wilke, 2020 (59) | 14 countries (Argentina, Australia, Austria, Brazil, Chile, France, Germany, Italy, the Netherlands, Singapore, South Africa, Spain, Switzerland, and the United States of America (USA) | CS | Non-probabilistic, convenient sampling | Online survey | During | Governmental restrictions limiting movement in public spaces | NR | GP, M: 6326 (41.1%)  F: 8935 (58.9%), Mean age (SD):38 (15)  Age range: 18-over 40 | Nordic Physical Activity Questionnaire, short version | MVPA | 81.5 |
| Zhang, 2020 (60) | China | Longitudinal study | Non-probabilistic, convenient sampling | Online survey | During | Forced to stay at home during the COVID-19 outbreak | Feb 19, 2020 | GP, M:25 (37.9%)  F: 41 (62.1%), Mean age (SD): 20.7 (2.11)  Age range: >18 | SF-IPAQ, Chinese | Moderate | 43.9 |
| Zhang, 2020 (60) | China | Longitudinal study | Non-probabilistic, convenient sampling | Online survey | During | Forced to stay at home during the COVID-19 outbreak | Feb 19, 2020 | GP, M:25 (37.9%)  F: 41 (62.1%), Mean age (SD): 20.7 (2.11)  Age range: >18 | SF-IPAQ, Chinese | High | 12.1 |
| Zhang, 2020 (60) | China | Longitudinal study | Non-probabilistic, convenient sampling | Online survey | During | Forced to stay at home during the COVID-19 outbreak | Feb 19, 2020 | GP, M:25 (100%), Age range: >18 | SF-IPAQ, Chinese | Moderate | 60 |
| Zhang, 2020 (60) | China | Longitudinal study | Non-probabilistic, convenient sampling | Online survey | During | Forced to stay at home during the COVID-19 outbreak | Feb 19, 2020 | GP, M:25 (100%), Age range: >18 | SF-IPAQ, Chinese | High | 24 |
| Zhang, 2020 (60) | China | Longitudinal study | Non-probabilistic, convenient sampling | Online survey | During | Forced to stay at home during the COVID-19 outbreak | Feb 19, 2020 | GP, F: 41 (62.1%), Age range: >18 | SF-IPAQ, Chinese | Moderate | 34.2 |
| Zhang, 2020 (60) | China | Longitudinal study | Non-probabilistic, convenient sampling | Online survey | During | Forced to stay at home during the COVID-19 outbreak | Feb 19, 2020 | GP, F: 41 (62.1%), Age range: >18 | SF-IPAQ, Chinese | High | 4.9 |
| Zheng, 2020 (61) | China | CS | Non-probabilistic, convenient sampling | Online survey | During | COVID-19 outbreak/closure of schools | Apr 15-26, 2020 | GP, M: 245 (38.8%)  F: 386 (61.2%), Mean age (SD): 21.1(2.9)  Age range: 18-35 | IPAQ | MVPA | 29.6 |
| Zheng, 2020 (61) | China | CS | Non-probabilistic, convenient sampling | Online survey | During | COVID-19 outbreak/closure of schools | Apr 15-26, 2020 | GP, M: 245 (100%), Mean age (SD): 21.5(3.2)  Age range: 18-35 | IPAQ | MVPA | 30.2 |
| Zheng, 2020 (61) | China | CS | Non-probabilistic, convenient sampling | Online survey | During | COVID-19 outbreak/closure of schools | Apr 15-26, 2020 | GP, F: 386 (100%), Mean age (SD): 20.9(2.5)  Age range: 18-35 | IPAQ | MVPA | 29.3 |

*PA among those not suffering from COVID-19 and adhering to a Mediterranean diet

NR-Not reported; CS-Cross-sectional; PA-Physical Activity; LOS-Longitudinal Observational study; GP-General population; M- Male; F-Female; MVPA-Moderate to Vigorous Physical Activity; IPAQ- International Physical Activity Questionnaire; SF-IPAQ- Short Form International Physical Activity Questionnaire; MLTPAQ- Minnesota Leisure Time Physical Activity Questionnaire

# **Table S6: Prevalence of Physical activity among adults with special conditions**

| **Reference** | | **Country** | | **Study design** | **Sampling method** | **Setting** | **Pre or during COVID19** | **Restrictions during the COVID19 Pandemic** | | **Data collection time** | **Population description** | **Physical activity instrument** | **Level of physical activity** | **Prevalence of physical activity (%)** |  |
| --- | --- | --- | --- | --- | --- | --- | --- | --- | --- | --- | --- | --- | --- | --- | --- |
| **Patients with COVID-19** | | | | | | | | | | | | | | | |
| Franco, 2020 (33) | | Spain | | Longitudinal | NR | Online Survey | Before | No restrictions | | Oct 2019 | COVID Patients,  M: 11 (64.7%)  F: 6(35.3%),  Mean age (SD): 42.76  (7.79) | IPAQ | MVPA | 88.2 |  |
| Franco, 2020 (33) | | Spain | | Longitudinal | NR | Online Survey | During | During confinement due to COVID19 restrictions | | May 2020 | COVID Patients,  M: 11 (64.7%)  F: 6(35.3%),  Mean age (SD): 42.76  (7.79) | IPAQ | MVPA | 17.6 |  |
| **Non-COVID-19 patients** | | | | | | | | | | | | | | | |
| Barrea, 2020 (62) | Italy | | CS | | Non-probabilistic, convenient sampling | Clinical | During | No restriction | Jan-Apr, 2020 | | Patients at the Department of Clinical Medicine and Surgery,  M: 43 (35.5%)  F: 78 (64.5%)  Mean age (SD): 44.9 (13.3)  Range: 18-65 | Telephonic interview | MVPA | 62.51 |  |
| Barrea, 2020 (62) | Italy | | CS | | Non-probabilistic, convenient sampling | Clinical | During | Emergency quarantine started in Mar 12, with stringent containment measures on the entire national  territory | Jan-Apr, 2020 | | Patients at the Department of Clinical Medicine and Surgery,  M: 43 (35.5%)  F: 78 (64.5%)  Mean age (SD): 44.9 (13.3)  Range: 18-65 | Telephonic interview | MVPA | 39.32 |  |
| Hashim, 2020 (63) | Iraq | | CS | | Non-probabilistic, convenient sampling | Clinical | During | Prolonged staying at home during the coronavirus disease 2019 (COVID-19) pandemic | Jan-Apr, 2020 | | Patients with unusual frequent urination associated with abnormal sleep,  M:19 (76%)  F: 6 (24%),  Mean age (SD): 28.24 (7.38)  Age range: 20-45 | Interview | MVPA | 0 |  |
| Rahman, 2020 (51) | Bangladesh | | CS | | Non-probabilistic, convenient sampling | Online survey | During | Lockdown | Jun 20-30, 2020 | | Patients with chronic Disease, Mean age (SD): 25.9 (8.1)  Age range 18-65 | SF-IPAQ, Bangla | MVPA | 71.4 |  |
| Reguera-García, 2020 (64) | Spain | | CS | | Non-probabilistic, convenient sampling | Online survey | During | Confinement | Mar 28- Jun, 2020 | | People with Multiple Sclerosis,  M: 34 (40.5%)  F: 50 (59.5%),  Mean age (SD): 46.9 (9.7) | SF-IPAQ, Spanish | Moderate | 34.5 |  |
| Reguera-García, 2020 (64) | Spain | | CS | | Non-probabilistic, convenient sampling | Online survey | During | Confinement | Mar 28- Jun, 2020 | | People with Multiple Sclerosis,  M: 34 (40.5%)  F: 50 (59.5%),  Mean age (SD): 46.9 (9.7) | SF-IPAQ, Spanish | High | 33.3 |  |
| Werneck, 2020 (58) | Brazil | | CS | | Non-probabilistic, chain sampling | Community | During | With restrictions | Apr 24-May 24, 2020 | | Patients with depression, M: 2188 (31.8%)  F: 4693 (68.2%), Age range:18-over 60 | Questionniare Survey | MVPA | 24.5 |  |

NR-Not reported; CS-Cross-sectional; M: Male; F-Female; MVPA-Moderate to Vigorous Physical Activity; IPAQ- International Physical Activity Questionnaire; SF-IPAQ- Short Form International Physical Activity Questionnaire

**Table S7: Quality assessment of the included studies**

| **References** | **Population**  **characteristics** | **Outcome**  **definition** | **Measurement**  **tool** | **Setting** | **Timing** | **Sampling**  **Method** | **Response**  **rate** |
| --- | --- | --- | --- | --- | --- | --- | --- |
| Aegerter, 2021(22) | Low ROB | Low ROB | Low ROB | Low ROB | Low ROB | High ROB | High ROB |
| Alonso-Martinez, 2021 (4) | Low ROB | Low ROB | Low ROB | Low ROB | Low ROB | High ROB | High ROB |
| Alsalhe, 2020 (23) | Low ROB | Low ROB | Low ROB | Low ROB | Low ROB | Moderate ROB | High ROB |
| Amini, 2020 (24) | Low ROB | Low ROB | Low ROB | Low ROB | Low ROB | Moderate ROB | Moderate ROB |
| Antunes, 2020 (25) | Low ROB | Low ROB | Low ROB | Low ROB | Low ROB | Moderate ROB | High ROB |
| Barrea, 2020 (62) | Low ROB | Low ROB | High ROB | Low ROB | Low ROB | Moderate ROB | High ROB |
| Boukrim, 2021 (26) | Low ROB | Low ROB | Low ROB | Low ROB | Low ROB | High ROB | High ROB |
| Bronikowska, 2021(5) | Low ROB | Low ROB | Low ROB | Low ROB | Low ROB | High ROB | Low ROB |
| Bourdas, 2020 (27) | Low ROB | Low ROB | Low ROB | Low ROB | Low ROB | Moderate ROB | High ROB |
| Brz˛ek, 2021(6) | Low ROB | Low ROB | High ROB | Low ROB | Low ROB | High ROB | High ROB |
| Carriedo, 2020 (28) | Low ROB | Low ROB | Low ROB | Low ROB | Moderate ROB | Moderate ROB | High ROB |
| Chaffee, 2021 (7) | Low ROB | Low ROB | High ROB | Low ROB | Low ROB | High ROB | High ROB |
| Chopra, 2020 (29) | Low ROB | Low ROB | High ROB | Low ROB | Low ROB | Moderate ROB | High ROB |
| Constantini, 2021 (8) | Low ROB | Low ROB | Low ROB | Low ROB | High ROB | Moderate ROB | High ROB |
| Crochemore-Silva, 2020 (30) | Low ROB | Low ROB | Low ROB | Low ROB | Low ROB | Low ROB | High ROB |
| de Matos ,2020 (20) | Low ROB | Low ROB | Low ROB | Low ROB | Low ROB | Moderate ROB | High ROB |
| Di Sebastiano, 2020 (31) | Low ROB | Low ROB | Low ROB | Low ROB | Low ROB | Moderate ROB | High ROB |
| Faulkner, 2020 (32) | Low ROB | Low ROB | Low ROB | Low ROB | Low ROB | Moderate ROB | High ROB |
| Francisco, 2020 (9) | Low ROB | Low ROB | High ROB | Low ROB | Low ROB | High ROB | High ROB |
| Franco, 2020 (33) | Low ROB | Low ROB | Low ROB | Low ROB | Low ROB | High ROB | High ROB |
| Fuentes-Garcia, 2020 (34) | Moderate ROB | Low ROB | High ROB | Low ROB | Low ROB | Moderate ROB | High ROB |
| Galle, 2020 (35) | Low ROB | Low ROB | Low ROB | Low ROB | Low ROB | Moderate ROB | High ROB |
| Giustino, 2020 (36) | Low ROB | Low ROB | Low ROB | Low ROB | Low ROB | Moderate ROB | High ROB |
| Hall-Lopez, 2020 (37) | Low ROB | Low ROB | Low ROB | Low ROB | High ROB | Moderate ROB | High ROB |
| Hashim, 2020 (63) | Low ROB | Low ROB | High ROB | Low ROB | Low ROB | Moderate ROB | High ROB |
| Hu, 2020 (38) | Low ROB | Low ROB | Low ROB | Low ROB | Low ROB | Moderate ROB | Moderate ROB |
| Jacob, 2020 (39) | Low ROB | Low ROB | High ROB | Low ROB | Low ROB | Moderate ROB | High ROB |
| Knell, 2020 (40) | Low ROB | Low ROB | Low ROB | Low ROB | Low ROB | Moderate ROB | Moderate ROB |
| Kovacs, 2021 (10) | Low ROB | Low ROB | High ROB | Low ROB | Low ROB | Moderate ROB | High ROB |
| Lesser, 2020 (65) | Low ROB | Low ROB | Low ROB | Low ROB | Low ROB | Moderate ROB | Low ROB |
| Lopez-Bueno, 2020 (66) | Low ROB | Low ROB | Low ROB | Low ROB | Low ROB | Moderate ROB | High ROB |
| Lopez-Bueno, 2020 (41) | Low ROB | Low ROB | Low ROB | Low ROB | Low ROB | Moderate ROB | Low ROB |
| Malta, 2020 (42) | Low ROB | Low ROB | High ROB | Low ROB | Low ROB | Moderate ROB | Low ROB |
| Martinez-de-Quel, 2020 (43) | Low ROB | Low ROB | Low ROB | Low ROB | Low ROB | Moderate ROB | Moderate ROB |
| Maugeri, 2020 (44) | Low ROB | Low ROB | Low ROB | Low ROB | Low ROB | Moderate ROB | High ROB |
| Medrano, 2020 (11) | Low ROB | Low ROB | Low ROB | Low ROB | Low ROB | Low ROB | High ROB |
| Meza, 2021 (45) | Low ROB | Low ROB | Low ROB | Low ROB | High ROB | Moderate ROB | High ROB |
| Mitra, 2020 (12) | Low ROB | Low ROB | High ROB | Low ROB | Low ROB | Moderate ROB | High ROB |
| Munasinghe, 2020 (13) | Low ROB | Low ROB | Low ROB | Low ROB | Low ROB | High ROB | Moderate ROB |
| Newby, 2020 (46) | Low ROB | Low ROB | Low ROB | Low ROB | Low ROB | Moderate ROB | Low ROB |
| Okely, 2021 (14) | Low ROB | Low ROB | Low ROB | Low ROB | Low ROB | Low ROB | Moderate ROB |
| Orgilés, 2020 (15) | Low ROB | Low ROB | Low ROB | Low ROB | Low ROB | Moderate ROB | High ROB |
| Orlandi, 2021 (47) | Low ROB | Low ROB | Low ROB | Low ROB | Low ROB | Moderate ROB | High ROB |
| Ozdemir, 2020 (48) | Low ROB | Low ROB | Low ROB | Low ROB | Low ROB | Moderate ROB | High ROB |
| Parker, 2021 (16) | Low ROB | Low ROB | Low ROB | Low ROB | Low ROB | Moderate ROB | High ROB |
| Qi, 2020 (49) | Low ROB | Low ROB | Low ROB | Low ROB | Low ROB | Moderate ROB | High ROB |
| Qin, 2020 (50) | Low ROB | Low ROB | Low ROB | Low ROB | Low ROB | Moderate ROB | High ROB |
| Rahman, 2020 (51) | Low ROB | Low ROB | Low ROB | Low ROB | Low ROB | Moderate ROB | High ROB |
| Reguera-Garcia, 2020 (64) | Low ROB | Low ROB | Low ROB | Low ROB | Low ROB | Moderate ROB | High ROB |
| Rhodes, 2020 (52) | Low ROB | Low ROB | Low ROB | Low ROB | Low ROB | Low ROB | High ROB |
| Rogowska, 2020 (53) | Low ROB | Low ROB | High ROB | Low ROB | Low ROB | Moderate ROB | High ROB |
| Ruiz-Roso, 2020 (17) | Low ROB | Low ROB | Low ROB | Low ROB | Low ROB | Moderate ROB | High ROB |
| Sanudo, 2020 (54) | Low ROB | Low ROB | Low ROB | Low ROB | Low ROB | High ROB | High ROB |
| Schmidt, 2020 (18) | Low ROB | Low ROB | Low ROB | Low ROB | Low ROB | Low ROB | Moderate ROB |
| Silva, 2021 (55) | Low ROB | Low ROB | Low ROB | Low ROB | Low ROB | Moderate ROB | High ROB |
| Slimani, 2020 (56) | Low ROB | Low ROB | Low ROB | Low ROB | Moderate ROB | Moderate ROB | Low ROB |
| Souza, 2021 (57) | Low ROB | Low ROB | High ROB | Low ROB | Low ROB | Moderate ROB | Low ROB |
| Werneck, 2020 (58) | Low ROB | Low ROB | High ROB | Low ROB | Low ROB | Moderate ROB | High ROB |
| Wilke, 2020 (59) | Low ROB | Low ROB | Low ROB | Low ROB | Moderate ROB | Moderate ROB | High ROB |
| Xiang, 2020 (19) | Low ROB | Low ROB | Low ROB | Low ROB | Low ROB | Low ROB | High ROB |
| Zhang, 2020 (60) | Low ROB | Low ROB | Low ROB | Low ROB | Low ROB | Moderate ROB | High ROB |
| Zheng, 2020 (61) | Low ROB | Low ROB | Low ROB | Low ROB | Low ROB | Moderate ROB | High ROB |
| Zhou, 2021 (21) | Low ROB | Low ROB | Low ROB | Low ROB | Low ROB | Moderate ROB | Low ROB |

ROB-Risk of Bias

# **Figure S1: Funnel plot for the during pandemic meta-analysis among adults**


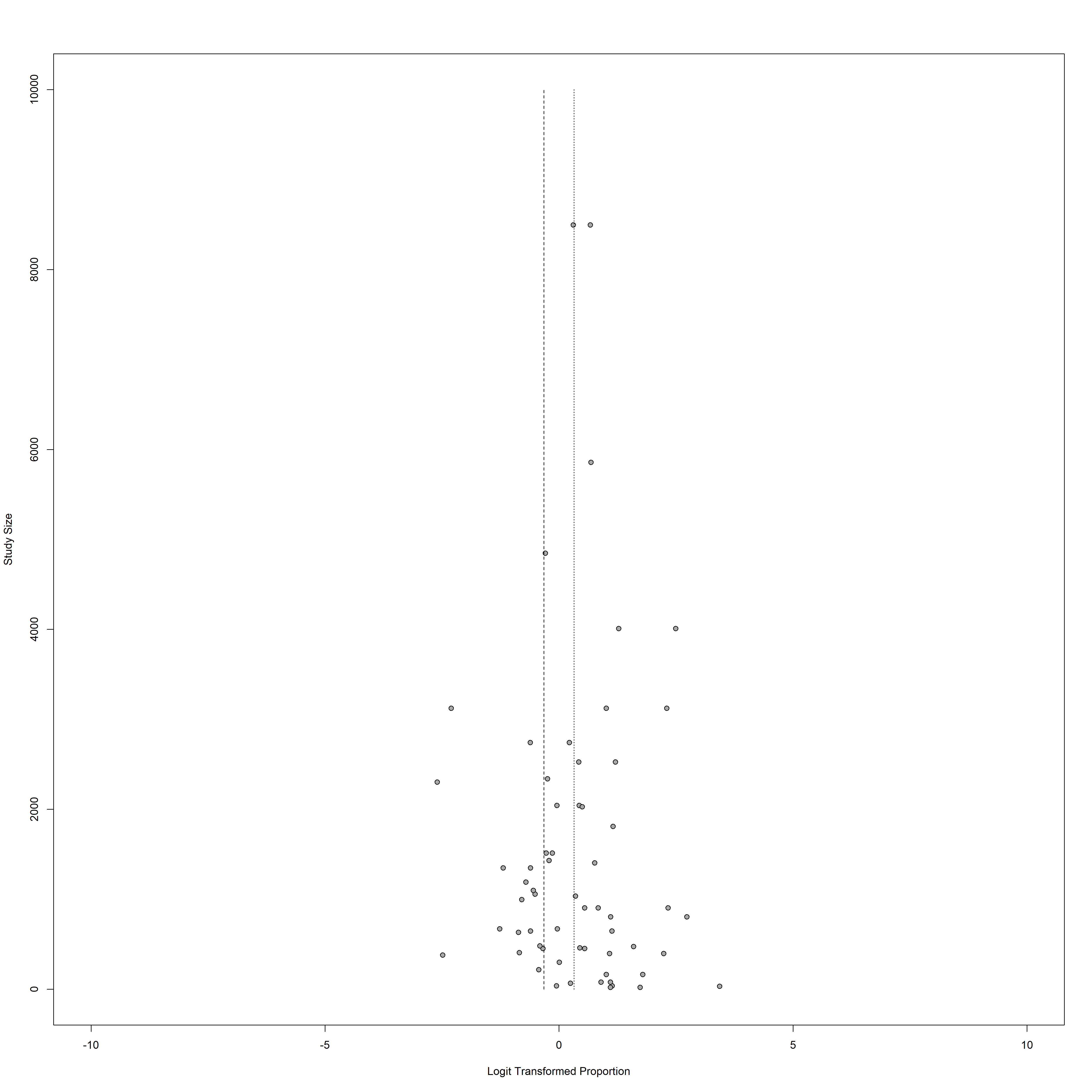


# **Figure S2: Funnel plot for the during pandemic meta-analysis among children**


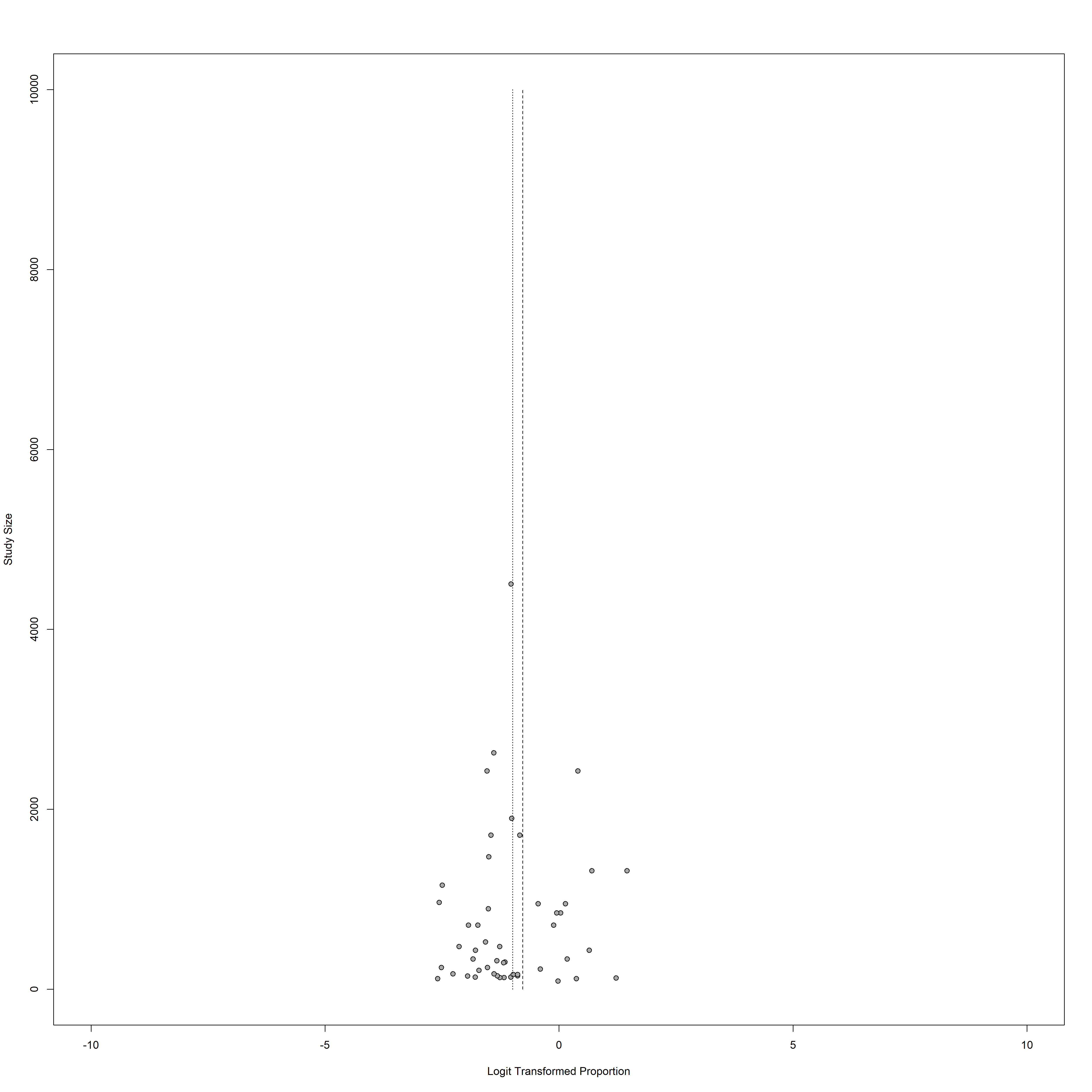


**References list**

1. Human energy requirements. Scientific background papers from the Joint FAO/WHO/UNU Expert Consultation. October 17-24, 2001. . Rome, Italy; 2005 Oct. Report No.: 1368-9800 (Print)

1368-9800 Contract No.: 7a.

2. World Health Organization (WHO). Global Recommendations on Physical Activity for Health. Geneva: World Health Organization 2010; 2010. Available from: <https://www.ncbi.nlm.nih.gov/books/NBK305049/>.

3. WHO-STEPS Survey Jordan. WHO STEPS survey Jordan, 2007. 2007.

4. Alonso-Martínez AM, Ramírez-Vélez R, García-Alonso Y, Izquierdo M, García-Hermoso A. Physical Activity, Sedentary Behavior, Sleep and Self-Regulation in Spanish Preschoolers during the COVID-19 Lockdown. Int J Environ Res Public Health. 2021;18(2).

5. Bronikowska M, Krzysztoszek J, Łopatka M, Ludwiczak M, Pluta B. Comparison of Physical Activity Levels in Youths before and during a Pandemic Lockdown. Int J Environ Res Public Health. 2021;18(10).

6. Brzęk A, Strauss M, Sanchis-Gomar F, Leischik R. Physical Activity, Screen Time, Sedentary and Sleeping Habits of Polish Preschoolers during the COVID-19 Pandemic and WHO's Recommendations: An Observational Cohort Study. Int J Environ Res Public Health. 2021;18(21).

7. Chaffee BW, Cheng J, Couch ET, Hoeft KS, Halpern-Felsher B. Adolescents' Substance Use and Physical Activity Before and During the COVID-19 Pandemic. JAMA Pediatr. 2021;175(7):715-22.

8. Constantini K, Markus I, Epel N, Jakobovich R, Gepner Y, Lev-Ari S. Continued Participation of Israeli Adolescents in Online Sports Programs during the COVID-19 Pandemic Is Associated with Higher Resilience. Int J Environ Res Public Health. 2021;18(8).

9. Francisco R, Pedro M, Delvecchio E, Espada JP, Morales A, Mazzeschi C, et al. Psychological Symptoms and Behavioral Changes in Children and Adolescents During the Early Phase of COVID-19 Quarantine in Three European Countries. Front Psychiatry. 2020;11:570164.

10. Kovacs VA, Starc G, Brandes M, Kaj M, Blagus R, Leskošek B, et al. Physical activity, screen time and the COVID-19 school closures in Europe - An observational study in 10 countries. Eur J Sport Sci. 2021:1-10.

11. Medrano M, Cadenas-Sanchez C, Oses M, Arenaza L, Amasene M, Labayen I. Changes in lifestyle behaviours during the COVID-19 confinement in Spanish children: A longitudinal analysis from the MUGI project. Pediatr Obes. 2020:e12731.

12. Mitra R, Moore SA, Gillespie M, Faulkner G, Vanderloo LM, Chulak-Bozzer T, et al. Healthy movement behaviours in children and youth during the COVID-19 pandemic: Exploring the role of the neighbourhood environment. Health Place. 2020;65:102418.

13. Munasinghe S, Sperandei S, Freebairn L, Conroy E, Jani H, Marjanovic S, et al. The Impact of Physical Distancing Policies During the COVID-19 Pandemic on Health and Well-Being Among Australian Adolescents. J Adolesc Health. 2020;67(5):653-61.

14. Okely AD, Kariippanon KE, Guan H, Taylor EK, Suesse T, Cross PL, et al. Global effect of COVID-19 pandemic on physical activity, sedentary behaviour and sleep among 3- to 5-year-old children: a longitudinal study of 14 countries. BMC Public Health. 2021;21(1):940.

15. Orgilés M, Morales A, Delvecchio E, Mazzeschi C, Espada JP. Immediate Psychological Effects of the COVID-19 Quarantine in Youth From Italy and Spain. Front Psychol. 2020;11:579038.

16. Parker K, Uddin R, Ridgers ND, Brown H, Veitch J, Salmon J, et al. The Use of Digital Platforms for Adults' and Adolescents' Physical Activity During the COVID-19 Pandemic (Our Life at Home): Survey Study. J Med Internet Res. 2021;23(2):e23389.

17. Ruíz-Roso MB, de Carvalho Padilha P, Matilla-Escalante DC, Brun P, Ulloa N, Acevedo-Correa D, et al. Changes of Physical Activity and Ultra-Processed Food Consumption in Adolescents from Different Countries during Covid-19 Pandemic: An Observational Study. Nutrients. 2020;12(8).

18. Schmidt SCE, Anedda B, Burchartz A, Eichsteller A, Kolb S, Nigg C, et al. Physical activity and screen time of children and adolescents before and during the COVID-19 lockdown in Germany: a natural experiment. Sci Rep. 2020;10(1):21780.

19. Xiang M, Zhang Z, Kuwahara K. Impact of COVID-19 pandemic on children and adolescents&#039; lifestyle behavior larger than expected. Prog Cardiovasc Dis. 2020;63(4):531-2.

20. de Matos GD, Aidar F, de Almeida-Neto PF, Moreira OC, de Souza RF, Marcal AC, et al. The Impact of Measures Recommended by the Government to Limit the Spread of Coronavirus (COVID-19) on Physical Activity Levels, Quality of Life, and Mental Health of Brazilians. Sustainability. 2020;12(21):9072.

21. Zhou J, Xie X, Guo B, Pei R, Pei X, Yang S, et al. Impact of COVID-19 Lockdown on Physical Activity Among the Chinese Youths: The COVID-19 Impact on Lifestyle Change Survey (COINLICS). Front Public Health. 2021;9:592795.

22. Aegerter AM, Deforth M, Sjøgaard G, Johnston V, Volken T, Luomajoki H, et al. No Evidence for a Decrease in Physical Activity Among Swiss Office Workers During COVID-19: A Longitudinal Study. Front Psychol. 2021;12:620307.

23. Alsalhe TA, Aljaloud SO, Chalghaf N, Guelmami N, Alhazza DW, Azaiez F, et al. Moderation Effect of Physical Activity on the Relationship Between Fear of COVID-19 and General Distress: A Pilot Case Study in Arabic Countries. Front Psychol. 2020;11:570085.

24. Amini H, Isanejad A, Chamani N, Movahedi-Fard F, Salimi F, Moezi M, et al. Physical activity during COVID-19 Pandemic in the Iranian population: A brief report. Heliyon. 2020:e05411.

25. Antunes R, Frontini R, Amaro N, Salvador R, Matos R, Morouço P, et al. Exploring Lifestyle Habits, Physical Activity, Anxiety and Basic Psychological Needs in a Sample of Portuguese Adults during COVID-19. Int J Environ Res Public Health. 2020;17(12).

26. Boukrim M, Obtel M, Kasouati J, Achbani A, Razine R. Covid-19 and Confinement: Effect on Weight Load, Physical Activity and Eating Behavior of Higher Education Students in Southern Morocco. Ann Glob Health. 2021;87(1):7.

27. Bourdas DI, Zacharakis ED. Impact of COVID-19 Lockdown on Physical Activity in a Sample of Greek Adults. Sports (Basel). 2020;8(10).

28. Carriedo A, Cecchini JA, Fernandez-Rio J, Méndez-Giménez A. COVID-19, Psychological Well-being and Physical Activity Levels in Older Adults During the Nationwide Lockdown in Spain. Am J Geriatr Psychiatry. 2020;28(11):1146-55.

29. Chopra S, Ranjan P, Singh V, Kumar S, Arora M, Hasan MS, et al. Impact of COVID-19 on lifestyle-related behaviours- a cross-sectional audit of responses from nine hundred and ninety-five participants from India. Diabetes Metab Syndr. 2020;14(6):2021-30.

30. Crochemore-Silva I, Knuth AG, Wendt A, Nunes BP, Hallal PC, Santos LP, et al. Physical activity during the COVID-19 pandemic: a population-based cross-sectional study in a city of South Brazil. Cien Saude Colet. 2020;25(11):4249-58.

31. Di Sebastiano KM, Chulak-Bozzer T, Vanderloo LM, Faulkner G. Don't Walk So Close to Me: Physical Distancing and Adult Physical Activity in Canada. Front Psychol. 2020;11:1895.

32. Faulkner J, O'Brien W, McGrane B, Wadsworth D, Batten J, Askew CD, et al. Physical activity, mental health and well-being of adults during early COVID-19 containment strategies: A multi-country cross-sectional analysis. medRxiv. 2020:2020.07.15.20153791.

33. Franco E, Urosa J, Barakat R, Refoyo I. Physical Activity and Adherence to the Mediterranean Diet among Spanish Employees in a Health-Promotion Program before and during the COVID-19 Pandemic: The Sanitas-Healthy Cities Challenge. Int J Environ Res Public Health. 2021;18(5).

34. Fuentes-García JP, Martínez Patiño MJ, Villafaina S, Clemente-Suárez VJ. The Effect of COVID-19 Confinement in Behavioral, Psychological, and Training Patterns of Chess Players. Front Psychol. 2020;11:1812.

35. Gallè F, Sabella EA, Ferracuti S, De Giglio O, Caggiano G, Protano C, et al. Sedentary Behaviors and Physical Activity of Italian Undergraduate Students during Lockdown at the Time of CoViD-19 Pandemic. Int J Environ Res Public Health. 2020;17(17).

36. Giustino V, Parroco AM, Gennaro A, Musumeci G, Palma A, Battaglia G. Physical Activity Levels and Related Energy Expenditure during COVID-19 Quarantine among the Sicilian Active Population: A Cross-Sectional Online Survey Study. Sustainability. 2020;12(11):4356.

37. Hall-López JA. Physical activity levels in physical education teachers before and during school suspension brought by the covid-19 quarantine. Facta Universitatis, Series: Physical Education and Sport. 2020:475-81.

38. Hu Z, Lin X, Chiwanda Kaminga A, Xu H. Impact of the COVID-19 Epidemic on Lifestyle Behaviors and Their Association With Subjective Well-Being Among the General Population in Mainland China: Cross-Sectional Study. J Med Internet Res. 2020;22(8):e21176-e.

39. Jacob L, Tully MA, Barnett Y, Lopez-Sanchez GF, Butler L, Schuch F, et al. The relationship between physical activity and mental health in a sample of the UK public: A cross-sectional study during the implementation of COVID-19 social distancing measures. Mental Health and Physical Activity. 2020;19(100345).

40. Knell G, Robertson MC, Dooley EE, Burford K, Mendez KS. Health Behavior Changes During COVID-19 Pandemic and Subsequent "Stay-at-Home" Orders. Int J Environ Res Public Health. 2020;17(17).

41. López-Bueno R, Calatayud J, Casaña J, Casajús JA, Smith L, Tully MA, et al. COVID-19 Confinement and Health Risk Behaviors in Spain. Front Psychol. 2020;11:1426.

42. Malta DC, Szwarcwald CL, Barros MBA, Gomes CS, Machado Í E, Souza Júnior PRB, et al. The COVID-19 Pandemic and changes in adult Brazilian lifestyles: a cross-sectional study, 2020. Epidemiol Serv Saude. 2020;29(4):e2020407.

43. Martínez-de-Quel Ó, Suárez-Iglesias D, López-Flores M, Pérez CA. Physical activity, dietary habits and sleep quality before and during COVID-19 lockdown: A longitudinal study. Appetite. 2020;158:105019.

44. Maugeri G, Castrogiovanni P, Battaglia G, Pippi R, D'Agata V, Palma A, et al. The impact of physical activity on psychological health during Covid-19 pandemic in Italy. Heliyon. 2020;6(6):e04315.

45. Meza EIA, López JAH. Physical activity in university student athletes, prior and in confinement due to pandemic associated with COVID-19. Retos: nuevas tendencias en educación física, deporte y recreación. 2021(39):572-5.

46. Newby JM, O'Moore K, Tang S, Christensen H, Faasse K. Acute mental health responses during the COVID-19 pandemic in Australia. PLoS One. 2020;15(7):e0236562.

47. Orlandi M, Rosselli M, Pellegrino A, Boddi M, Stefani L, Toncelli L, et al. Gender differences in the impact on physical activity and lifestyle in Italy during the lockdown, due to the COVID-19 pandemic. Nutrition, Metabolism and Cardiovascular Diseases. 2021;31(7):2173-80.

48. Ozdemir F, Cansel N, Kizilay F, Guldogan E, Ucuz I, Sinanoglu B, et al. The role of physical activity on mental health and quality of life during COVID-19 outbreak: A cross-sectional study. Eur J Integr Med. 2020:101248.

49. Qi M, Li P, Moyle W, Weeks B, Jones C. Physical Activity, Health-Related Quality of Life, and Stress among the Chinese Adult Population during the COVID-19 Pandemic. Int J Environ Res Public Health. 2020;17(18).

50. Qin F, Song Y, Nassis GP, Zhao L, Dong Y, Zhao C, et al. Physical Activity, Screen Time, and Emotional Well-Being during the 2019 Novel Coronavirus Outbreak in China. Int J Environ Res Public Health. 2020;17(14).

51. Rahman ME, Islam MS, Bishwas MS, Moonajilin MS, Gozal D. Physical inactivity and sedentary behaviors in the Bangladeshi population during the COVID-19 pandemic: An online cross-sectional survey. Heliyon. 2020;6(10):e05392.

52. Rhodes RE, Liu S, Lithopoulos A, Zhang CQ, Garcia-Barrera MA. Correlates of Perceived Physical Activity Transitions during the COVID-19 Pandemic among Canadian Adults. Appl Psychol Health Well Being. 2020.

53. Rogowska AM, Pavlova I, Kusnierz C, Ochnik D, Bodnar I, Petrytsa P. Does physical activity matter for the mental health of university students during the COVID-19 pandemic? Journal of Clinical Medicine. 2020;9(11):1-19.

54. Sanudo B, Curtis F, Sanchez-Oliver A. Objectively-Assessed Physical Activity, Sedentary Behavior, Smartphone Use, and Sleep Patterns Pre- and during-COVID-19 Quarantine in Young Adults from Spain. Sustainability. 2020;12(15):5890.

55. Silva MN, Gregório MJ, Santos R, Marques A, Rodrigues B, Godinho C, et al. Towards an In-Depth Understanding of Physical Activity and Eating Behaviours during COVID-19 Social Confinement: A Combined Approach from a Portuguese National Survey. Nutrients. 2021;13(8).

56. Slimani M, Paravlic A, Mbarek F, Bragazzi NL, Tod D. The Relationship Between Physical Activity and Quality of Life During the Confinement Induced by COVID-19 Outbreak: A Pilot Study in Tunisia. Front Psychol. 2020;11:1882.

57. Souza TC, Oliveira LA, Daniel MM, Ferreira LG, Della Lucia CM, Liboredo JC, et al. Lifestyle and eating habits before and during COVID-19 quarantine in Brazil. Public Health Nutr. 2021;25(1):65-75.

58. Werneck AO, Silva DRD, Malta DC, Souza-Júnior PRB, Azevedo LO, Barros MBA, et al. Lifestyle behaviors changes during the COVID-19 pandemic quarantine among 6,881 Brazilian adults with depression and 35,143 without depression. Cien Saude Colet. 2020;25:4151-6.

59. Wilke J, Mohr L, Tenforde AS, Edouard P, Fossati C, González-Gross M, et al. Restrictercise! Preferences Regarding Digital Home Training Programs during Confinements Associated with the COVID-19 Pandemic. Int j environ res public health (Online). 2020;17(18).

60. Zhang Y, Zhang H, Ma X, Di Q. Mental Health Problems during the COVID-19 Pandemics and the Mitigation Effects of Exercise: A Longitudinal Study of College Students in China. Int j environ res public health (Online). 2020;17(10).

61. Zheng C, Huang WY, Sheridan S, Sit CH-P, Chen X-K, Wong SH-S. COVID-19 Pandemic Brings a Sedentary Lifestyle in Young Adults: A Cross-Sectional and Longitudinal Study. Int j environ res public health (Online). 2020;17(17).

62. Barrea L, Pugliese G, Framondi L, Di Matteo R, Laudisio D, Savastano S, et al. Does Sars-Cov-2 threaten our dreams? Effect of quarantine on sleep quality and body mass index. J Transl Med. 2020;18(1):318-.

63. Hashim HT. Association Between Frequent Urination and Prolonged Staying at Home. Prim Care Companion CNS Disord. 2020;22(5).

64. Reguera-García MM, Liébana-Presa C, Álvarez-Barrio L, Alves Gomes L, Fernández-Martínez E. Physical Activity, Resilience, Sense of Coherence and Coping in People with Multiple Sclerosis in the Situation Derived from COVID-19. Int J Environ Res Public Health. 2020;17(21).

65. Lesser IA, Nienhuis CP. The Impact of COVID-19 on Physical Activity Behavior and Well-Being of Canadians. Int J Environ Res Public Health. 2020;17(11).

66. López-Bueno R, Calatayud J, Andersen LL, Balsalobre-Fernández C, Casaña J, Casajús JA, et al. Immediate Impact of the COVID-19 Confinement on Physical Activity Levels in Spanish Adults. Sustainability. 2020;12(14):5708.
